# Revisiting Zn-specific nucleation via a dimensionless factor to quantify interfacial electrochemistry of aqueous batteries

**DOI:** 10.1038/s41467-026-73953-w

**Published:** 2026-06-02

**Authors:** Zeyu Wang, Wanhai Zhou, Gaoyang Li, Zhuo Yang, Zefang Yang, Yuhang Liu, Tengsheng Zhang, Hongrun Jin, Shixiang Ding, Junwei Zhang, Xia Wang, Fanxing Bu, Min Wang, Jingwen Zhao, Zaiwang Zhao, Dongyuan Zhao, Dongliang Chao

**Affiliations:** 1 Laboratory of Advanced Materials, Aqueous Battery Center, Shanghai Key Laboratory of Molecular Catalysis and Innovative Materials, Collaborative Innovation Center of Chemistry for Energy Materials, Shanghai Wusong Laboratory of Materials Science, State Key Laboratory of Porous Materials for Separation and Conversion, College of Smart Materials and Future Energy, Fudan University, Shanghai, China; 2 Qingdao Industrial Energy Storage Research Institute, Qingdao Institute of Bioenergy and Bioprocess Technology, Chinese Academy of Sciences, Qingdao, China; 3 College of Energy Materials and Chemistry, College of Chemistry and Chemical Engineering, Inner Mongolia University, Hohhot, China

**Keywords:** Batteries, Energy transfer, Batteries

## Abstract

Zn-based aqueous batteries have attracted widespread research attention, while the lack of nucleation theory for electrochemical interactions at the Zn-water interface constrains efforts to suppress the thermodynamically spontaneous hydrogen evolution reaction and dendrite formation, thereby stalling practical development. Elucidating Zn electrodeposition in aqueous media requires Zn-specific nucleation theory and a descriptor to regulate interfacial electrochemistry. Conventional Li-based spherical nucleation models disregard Zn’s crystallography and the interfacial resistance that governs nucleation, thereby focusing on polarization variations. In this work, we reformulate the classical spherical nucleation theory derived by Li for the hexagonal close-packed structure of Zn and establish a dimensionless descriptor (*W*_f_) to quantitatively rationalize interfacial electrochemistry. *W*_f_ synthesizes the polarization driving force and interfacial resistance into a stability metric. Higher *W*_f_ values facilitate uniform Zn deposition, as evidenced by the literature. Accordingly, we develop a high-*W*_f_ electrolyte to inhibit dendrites and side reactions, achieving over 700 h at 100% depth of discharge and 7660 cycles at 10 A g^−1^ in a Zn||NaV_3_O_8_ cell. This work provides a fundamental nucleation theory and a generally applicable quantitative metric for the rational design of Zn-based aqueous batteries.

## Abstract (web summary)

Zinc-based batteries offer safe energy storage but are limited by damaging dendrite growth. Here, authors report a zinc-specific nucleation model and a stability metric to design an advanced electrolyte that prevents dendrites, yielding highly stable, long-lasting batteries.

## Introduction

Zn-based aqueous batteries (ZABs) are promising for safe, low-cost energy storage^1–3^, but uncontrolled Zn deposition can lead to dendrite growth and corrosion, severely restricting their use^4,5^. Despite extensive efforts, such as electrolyte optimization and interfacial engineering^6–16^, a fundamental understanding of Zn electrode stabilization remains elusive. The absence of a unified theoretical framework to describe the dynamic evolution of the Zn-electrolyte interface during electrodeposition continues to thwart mechanistic understanding and rational interfacial design^17–19^. Although classical nucleation theory (CNT) is widely used to describe Zn electrodeposition^20–22^, its assumption of spherical nucleation neglects the intrinsic hexagonal close-packed (HCP) structure of Zn. This oversimplification compromises the accurate prediction of nucleation behavior^23,24^. Consequently, a comprehensive nucleation theory that accurately captures Zn-electrolyte interfacial dynamics during deposition is imperative.

Initial nucleation and growth processes govern long-term electrode stability^25^. These stages demand a fundamental understanding of Zn electrodeposition at the dynamic solid-liquid interface, where ion transport, interfacial charge transfer, and non-equilibrium processes interplay^26,27^. While CNT attributes metal nucleation to the relationship between interfacial polarization and surface energy, previous studies have frequently underestimated the decisive role of surface energy in dictating interfacial behavior^28^. The pronounced direction-dependent surface energy of Zn promotes non-spherical nucleation during the early growth stage, necessitating a HCP model of the nucleation^29,30^. The low surface energy of the (002) basal plane directs Zn^2+^ deposition along low-energy facets, leading to anisotropic HCP growth^31,32^. Furthermore, the theoretical driving force facilitates nucleation only when interfacial charge and mass transport are sufficiently accessible, whereas kinetic barriers and parasitic reactions consume a portion of this energy^33^. Specifically, at the Zn-water interface, intrinsic hydrogen evolution and corrosion precipitate byproducts, reducing the efficiency of Zn plating and stripping. These processes also promote two-dimensional growth, leading to extended sheets that can penetrate separators and trigger short circuits, further limiting the lifetime of ZABs^4^. Therefore, a unified descriptor is essential to quantitatively encapsulate the collaborative influence of interfacial kinetics and thermodynamics on nucleation behavior for stable and compact Zn deposition.

In this work, a Zn-HCP nucleation model and a generally applicable quantitative metric are established to close the existing theory gap. CNT is extended to incorporate Zn-HCP crystallography by replacing the spherical nucleation approximation with hexagonal platelets that account for anisotropic growth. The resulting model quantitatively correlates critical nucleus size, nucleation energy barrier, overpotential, and surface energy. To facilitate the assessment of nucleation kinetics under practical conditions, a dimensionless descriptor, *W*_f_, is defined. This metric integrates the electrodeposition driving force (overpotential) and interfacial resistance (corrosion current and polarization resistance), enabling a direct evaluation of deposition efficiency. A higher *W*_f_ indicates that the electrodeposition driving force dominates the interfacial resistance, which promotes uniform Zn growth, as corroborated by previously reported data. Guided by this metric, a high-*W*_f_ (HW) electrolyte is formulated to suppress dendrite formation and parasitic reactions effectively. The elaborated electrolyte delivers > 700 h at 100% DOD, 7660 cycles in Zn||NaV_3_O_8_ cells, and 97% retention over 200 cycles in the pouch cells (N/P = 2.62, areal capacity = 2.25 mAh cm^−2^). This work provides a theoretical foundation and a general, quantitative design criterion for Zn nucleation theory in ZABs.

## Results

### A Zn-HCP nucleation theory

The CNT, from a thermodynamic perspective, describes the barrier to crystal formation at the solid-liquid interface^34^. It expresses the free-energy difference (∆*G*) as the surface free energy (Δ*G*_s_) minus the bulk free energy (Δ*G*_v_), reflecting the fundamental competition between surface and volume contributions (Eq. (1))^35,36^.

1 $$\Delta G=-\Delta {G}_{{\rm{v}}}+\Delta {G}_{{\rm{s}}}=-V\Delta {g}_{{\rm{v}}}+S{\rm{\gamma }}$$

Here, *V* and *S* denote the model volume and surface area, respectively. The parameter *γ* represents the surface energy between the new phase and the environment, and ∆*g*_v_ signifies the free energy change per unit volume. Metal nucleation requires surmounting a thermodynamic barrier, where the maximum value (∆G^*^) determines the critical nucleus size^37^. However, the spherical nucleation approximation in CNT is inadequate for characterizing the anisotropic^38^, quasi-two-dimensional nucleation of Zn, which contrasts with the isotropic spherical growth of Li^39^. This direction-dependent growth accelerates dendrite formation and shortens battery lifespan, highlighting the necessity for a Zn-specific model.

**Fig. 1:**
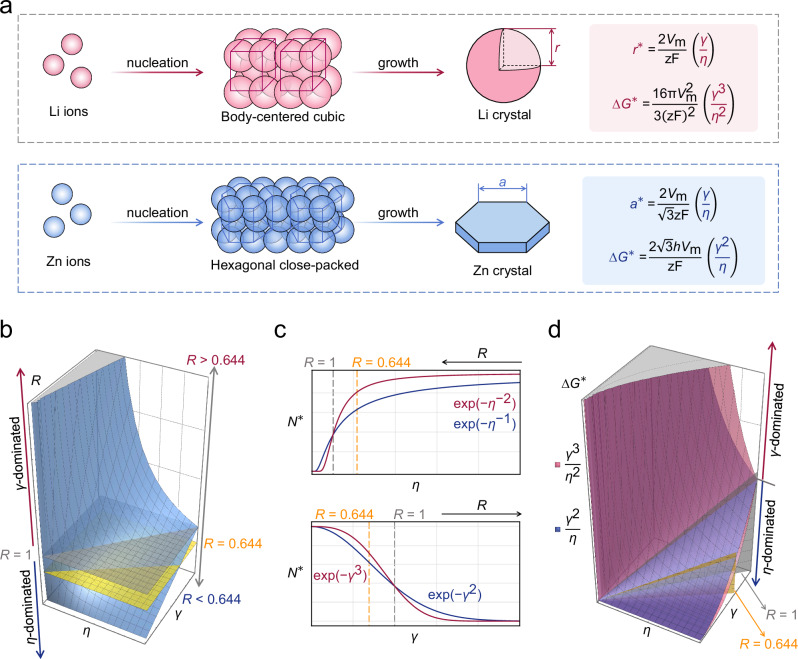
A Zn-HCP nucleation theory. **a** Schematic illustration of the classical nucleation theory (CNT) applied to Li (red) and Zn (blue) nucleation processes. **b** Three-dimensional plot (blue) of the ratio *R* as a function of *γ* and *η*, showing its rate variation along the *R* = 0.644 plane (yellow) and the *R* = 1 plane (gray). **c** Dependence of N^*^ on *γ* or *η* under different nucleation models. The yellow line (*R* = 0.644) partitions the rate of change, and the gray line (*R* = 1) defines the *η*-dominated and *γ*-dominated regions, with red and blue curves representing spherical and hexagonal close-packed (HCP) nucleation, respectively. **d** Three-dimensional plots of (*γ*^2^ / *η*) (blue-violet) and (*γ*^3^ / *η*^2^) (plum) as functions of *γ* or *η*, showing the corresponding rate trends.

Metals exhibit distinct nucleation behaviours arising from crystallographic anisotropy in surface energy (*γ*) (Fig. 1a)^40^. For body-centered cubic (BCC) Li, *γ* is relatively isotropic (*γ* ≈ 29–35 meV Å^−2^ across facets), favoring spherical nucleation and uniform growth^41^. To address the limitations of existing models that neglect crystallographic anisotropy^23,24^, a revised Zn-HCP nucleation mechanism is proposed here, governed by facet-dependent *γ*. In this framework, the (001) basal plane exhibits the lowest *γ* (21 meV Å^−2^, Supplementary Fig. 1). In contrast, higher-energy facets such as (100) and (101) drive anisotropic growth. This direction-dependent behavior leads to HCP nucleation and quasi-two-dimensional Zn deposition, which is distinct from the isotropic Li system (Supplementary Fig. 2)^42,43^. Based on the spherical and HCP nucleation models, where *r*, *a*, and *h* denote the radius, edge length, and height, respectively, the corresponding Δ*G* expressions are derived in Eqs (2) and (3).

2 $$\Delta {G}_{{\rm{spherical}}}=-\frac{4}{3}{\rm{\pi }}{r}^{3}\Delta {g}_{{\rm{v}}}+4{\rm{\pi }}{r}^{2}\gamma$$

3 $$\Delta {G}_{{\rm{hexagonal}}}=3\sqrt{3}{a}^{2}\left(\gamma -\frac{h\Delta {g}_{{\rm{v}}}}{2}\right)+6{ah}\gamma$$

By differentiating Δ*G* with respect to the geometric parameters and setting the derivative to zero, the extremum (critical free-energy difference, ΔG^*^) and corresponding critical sizes are obtained (Supplementary Eqs. (10) and (11)). For the HCP nucleation, assuming a constant height under overpotential (*η*)-dominated deposition (*g*_v_ ≫ *γ*), the analytical expressions in Eq. (4) constitute a thermodynamic framework for quantifying the critical nucleus length (a^*^), critical free-energy difference (∆G^*^, Eq. (5)), and critical nuclei density (N^*^, Supplementary Equation (17)). The a^*^ scales linearly with *γ* and inversely with *η* (Supplementary Fig. 3), where the proportionality constant encompasses the Zn^2+^ charge (*z* = 2), molar volume (*V*_m_), and the Faraday constant (F).

4 $${a}^{*}=\frac{2{V}_{{\rm{m}}}}{\sqrt{3}{\rm{zF}}}\left(\frac{\gamma }{\eta }\right)$$

5 $$\Delta {G}_{{\rm{hexagonal}}}^{*}=\frac{2\sqrt{3}h{V}_{{\rm{m}}}}{{\rm{zF}}}\left(\frac{{\gamma }^{2}}{\eta }\right)$$

As depicted in Supplementary Fig. 4, the ∆G^*^ of HCP nucleation increases with *γ*^2^ but decreases with *η* (Eq. (5)), whereas N^*^ is sensitive to both parameters. Spherical nucleation follows similar trends, where ∆G^*^ is proportional to *γ*^3^ and *η*^−2^ (Supplementary Equation (13)). To evaluate these trends, the variation of ∆G^*^ between the two models is quantified. Accordingly, the ratio *R* = (*γ*^3^/*η*^2^) / (*γ*^2^/*η*) (Eq. (6)) is evaluated, which yields the three-dimensional correlation in Fig. 1b. As illustrated, *R* = 1 serves as the boundary between the overpotential-dominated (*γ* < *η*) and surface-energy-dominated (*γ* > *η*) nucleation regimes. The simulated trends of N^*^ with respect to *η* and *γ* for both nucleation models are presented in Fig. 1c.

6 $$R\left(\gamma,\eta \right)=\frac{\gamma }{\eta }$$

To elucidate the relationship between the ratio *R* and the variation rates of ∆G^*^ in both nucleation models, the relative and absolute variation rates of *B* = *γ*^3^/*η*^2^ and *A* = *γ*^2^/*η* are analyzed (derivation in Supplementary Note 2).

7 $$\frac{\left|\nabla A\right|}{\left|\nabla B\right|}=\frac{1}{R}\sqrt{\frac{4+{R}^{2}}{9+4{R}^{2}}}$$

The examination of Eq. (7) demonstrates the variation behavior of (*γ*^3^ / *η*^2^) and (*γ*^2^ / *η*) (Supplementary Fig. 5). A positive real solution *R* = 0.644 is obtained under the condition |∇*A*|/|∇*B*| = 1. This value identifies a clear threshold for the relative behavior of the two nucleation modes. For *R* < 0.644, HCP nucleation exhibits a stronger dependence on *η*, as both the ∆G^*^ and N^*^ evolve rapidly with increasing *η*. Furthermore, N^*^ exhibits an exponential decay, which indicates a precipitous decrease (Fig. 1d). In the intermediate range 0.644 < *R* < 1, the spherical nucleation mode varies more quickly than the HCP mode (Fig. 1d). When the nucleation process is dominated by *γ* (*R* ≥ 1), this trend continues and the rates of change of ∆G^*^ and N^*^ in the spherical nucleus accelerate significantly with increasing *R*.

The stability of metal nucleation and subsequent growth is governed by the critical nucleus size (*a* or *r*) and the N^*^. Schematic illustrations of these specific variations are summarized in Supplementary Fig. 6. In particular, the HCP nucleation yields a higher N^*^ when *R* < 1, whereas the N^*^ becomes lower for *R* ≥ 1. Overall, the divergence between the two nucleation modes results in distinct patterns of variation in ∆G^*^ and N^*^. Consequently, this analysis justifies applying the HCP nucleation model to Zn electrodeposition, revealing that *γ* and *η* jointly govern nucleation thermodynamics and spatial uniformity.

### Experimental validation of the Zn-HCP nucleation theory

**Fig. 2:**
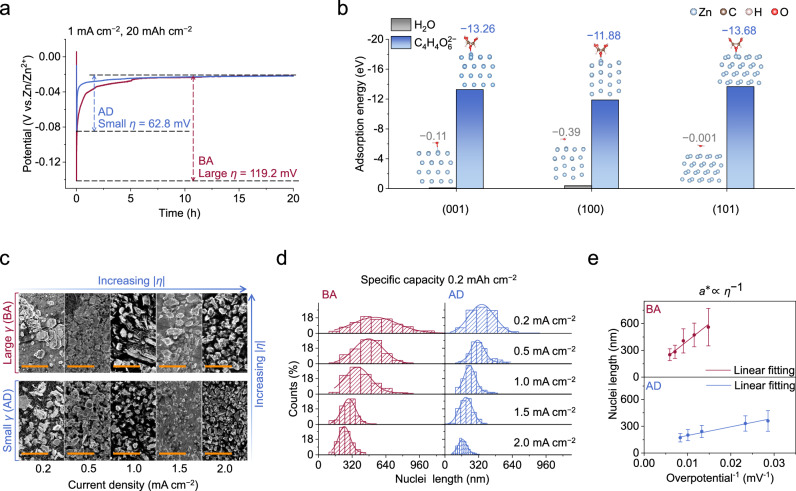
Experimental validation of the Zn-HCP nucleation theory. **a** Nucleation overpotential curve of the bare (BA, red) and additive (AD, blue) electrolytes in Zn||Zn symmetric cells. **b** Adsorption energy of tartrate ions (blue) and water molecules (gray) on Zn facets. The optimized atomic coordinates for all adsorption configurations are provided as Supplementary Data 1–3. **c** Scanning electron microscopy (SEM) images of Zn deposition morphologies under varying *η* and *γ* (scale bar: 2 μm). **d** Statistical correlation between Zn nuclei length and polarization during Zn deposition in BA (red) and AD (blue) electrolytes. Bars represent the mean values, with individual data points overlaid to show the distribution. **e** Linear fitting of *η*^−1^ versus the nuclei length. Data points represent the mean values, and error bars indicate the standard deviation (SD). The sample sizes (n, representing the number of statistically counted crystal nuclei) for BA at current densities of 0.2, 0.5, 1.0, 1.5, and 2.0 mA cm^−2^ are 204, 181, 222, 112, and 151, respectively. For AD, the corresponding sample sizes are 201, 186, 152, 130, and 156, respectively. All electrochemical tests in this figure are conducted at 25 ± 2 °C.

To validate the theoretical framework, electrolytes with tunable surface energy and overpotential are formulated by introducing ionic additives. The additive-containing and additive-free electrolytes are denoted as AD and BA, respectively. As shown in Fig. 2a, the introduction of additives reduces the Zn^2+^ deposition overpotential (*η*) from 119.2 mV (BA) to 62.8 mV (AD). Adsorption energy calculations (Fig. 2b) reveal that additive ions exhibit stronger adsorption on Zn facets (001, 100, 101) at −13.26, −11.88, and −13.68 eV, respectively. These values are significantly higher than the −0.11, −0.39, and −0.001 eV reported for water molecules, indicating a substantial reduction in interfacial energy^44^. This effect is supported by the decrease in contact angle from 97.6° to 80.6° (Supplementary Fig. 7), confirming enhanced wettability and reduced interfacial tension. Chronoamperometry (CA) measurements on Zn||Zn and Zn||Cu cells confirm instantaneous nucleation in both electrolytes (Supplementary Fig. 8)^45^. As derived from the Butler-Volmer relation (Supplementary Equation (27)), the current density and overpotential during cross-flow electrodeposition in symmetric cells satisfy Eq. (8) (derivation in Supplementary Note 3). Accordingly, under galvanostatic conditions, the overpotential *η* increases with increasing current density, a trend confirmed experimentally (Supplementary Fig. 9).

8 $$\eta=\frac{{\rm{R}}T}{\alpha {\rm{z}}{\rm{F}}}{\mathrm{ln}}\left(\frac{j}{{2j}_{0}}+\sqrt{{\left(\frac{j}{{2j}_{0}}\right)}^{2}+1}\right)$$

Subsequently, galvanostatic deposition of 0.2 mAh cm^−2^ Zn is performed on Zn foil (Supplementary Fig. 10) at current densities of 0.2, 0.5, 1.0, 1.5, and 2.0 mA cm^−2^. Scanning electron microscopy (SEM) imaging (Fig. 2c, Supplementary Figs. 11–15) reveals HCP nucleation morphology, and statistical analysis (Fig. 2d) demonstrates that increasing current density elevates the nucleation overpotential (BA: 67.57 → 169.49 mV, AD: 35.09 → 120.48 mV) while reducing the nuclei length (BA: 560.0 → 251.4 nm, AD: 359.9 → 171.8 nm). At each current density, the AD with lower *γ* consistently yields smaller nuclei lengths than BA (Supplementary Fig. 16), consistent with prior reports^20,28,46^. The linear correlation between *η*^−1^ and nuclei length further validates the predicted scaling relation (Eq. (4), Fig. 2e). Moreover, the AD exhibits smaller standard deviations (*σ*) for length distributions (*σ* = 116.2 → 47.0 nm vs. 65.9 → 209.6) and stronger (002) texture (RTC_(002)_: 22.139 vs. 17.925) (Supplementary Fig. 17). Supplementary Fig. 18 presents further characterization of the interfacial kinetics of the electrode after nucleation under the specified current density and areal capacity conditions. The AD electrolyte shows a higher current response and faster interfacial kinetics in the CA test, which are associated with a higher nucleation site density. Consequently, these results validate the Zn-HCP nucleation model and demonstrate that regulation of *γ* and *η* enables uniform Zn electrodeposition.

### Dimensionless quantification of electrodeposition kinetics

The overpotential (*η*) acts as the primary driving force for electrodeposition and determines the nucleation rate, growth kinetics, and the final deposition morphology. In contrast, the intrinsic potential of Zn at −0.76 V versus the standard hydrogen electrode (SHE) ensures that its oxidation is thermodynamically spontaneous in aqueous electrolytes. The surface energy (*γ*) defines the interfacial thermodynamic barrier and quantifies interfacial resistance. A balance between *η* and *γ* is therefore required to achieve efficient and uniform electrodeposition.

In practical applications, *η* is converted into actual electrodeposition only when the interface provides sufficient charge transfer and mass transport. This process is characterized by the transfer coefficients (*α*) in the Butler-Volmer relation (Supplementary Eq. (27)). However, interfacial resistance and parasitic reactions consume a portion of this driving force, reducing the deposition efficiency. A dimensionless descriptor, *W*_f_, is introduced in Eq. (9) to quantitatively describe the interplay between the driving force and interfacial resistance.

9 $${W}_{{\rm{f}}}=\frac{\Delta E}{I\times Z}$$

**Fig. 3:**
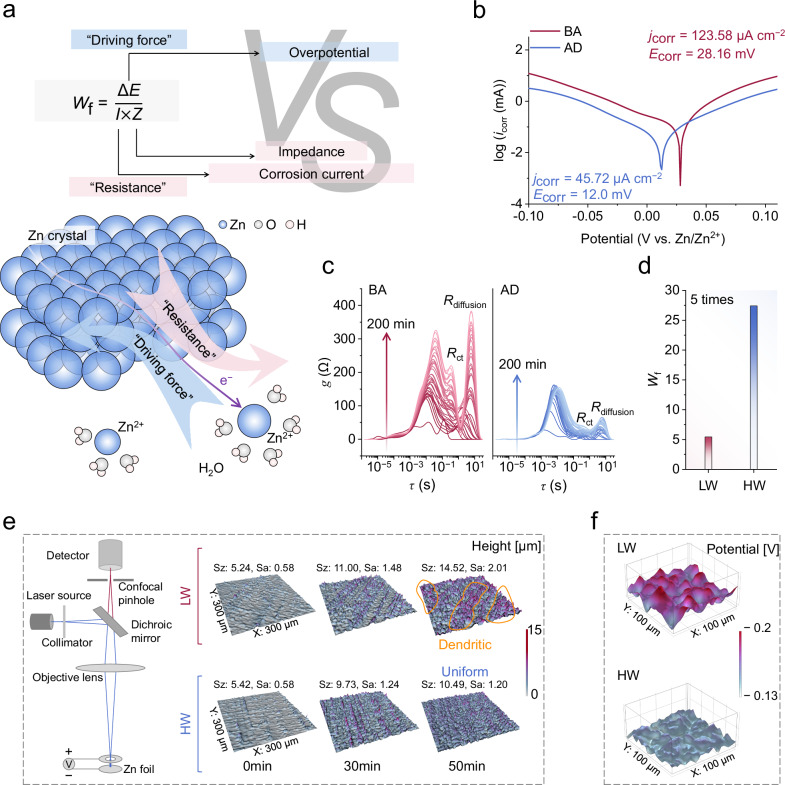
Dimensionless quantification of electrodeposition kinetics. **a** Schematic illustration of the dimensionless descriptor *W*_f_, illustrating the competitive interplay between interfacial driving force and interfacial resistance during Zn electrodeposition. **b** Tafel polarization curves of the bare (BA, red) and additive (AD, blue) electrolytes. **c** In situ distribution of relaxation times (DRT) spectra of Zn||Zn symmetric cells in BA (red) and AD (blue). **d** Calculation of *W*_f_ values for low-*W*_f_ (LW) and high-*W*_f_ (HW) electrolytes. **e** In situ electrochemical laser scanning confocal microscope (ELSCM) and corresponding Zn deposition morphologies at 0, 30, and 50 min under 10 mA cm^−2^. **f** Finite-element simulations of the surface electric-potential distribution. All electrochemical tests in this figure are conducted at 25 ± 2 °C.

Formally, *W*_f_ is defined as the ratio between the polarization overpotential (∆*E*, *η*) and the product of corrosion current (*I*, *i*_corr_) and polarization resistance (*Z*, Supplementary Eq. (32)). Because *Z* generally exceeds  *R*_ct_ (charge-transfer resistance), reflecting additional contributions from diffusion, double-layer capacitance, and resistive partitioning, it is approximated as *R*_ct_ when diffusion limitations are negligible (Supplementary Eq. (44))^47^. Therefore, *W*_f_ is calculated through Supplementary Equation (45) (derivation in Supplementary Note 3), providing a quantitative metric to evaluate the competition between the deposition driving force and interfacial resistance (Fig. 3a). A higher *W*_f_ value indicates enhanced deposition efficiency and a more stable electrode/electrolyte interface. Tafel measurements and in situ distribution of relaxation times (DRT) are employed to quantify the corrosion current and interfacial resistances (*R*_ct_ and *R*_diffusion_ resistance). Specifically, AD exhibits a significantly lower corrosion current density (45.72 μA cm^−2^) than BA (123.58 μA cm^−2^) (Fig. 3b). In situ DRT analysis after 200 min at open circuit reveals a pronounced increase in both *R*_ct_ and *R*_diffusion_ for BA, confirming enhanced interfacial anti-corrosion in AD (Fig. 3c)^48^. Based on these parameters, *W*_f_ values are calculated for electrolytes with varying additive contents (Supplementary Fig. 19). The highest-*W*_f_ electrolyte is denoted as HW, whereas the lowest (bare) counterpart is identified as LW. *W*_f_ for HW is 5 times higher than for LW (Fig. 3d).

To further confirm the practical relevance of *W*_f_, in situ electrochemical laser scanning confocal microscope (ELSCM) are performed in a custom-designed cell to monitor Zn deposition in HW and LW electrolytes at 10 mA cm^−2^ for 50 min (Fig. 3e). HW exhibits a smoother and more uniform deposition. The altitude intercept (Sz) and surface roughness (Sa) increase only to 10.49 μm and 1.20 μm, respectively, while values of 14.52 μm and 2.01 μm are observed for LW. Abbott-Firestone bearing area curve analysis and power spectral density (PSD) analysis (Supplementary Fig. 20) reveal that the HW yields a more uniform electrode interface height and a more uniform spatial distribution of deposited features during Zn deposition (Supplementary Table 3). Non-uniform interfacial potentials in LW concentrate local current densities, promoting tip growth and dendrite formation. Finite-element simulations reveal that, at a current density of 5 mA cm^−2^, the HW maintains a more uniform surface electric-potential distribution (variation <0.16 V) than LW (Fig. 3f), thereby mitigating tip-induced parasitic reactions and inhibiting dendrite propagation^49^. Furthermore, in situ monitoring of dendrite growth (Supplementary Fig. 21) during Zn deposition at a current density of 5 mA cm^−2^, with interface reconstruction every 10 s, reveals the detailed growth process. In the LW, dendrite height increases substantially from 18.89 μm at 0 s to 75.44 μm at 60 s. This sharp increase indicates a high risk of separator piercing. In contrast, the dendrite height in HW rises only modestly from 18.26 μm at 0 s to 24.93 μm at 60 s, resulting in a more uniform and stable deposition interface. Collectively, these results verify that *W*_f_ quantitatively reflects the interplay between the deposition driving force and interfacial resistance, serving as a reliable descriptor for guiding practical electrodeposition processes.

### Interfacial behavior and electrochemical performance of LW and HW electrolytes

**Fig. 4:**
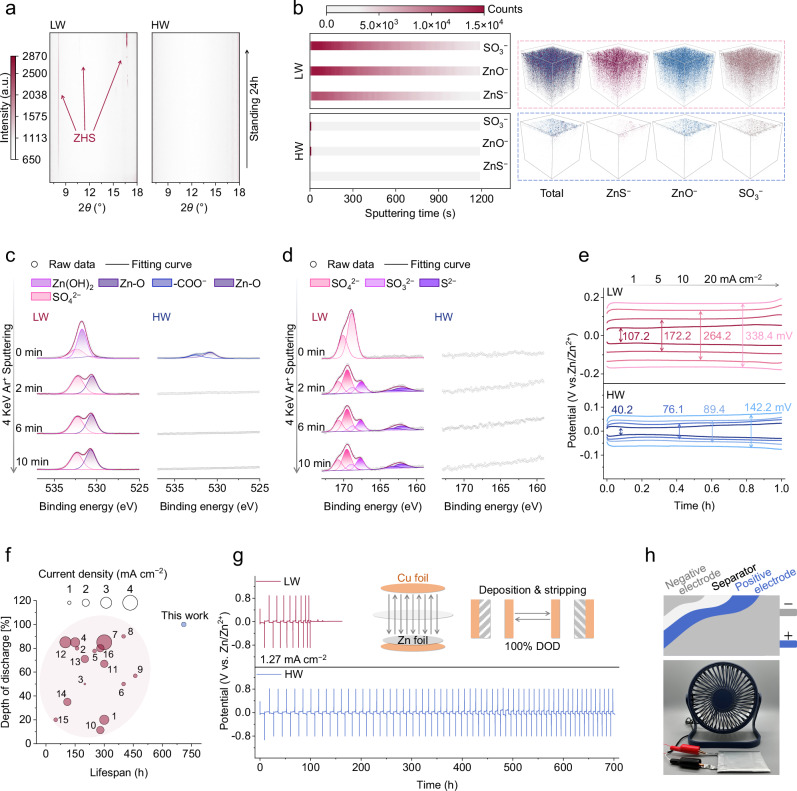
Interfacial behavior and electrochemical performance of LW and HW electrolytes. **a** In situ X-ray diffraction (XRD) patterns of Zn metal after immersion in low-*W*_f_ (LW) and high-*W*_f_ (HW) electrolytes. **b** Interphase composition depth distribution curves and corresponding spatial distributions of Zn electrodes after soaking in different electrolytes for 4 h. Depth-profiling X-ray photoelectron spectroscopy (XPS) spectra of (**c**) O 1 *s* and (**d**) S 2*p* of Zn metal following 5 days of immersion in LW and HW electrolytes. **e** Voltage curves of Zn||Zn symmetrical cells with LW (red) and HW (blue) at current densities of 1, 5, 10, and 20 mA cm^−2^. The Zn foil with a thickness of 100 μm is employed. **f** Statistical comparison of Depth of Discharge (DOD) testing, conducted at a low current density of <5 mA cm^−2^ with data from published work. The source of the literature data shown in this figure is provided in Supplementary Information, Table 5. **g** The voltage curves of LW (red) and HW (blue) electrolytes during DOD testing, demonstrating the testing protocol, with the Zn foil used having a thickness of 2 μm. **h** A fan driven by a ZAB pouch cell (single piece, 4 cm × 5 cm, N/P ratio 2.62), and its schematic diagram. All electrochemical tests in this figure are conducted at 25 ± 2 °C.

Interfacial chemical barriers fundamentally determine the stability of both the electrolyte and the electrode interface. Electrolytes with higher *W*_f_ exhibit lower charge-transfer resistance and reduce corrosion current. These characteristics lead to improved interfacial stability and a prolonged electrode lifespan during both cycling and storage. Such electrolytes also demonstrate higher bulk ionic conductivity (Supplementary Fig. 22) and larger diffusion coefficients (Supplementary Fig. 23). Consequently, variations in electrolyte composition modulate the interfacial chemistry of the Zn metal and necessitate an investigation into electrolytes with different *W*_f_ values at the negative electrode. In situ X-ray diffraction (XRD) of Zn foils immersed in the LW and HW electrolytes reveals characteristic reflections at 8.3^°^ and 16.4^°^. These peaks correspond to the formation of zinc hydroxide sulfates (ZHS) in the LW electrolyte and intensify over time^50^, indicating severe side reactions (Fig. 4a). In contrast, no such reflections are detected in the HW electrolyte. Ex situ XRD analysis performed after 20 days confirms these findings, showing that strong ZHS reflections persist for LW but are absent for HW (Supplementary Fig. 24), demonstrating the highly effective anti-corrosion properties of the HW electrolyte.

Time-of-flight secondary ion mass spectrometry (ToF-SIMS) is employed to reveal the chemical composition and spatial distribution of corrosion layers on Zn foils soaked in HW and LW electrolytes (Fig. 4b). The analyzed ion fragments include SO_3_^−^, ZnO^−^, and ZnS^−^. For Zn surfaces soaked in the HW electrolyte, signal intensities remain confined to the first 30 s of sputtering, indicating negligible surface corrosion. In contrast, the LW electrolyte exhibits strong signals throughout the entire 1200 s sputtering process, signifying the formation of a substantially thicker ZHS corrosion layer and thus highlighting the enhanced interfacial stability of HW.

To further elucidate the underlying chemical states, depth-profiling X-ray photoelectron spectroscopy (XPS) is conducted after 5 days of immersion. In the LW, after 10 min of 4 keV Ar^+^ sputtering, the O 1*s* spectrum shows pronounced peaks at 532.2 and 530.5 eV, and the S 2*p* region displays sulfate-related features, consistent with the formation of a compact ZHS layer (Fig. 4c, d). By contrast, no sulfate-related signals are detected for the HW electrolyte under the same sputtering conditions. Only features attributable to an adsorbed tartrate layer are observed without evidence of ZHS formation. Sulfite and sulfide signals likely result from Ar^+^ bombardment, where preferential sputtering induces oxygen depletion and concurrent electron bombardment and localized heating promote S-O bond reduction, partially converting the original sulfate (SO_4_^2−^) into lower-valence sulfur species (SO_3_^2−^ and S^2−^). This conclusion is further supported by the Zn 2*p* spectra, which show metallic Zn peaks in the HW after 2 min of sputtering (about 20 nm). In contrast, the LW displays only Zn-O bonds throughout the depth-profile (Supplementary Fig. 25)^51^.

Furthermore, the HW electrolyte shifts the hydrogen-evolution onset potential to −142 mV vs. Zn/Zn^2+^ compared with −97 mV for LW (Supplementary Fig. 26). To further validate the long-term pH stability, in situ local pH measurements (Supplementary Fig. 27) are performed during Zn deposition/stripping at 1 mA cm^−2^ for 6 h. The HW showed more stable pH fluctuations, with an average of 4.84, compared to 5.12 for the LW, confirming its sustained pH stability. At a current density of 0.75 mA cm^−2^, the HW electrolyte positively shifts the Zn^2+^ reduction potential in Zn||Zn cells (−0.0642 V for HW vs. − 0.0701 V for LW, Supplementary Fig. 28). The formation of these stable chemical interfaces is attributed to the adsorption of additive molecules on the electrode surface (Supplementary Fig. 29). Collectively, a high *W*_f_ suppresses parasitic ZHS, mitigates hydrogen evolution, and facilitates charge transfer, thereby enabling a high *W*_f_, which is essential for the long-term reversibility of Zn electrodes.

A higher *W*_f_ corresponds to more efficient Zn electrodeposition as reflected in the polarization behavior. Under galvanostatic conditions, LW exhibits substantial and unstable overpotentials ranging from 107.2 to 338.4 mV over 1–20 mA cm^−2^. In contrast, HW maintains much lower and steadier values between 40.2 and 142.2 mV (Fig. 4e). Rate tests further corroborate this trend (Supplementary Fig. 30). This stability translates into markedly prolonged lifetimes of 4,744 h and 4,335 h at 1 and 5 mA cm^−2^ (unit area capacity 1 mAh cm^−2^), over ten times longer than those of LW (392 and 155 h) (Supplementary Fig. 31). To further investigate the evolution of interfacial chemistry and plating/stripping kinetics during prolonged working, cells after 100 h and 200 h plating/stripping are characterized using EIS, XRD, and XPS (Supplementary Fig. 32). LW leads to the formation of ZHS byproducts during this process. In contrast, HW maintains a more stable plating/stripping interface. HW also sustains 99.4% Coulombic efficiency (CE) over 1000 cycles, which stands in contrast to LW. The latter achieves only 98.8% CE and exhibits fewer than 350 cycles of durability (Supplementary Fig. 33). Depth of discharge (DOD) analyses further confirm that LW suffers severe polarization beyond 82 % DOD. In contrast, HW preserves stable interfacial kinetics (Supplementary Fig. 34). When benchmarked against previously reported DOD performance (Fig. 4f), the present results substantiate the markedly enhanced Zn plating and stripping reversibility enabled by the HW electrolyte. To evaluate cell performance under extreme conditions, a 100% depth of discharge (DOD) test is conducted at a low current density (<5 mA cm^−2^), during which Zn is fully plated and stripped on the current collector. The voltage curves are shown in Fig. 4g. Throughout the test, the HW cell exhibits robust stability for more than 700 h, while the LW cell short-circuits after only 7 Zn plating/stripping cycles.

The advantages of HW extend to full-cell configurations. When coupled with nanorod sodium vanadate (SEM images, Supplementary Fig. 35) positive electrodes, HW-based Zn||NaV_3_O_8_ cells deliver 258.35 mAh g^−1^ at 10 A g^−1^, slightly higher than 249.61 mAh g^−1^ for LW (Supplementary Fig. 36). In terms of cycling stability, HW retains 61.7% of its initial capacity after 7660 cycles. In contrast, LW maintains only 20.4% after 5200 cycles and eventually short-circuits. Differential capacity (dQ/dV) analysis corroborates that HW fosters more favorable interfacial kinetics (Supplementary Fig. 37). Practical pouch-cell tests (N/P ratio 2.62) also demonstrate the same trend. The HW cell, which utilizes a NaV_3_O_8_ positive electrode (single piece, 4 × 5 cm^2^) and 10 μm Zn foil (Fig. 4h), delivers 45 mAh with 97% retention after 200 cycles at 6.44 mA cm^−2^. By comparison, the LW cell exhibits a rapid capacity decay to 38.6% after 190 cycles and fails due to dendritic short-circuiting (Supplementary Fig. 38). These results demonstrate that high-*W*_f_ electrolytes substantially enhance Zn electrode reversibility and full-cell durability. This enhancement is achieved by enabling effective interfacial stabilization and uniform Zn^2+^ deposition kinetics.

### Analysis and verification of the dimensionless descriptor

**Fig. 5:**
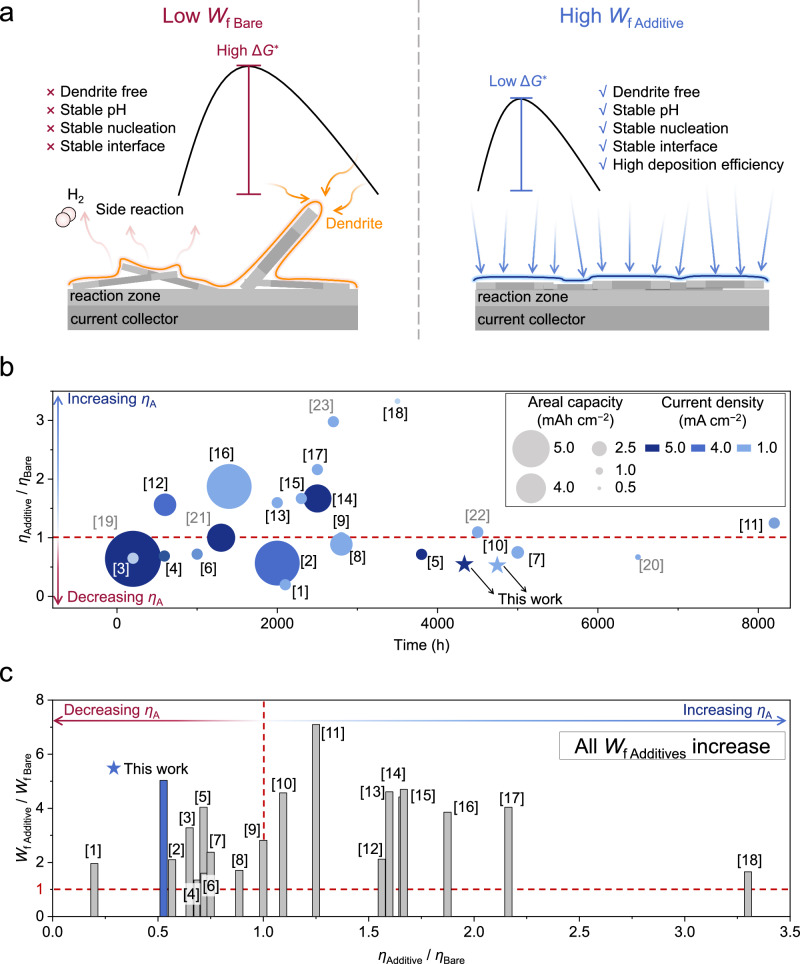
Analysis and verification of the dimensionless descriptor. **a** Schematic comparison of Zn deposition morphologies corresponding to different *W*_f_ values. **b** Statistical correlations among lifespan, current density, areal capacity, and polarization magnitude for various additives. **c** Statistics of the dimensionless descriptor *W*_f_ reported in related works. The source of the literature data shown in this figure is provided in Supplementary Information, Table 6.

The parameterization of interfacial behavior with the dimensionless descriptor *W*_f_ allows for the systematic delineation of electrodeposition regimes (Fig. 5a). At low *W*_f_ values, the denominator term, which incorporates corrosion currents and charge-transfer resistance, dominates the expression. This dominance suppresses the driving force for electrodeposition and facilitates parasitic reactions. Such an imbalance leads to unstable interfaces, dendritic growth, and local pH fluctuations. Conversely, high *W*_f_ indicates the dominance of the driving-force term, corresponding to efficient electrodeposition with minimized corrosion and interfacial resistance. Under these conditions, Zn deposition proceeds with uniform nucleation, stable interfacial chemistry, dendrite-free morphology, and high efficiency.

Numerous additives for ZABs are documented in Supplementary Table 6. Figure 5b summarizes the relationship among overpotential, areal capacity, current density, and lifespan in Zn||Zn cells. The ratio *η*_additive_*/η*_bare_ reflects the additive-induced change in overpotential, where values below unity indicate a reduced *η* and values above unity indicate an enhanced *η*. However, *η* alone does not reliably predict deposition stability because the interfacial resistance, which is particularly related to surface energy, is often neglected. Therefore, a comprehensive evaluation requires simultaneous consideration of both *η* and interfacial resistance.

In this context, the dimensionless descriptor *W*_f_ reconciles the interplay between *η* and interfacial resistance, enabling a quantitative evaluation of electrolyte performance. To evaluate its general applicability, *W*_f_ values are computed for reported electrolytes and normalized to their bare counterparts (*W*_f,Additive_ / *W*_f,Bare_). This normalization eliminates variations arising from electrolyte concentration or cell differences and provides a consistent metric. Notably, all additives yield a higher *W*_f_ than the bare electrolyte (Fig. 5c), validating *W*_f_ as a generally applicable descriptor of electrolyte performance. Such an improvement is reflected in elevated *W*_f_ values that result from additive adsorption at the negative electrode surface, an altered Zn^2+^ solvation structure, an improved electric double layer, and optimized interfacial reaction kinetics.

The introduction of *W*_f_ unifies the deposition driving force and interfacial resistance into a single experimentally accessible metric. This framework enables a quantitative comparison of diverse electrolytes through macroscopic electrochemical data and offers practical guidance for the rational design of electrolytes and the Zn electrode interface. Furthermore, because both the numerator (∆*E*) and denominator (*I* × *Z*) of *W*_f_ are derived from quantifiable electrochemical parameters, their integration with machine-learning approaches shows strong potential for high-throughput electrolyte screening and precise compositional optimization.

## Discussion

In summary, this work advances the understanding of CNT by integrating an HCP model to elucidate the coupled roles of overpotential and surface energy in Zn^2+^ electrodeposition. A dimensionless descriptor, *W*_f_, is defined to quantitatively unify the electrodeposition driving force and interfacial resistance into a single experimentally accessible metric. The validity of this framework is corroborated by theoretical modelling and experimental observations, providing a unified thermodynamic-kinetic basis for predicting nucleation behaviour and guiding strategies to mitigate dendritic Zn growth. Furthermore, the application of the *W*_f_ principle to electrolyte design, corroborated by in situ evolution analysis, microstructural characterization, and first-principles simulations, shows that a high *W*_f_ establishes a highly stable negative electrode/electrolyte interface, thereby promoting denser and more uniform Zn deposition. As a result, the optimized high-*W*_f_ electrolyte achieves prolonged Zn||Zn lifespan for 4740 h and Zn||NaV_3_O_8_ cell cycling for 7660 cycles. Overall, *W*_f_ serves as a robust and generalizable criterion for electrolyte design and guides the development of dendrite-free, corrosion-resistant, and long-lasting Zn metal electrodes.

## Methods

### Materials

Zinc sulfate heptahydrate (ZnSO_4_·7H_2_O), ammonium tartrate (AR, 99%), V_2_O_5_ (99%), and N-methyl-2-pyrrolidone (NMP) (99.9%) are purchased from Shanghai Aladdin Biochemical Technology Co, Ltd. Carbon black (Super P) is purchased from TIMCAL. Poly(vinylidene fluoride) (PVDF) (Solef 5130) is purchased from Solvay. NaCl (AR, 99.5%), and ethanol absolute (99.9%) are purchased from Sinopharm Chemical Reagent Co, Ltd. The copper foils used in this study had a thickness of 10 μm and an Sq of approximately 600 nm. A commercial 100-mesh titanium (Ti) mesh is employed as the current collector, as-received, without any further chemical or physical treatments. The Ti mesh possesses an average pore size of approximately 140–150 μm and an areal density of 26.40 mg cm⁻². The GF/A (Whatman, Cat. No. 1820-47) and GF/D (Whatman, Cat. No. 1823-047) glass fibers, featuring a porosity of ~90% and average pore sizes of 1.6 and 2.7 μm, respectively, are employed as separators. These membranes have thicknesses of 260 μm (GF/A) and 675 μm (GF/D) and are cut into circular discs with a diameter of 16 mm. Commercial Zn foils (99.9%) with various thicknesses (100, 50, 20, and 10 μm) and a root-mean-square roughness (Sq) of ~500 nm are employed. Before use, the foils are thoroughly cleaned with ethanol and deionized water, then cut into 12 mm circular discs. The specific thickness of the Zn electrode for each test is provided in the corresponding figure caption.

### Preparation of electrolytes and electrodes

The electrolyte solutions are prepared by dissolving ZnSO_4_·7H_2_O (ZS) and ammonium tartrate (AT) at various concentrations. The electrolyte is labeled as HW (ammonium tartrate concentration is 15 mM, with ZS concentration of 1 M). The electrolyte without AT is labeled as BA (LW). For ex situ characterizations, the cycled cells are disassembled in ambient air. The harvested electrodes are immediately rinsed several times with deionized water to remove surface residuals, then dried. Both the sampling and transport of these electrode materials are performed under atmospheric conditions at a controlled temperature of 25 ± 2 °C. No inert gas protection or specialized environmental chambers are employed during the sample preparation and transfer process for the various technical measurements.

### Preparation of NaV_3_O_8_

Generally, 2.0 g of V_2_O_5_ is added to 30 mL of 2 M NaCl solution under constant stirring. After continuous stirring for 96 h at 25 ± 2 °C, the suspension exhibits a distinct color change from yellow to brown, indicating the formation of intermediate vanadium species. The products are collected and washed via suction filtration three times with deionized water and once with ethanol. Subsequently, the obtained powders are dried in a vacuum oven at 80 °C for 12 h to yield the final NaV_3_O_8_ powders.

### Electrochemistry test

All electrochemical performance measurements and cycling tests are performed in a temperature-stabilized laboratory environment, without the use of a climatic chamber. The ambient temperature is maintained at 25 ± 2 °C throughout the experiments. CR2032 coin cells are assembled using stainless steel cases, thick spacers (1.0 mm), and spring washers to maintain consistent internal pressure, with 80 μL of electrolyte added to the GF/D separator. Zn foil serves as the working electrode, and copper foil as the counter electrode, when assembling the half-cell. A symmetric Zn||Zn cell is assembled using Zn foil as both working and counter electrodes. For the 100% DOD test based on Cu symmetric electrodes, the Zn foil is placed between two Cu foils to achieve complete plating and stripping. Sodium vanadate (NaV_3_O_8_) is used as the positive electrode in a full-cell with a Zn electrode. The NaV_3_O_8_ positive electrode slurry is prepared by mixing NaV_3_O_8_, carbon black, and PVDF (7:2:1 mass ratio) in NMP solvent. The resulting slurry is uniformly brushed onto the 100-mesh Ti mesh current collector, followed by vacuum-drying at 80 °C for 12 h. The pouch cell is assembled in a Zn||NaV_3_O_8_ configuration, using a 10 μm-thick Zn foil and a NaV_3_O_8_-loaded Ti mesh (4 × 5 cm^2^, loading ~0.25 g) as the electrodes, yielding an N/P ratio of 2.62. A Whatman GF/A membrane served as the separator, into which the electrolyte is introduced via syringe injection at a dosage of 60 μL cm^−2^. Subsequently, the pouches are vacuum-sealed without a further gas release step. During electrochemical cycling, no external pressure is applied. The cell relies solely on the intrinsic pressure generated by the vacuum-sealing process. Electrochemical tests (EIS, linear polarization, CV) are conducted on a CHI660E workstation, and galvanostatic charge/discharge cycling is performed using the Neware battery testing system. Potentiostatic EIS (200 kHz-100 mHz, 10 mV amplitude, 10 points/decade) is performed after 10 min OCV stabilization at 25 ± 2 °C.

### Materials characterization

The morphology is analyzed using a scanning electron microscope (Zeiss Gemini SEM 560 and Hitachi SU-8600). X-ray diffraction patterns are collected using a Bruker D8 diffractometer operating at 40 kV and 25 mA with Cu kα radiation (*λ* = 0.15405 nm). Roughness testing and data analysis of the Zn deposition surface are performed using a VT6000 confocal microscope (Shenzhen CHOTEST Co., Ltd). X-ray photoelectron spectroscopy (XPS) and depth-profiling are conducted using a Thermo Scientific ESCALAB QXi spectrometer equipped with a monochromatic Al Kα X-ray source (hν = 1486.6 eV). The depth-profiling is performed via Ar^+^ ion etching at an acceleration voltage of 4 keV. Survey spectra are recorded at a pass energy of 100 eV, while high-resolution scans are collected at 20 eV. All spectra are energy-calibrated by referencing the adventitious C 1*s* peak to 284.8 eV. Data processing, including Shirley-type background subtraction and peak fitting using Gaussian-Lorentzian (G/L) functions, is performed using the Thermo Scientific Avantage software. The component distribution on the Zn electrode surface is further characterized by Time-of-flight secondary ion mass spectrometry (PHI nano TOF3), equipped with a 2 keV Ar^+^ sputter gun. The contact angle measuring instrument (SDC-100) is used to measure the hydrophilicity of Zn foils soaked in different electrolytes.

### Computational methods

All the Density Functional Theory (DFT) calculations are performed by CP2K with the mixed Gaussian and plane wave (GPW) approach. A double zeta basis set is used for all atom types to treat valence electrons, while pseudopotentials of the Goedecker-Teter-Hutter type are used for core electrons. For the auxiliary plane-wave (PW) basis, the PW energy cutoff is set to 400 Ry and the reference grid cutoff is set to 55 Ry. The exchange-correlation functional used is the Perdew, Burke, Ernzerhof (PBE) functional. Van der Waals forces are approximated using the correction of Grimme. All geometry relaxations and single point energy calculations are performed in corresponding quantity of charge utilizing periodic boundary conditions in XY. The adsorption energies are defined as follows:

$${E}_{{\rm{ads}}}={E}_{{\rm{adsorbate}}+{\rm{substrate}}}-{E}_{{\rm{adsorbate}}}-{E}_{{\rm{substrate}}}$$

where, *E*_total_ is the total energy of the adsorption system, *E*_surface_ and *E*_molecule_ represent the energies of the isolated surface and gas-phase molecule, respectively. A negative adsorption energy indicates that the molecular adsorption is thermodynamically stable. All calculations are performed using the PBE functional with an appropriate k-point grid density to ensure a convergence criterion of 10^−6^ eV.

The simulations in COMSOL Multiphysics 6.2 are divided into two distinct parts: a steady-state calculation for the 3D electric-field distribution and a transient simulation for the morphological evolution of metal electrodes (Li and Zn) during deposition. The model geometry is constructed from experimentally measured dimensions. For the transient morphological evolution, ion transport is described by the Nernst-Planck equations coupled with mass and charge conservation. Interfacial charge transfer is implemented using Butler-Volmer kinetics, with the Faradaic flux coupled to species transport at the electrode-electrolyte interface. A three-dimensional (3D) transient current distribution model is coupled to a geometric-deformation module to capture the dynamic interplay between electrochemical deposition and surface morphology, employing an implicit BDF scheme with adaptive time stepping. Conversely, the 3D electric-field distribution is calculated under steady-state conditions using COMSOL’s Electric Field interface. In both models, the stripping electrode potential is fixed at 0 V, while the depositing electrode is treated as a conductive boundary with a prescribed equilibrium potential or an imposed normal surface current density. Mesh refinement is applied near interfaces, and mesh independence is confirmed by observing that key observables varied by less than 2%. Material properties are taken from the COMSOL library, while the ionic diffusion coefficients (Supplementary Fig. 23) and charge transfer coefficients (Supplementary Fig. 9) are adopted from experimental measurements.

In the DRT analysis, the peaks with relaxation time (*τ*) of ~0.01 s, 0.1 s, and 7 s are attributed to the adsorption and desorption of Zn^2+^ (*R*_ad_), interface mass transfer impedance (*R*_ct_), and diffusion of Zn^2+^ on the Zn electrode (*R*_diffusion_), respectively. *R*_ct_ and *R*_diffusion_ in the Zn electrode showed smaller *τ* values, indicating faster interface reaction kinetics.

## Supplementary information

Supplementary Information

Description of Additional Supplementary Files

Supplementary Data 1–3

Transparent Peer Review file

## Source data

Source Data

## Data Availability

Source data are provided with this paper.
