# Supplementary material for: Revisiting Zn-specific nucleation via a dimensionless factor to quantify interfacial electrochemistry of aqueous batteries
Source: Nat Commun. 2026 Jun 2;17:7067. doi: 10.1038/s41467-026-73953-w (PMC13392247; doi:10.1038/s41467-026-73953-w)
Supplement: Supplementary file 1 — Supplementary Information [file 41467_2026_73953_MOESM1_ESM.pdf]

Supplementary Information

Revisiting Zn-specific nucleation via a dimensionless factor to quantify interfacial electrochemistry of aqueous batteries

Zeyu Wang[1], Wanhai Zhou[1], Gaoyang Li[1], Zhuo Yang,[1] Zefang Yang[1], Yuhang Liu[1], Tengsheng Zhang[1], Hongrun Jin[1], Shixiang Ding[1], Junwei Zhang[1], Xia Wang[1], Fanxing Bu[1], Min Wang[1], Jingwen Zhao[2], Zaiwang Zhao[3], Dongyuan Zhao[1, 3], Dongliang Chao[1]*

[1] Laboratory of Advanced Materials, Aqueous Battery Center, Shanghai Key Laboratory of Molecular Catalysis and Innovative Materials, Collaborative Innovation Center of Chemistry for Energy Materials, Shanghai Wusong Laboratory of Materials Science, State Key Laboratory of Porous Materials for Separation and Conversion, College of Smart Materials and Future Energy, Fudan University, Shanghai 200433, China

[2] Qingdao Industrial Energy Storage Research Institute, Qingdao Institute of Bioenergy and Bioprocess Technology, Chinese Academy of Sciences, Qingdao 266101, China

[3] College of Energy Materials and Chemistry, College of Chemistry and Chemical Engineering, Inner Mongolia University, Hohhot 010070, China

* Corresponding to chaod@fudan.edu.cn (D.C.)

Supplementary Notes

Supplementary Note 1. Thermodynamic derivation of spherical and HCP nucleation models.

Supplementary Equation (1) is the main equation of classical nucleation theory (CNT), which is used to explain and predict the formation of small particles (nucleation) of a new phase from a homogeneous phase during phase transitions, such as liquid solidification, gas condensation, or liquid evaporation. In the equation, ΔG_{v} represents the bulk free energy, and ΔG_{s} represents the surface free energy.

$$\Delta G = -\Delta G_{\mathrm{v}} + \Delta G_{\mathrm{s}} \tag{1}$$

The bulk free energy and surface free energy can be expressed by **Supplementary Equation (2)** and **Supplementary Equation (3)**.

$$\Delta G_{\mathrm{v}} = V\Delta g_{\mathrm{v}} \tag{2}$$

$$\Delta G_{\mathrm{s}} = S\gamma \tag{3}$$

$$\Delta g_{\mathrm{v}} = \frac{\mathrm{zF}\eta}{V_{\mathrm{m}}} \tag{4}$$

$$\Delta G = -V\frac{\mathrm{zF}\eta}{V_{\mathrm{m}}} + S\gamma \tag{5}$$

where V is the volume of the model, Δg_{v} is the free energy change per unit volume (typically negative), S is the surface area of the model, and γ is the interfacial tension between the new phase and the surrounding environment. z is the charge number, F is the Faraday constant, η is the overpotential, and V_{m} is the molar volume. Based on the spherical nucleus model (with radius r) and the hexagonal nucleus model (with side length a and height h), different expressions for ΔG can be obtained.

$$\Delta G_{\mathrm{spherical}} = -\frac{4}{3}\pi r^3 \Delta g_{\mathrm{v}} + 4\pi r^2 \gamma \tag{6}$$

$$\Delta G_{\mathrm{hexagonal}} = 3\sqrt{3}a^2\left(\gamma - \frac{h\Delta g_{\mathrm{v}}}{2}\right) + 6ah\gamma \tag{7}$$

By taking the partial derivative of the model's volume-dependent parameters and setting it to zero, as shown in **Supplementary Equations (8)** and **(9)**, the critical radius of the model in the Gibbs free energy criterion can be obtained. For the hexagonal model, the height is assumed to remain approximately constant. The results are given in **Supplementary Equations (10)** and **(11)**.

$$\frac{\partial \Delta G_{\mathrm{spherical}}}{\partial r} = 0\ , \ \frac{\partial \Delta G_{\mathrm{hexagonal}}}{\partial a} = 0 \qquad (8)\&(9)$$

$$r^* = \frac{2\gamma}{\Delta g_{\mathrm{v}}} = \frac{2V_{\mathrm{m}}}{\mathrm{zF}}\left(\frac{\gamma}{\eta}\right) \tag{10}$$

$$a^* = \frac{2\gamma}{\sqrt{3}}\left(\frac{1}{\Delta g_{\mathrm{v}} - \frac{2\gamma}{h}}\right) \tag{11}$$

where r^* and a^* represent the critical nucleation radius for the spherical nucleus and hexagonal nucleus, respectively. When the deposition overpotential dominates, **Supplementary Equation (11)** can be simplified to **Supplementary Equation (12)**.

$$a^* = \frac{2\gamma}{\sqrt{3}}\left(\frac{1}{\Delta g_{\mathrm{v}}}\right) = \frac{2V_{\mathrm{m}}}{\sqrt{3}\mathrm{zF}}\left(\frac{\gamma}{\eta}\right) \tag{12}$$

Substituting them into the original Gibbs free energy expressions yields **Supplementary Equations (13)** and **(14)**.

$$\Delta G^*_{\mathrm{spherical}} = \frac{16\pi\gamma^3}{3}\left(\frac{1}{\Delta g_{\mathrm{v}}}\right)^2 = \frac{16\pi V_{\mathrm{m}}^2}{3(\mathrm{zF})^2}\left(\frac{\gamma^3}{\eta^2}\right) \tag{13}$$

$$\Delta G^*_{\mathrm{hexagonal}} = 2\sqrt{3}h\gamma^2\left(\frac{1}{\Delta g_{\mathrm{v}}}\right) = \frac{2\sqrt{3}hV_{\mathrm{m}}}{\mathrm{zF}}\left(\frac{\gamma^2}{\eta}\right) \tag{14}$$

The relationship between the nucleation rate and the critical free energy can be approximately expressed to some extent, as shown in **Supplementary Equation (15)**.

$$J = \mathrm{A} * \exp\left(-\frac{\Delta G^*}{\mathrm{k_B}T}\right) \tag{15}$$

where $\mathrm{k_B}$ is the Boltzmann constant, and T is the temperature (in Kelvin). Therefore, the critical nucleus density N^* can be obtained, as shown in **Supplementary Equations (16)** and **(17)**.

$$N^*_{\mathrm{spherical}} \propto \exp\left[-\frac{16\pi V_{\mathrm{m}}^2}{3(\mathrm{zF})^2\mathrm{k_B}T}\left(\frac{\gamma^3}{\eta^2}\right)\right] \tag{16}$$

$$N^*_{\mathrm{hexagonal}} \propto \exp\left[-\frac{2\sqrt{3}hV_{\mathrm{m}}}{\mathrm{zFk_B}T}\left(\frac{\gamma^2}{\eta}\right)\right] \tag{17}$$

Supplementary Note 2. Evolution of free energy and critical nuclei density in spherical and HCP models.

From the Gibbs free energy (ΔG^*) of nucleation, it can be derived that the variation rates of $A = \frac{\gamma^2}{\eta}$ and $B = \frac{\gamma^3}{\eta^2}$ govern the evolution of the critical nuclei density (N^*). Therefore, a ratio between the two is defined as R (**Supplementary Equation (18)**).

$$R(\gamma,\eta) = \frac{B}{A} = \frac{\gamma}{\eta} \tag{18}$$

Based on the relative variation rates, the relative variation rates of A, B, and R are calculated and compared (**Supplementary Equation (19)**).

$$\frac{\mathrm{d}A}{A} = 2\frac{\partial\gamma}{\gamma} - \frac{\partial\eta}{\eta},\ \frac{\mathrm{d}B}{B} = 3\frac{\partial\gamma}{\gamma} - 2\frac{\partial\eta}{\eta},\ \frac{\mathrm{d}R}{R} = \frac{\partial\gamma}{\gamma} - \frac{\partial\eta}{\eta} \tag{19}$$

$$\frac{\mathrm{d}A}{A} - \frac{\mathrm{d}B}{B} = 2\frac{\partial\gamma}{\gamma} - \frac{\partial\eta}{\eta} - \left(3\frac{\partial\gamma}{\gamma} - 2\frac{\partial\eta}{\eta}\right) = \frac{\partial\eta}{\eta} - \frac{\partial\gamma}{\gamma} = -\frac{\mathrm{d}R}{R} \tag{20}$$

According to the above equation (**Supplementary Equation (20)**), it is evident that when $\frac{\mathrm{d}R}{R} > 0$, *i.e.*, when R increases ($\frac{\mathrm{d}\gamma}{\gamma} > \frac{\mathrm{d}\eta}{\eta}$), $\frac{\mathrm{d}A}{A} < \frac{\mathrm{d}B}{B}$, indicating that the relative variation rate of B is greater than that of A. In contrast, when $\frac{\mathrm{d}R}{R}$<0, *i.e.*, when R decreases ($\frac{\mathrm{d}\gamma}{\gamma} < \frac{\mathrm{d}\eta}{\eta}$) , the opposite trend occurs. This implies that the direction of R variation determines which parameter exhibits a faster relative change.

From the perspective of the absolute variation rates, that is, the magnitude of the gradients, the gradient vectors of A and B need to be calculated. Assuming that γ and η are independent variables, the following expressions can be obtained (**Supplementary Equations (21)** and (**S22)**).

$$\nabla A(\gamma,\eta) = \left(\frac{\partial A}{\partial\gamma},\frac{\partial A}{\partial\eta}\right) = \left(\frac{2\gamma}{\eta},-\frac{\gamma^2}{\eta^2}\right) \tag{21}$$

$$\nabla B(\gamma,\eta) = \left(\frac{\partial B}{\partial\gamma},\frac{\partial B}{\partial\eta}\right) = \left(\frac{3\gamma^2}{\eta^2},-\frac{2\gamma^3}{\eta^3}\right) \tag{22}$$

The Euclidean norm, the magnitude of the gradient, is calculated as follows (**Supplementary Equations (23)** and **(24)**).

$$|\nabla A| = \sqrt{\left(\frac{2\gamma}{\eta}\right)^2 + \left(-\frac{\gamma^2}{\eta^2}\right)^2} = \frac{\gamma}{\eta^2}\sqrt{4\eta^2 + \gamma^2} \tag{23}$$

$$|\nabla B| = \sqrt{\left(\frac{3\gamma^2}{\eta^2}\right)^2 + \left(-\frac{2\gamma^3}{\eta^3}\right)^2} = \frac{\gamma^2}{\eta^3}\sqrt{9\eta^2 + 4\gamma^2} \tag{24}$$

Taking the ratio of the above expressions and substituting R yields the following expression (**Supplementary Equation (25)**).

$$\frac{|\nabla A|}{|\nabla B|} = \frac{\frac{\gamma}{\eta^2}\sqrt{4\eta^2+\gamma^2}}{\frac{\gamma^2}{\eta^3}\sqrt{9\eta^2+4\gamma^2}} = \frac{\eta}{\gamma}\sqrt{\frac{4\eta^2+\gamma^2}{9\eta^2+4\gamma^2}} = \frac{1}{R}\sqrt{\frac{4+R^2}{9+4R^2}} \tag{25}$$

According to the assumption described in Note 1, when the overpotential dominates during the nucleation process, $R < 1$. When the above expression equals 1, *i.e.*, $|\nabla A| = |\nabla B|$, the following relation can be obtained (**Supplementary Equation (26)**).

$$\frac{|\nabla A|}{|\nabla B|} = \frac{1}{R}\sqrt{\frac{4+R^2}{9+4R^2}} = 1 \tag{26}$$

The positive solution is obtained as $R = \sqrt{\sqrt{2}-1} \approx 0.644$. When $R < 0.644$ (for example, when γ is much smaller than η), the ratio $|\nabla A| > |\nabla B|$, indicates that A has a larger gradient and a faster variation rate. This implies that the Gibbs free energy of HCP nucleation changes more rapidly, leading to a faster evolution of the corresponding nucleation surface density and a stronger influence of the overpotential.

In contrast, when $0.644 < R < 1$, the ratio $|\nabla A| < |\nabla B|$, suggesting that B has a larger gradient and a faster variation rate. Accordingly, the Gibbs free energy of spherical nucleation changes more rapidly, resulting in a faster evolution of the corresponding nucleation surface density and a more pronounced effect of the overpotential.

As shown in the figure above, when $R > 1$, the ratio $\frac{|\nabla A|}{|\nabla B|} < 1$, decreases further with increasing R. This indicates that, under surface energy dominated nucleation, the rate of change of the free-energy difference for spherical nucleation increases more rapidly, eventually exceeding that for HCP nucleation.

Supplementary Note 3. Derivation of the kinetic Butler-Volmer equation and dimensionless factors.

Supplementary Equation (27) represents the Butler-Volmer equation, which describes the relationship between the current density and electrode potential in an electrode reaction, particularly for redox processes.

$$j = j_0 \left[\exp\left(\frac{\alpha_{\mathrm{anode}}\mathrm{zF}}{\mathrm{R}T}\eta\right) - \exp\left(-\frac{\alpha_{\mathrm{cathode}}\mathrm{zF}}{\mathrm{R}T}\eta\right)\right] \tag{27}$$

Where R is the gas constant, F is the Faraday constant, and T is the temperature (in Kelvin), z denotes the number of transferred charges, α_{anode} and $\alpha_{\mathrm{cathode}}$ represent the anodic and cathodic transfer coefficients, respectively (which are approximately equal in a symmetric cell). j and j_0 represent the current density and the exchange current density, respectively. Under constant current (j) charge/discharge conditions, the relationship between j and η can be described as shown in **Supplementary Equations (28)** and **(29)**, indicating that in a symmetric cell, a higher current density leads to a larger overpotential.

$$\frac{j}{j_0} = 2\sinh\left(\frac{\alpha\mathrm{zF}}{\mathrm{R}T}\eta\right) \tag{28}$$

$$\eta = \frac{\mathrm{R}T}{\alpha\mathrm{zF}}\ln\left(\frac{j}{2j_0} + \sqrt{\left(\frac{j}{2j_0}\right)^2 + 1}\right) \tag{29}$$

Within the regime dominated by a single phase and governed by principle balance, the equation can be expressed in the form of a Tafel relation.

$$j_{\mathrm{anode}} = j_0\exp\left(\frac{\alpha_{\mathrm{anode}}\mathrm{zF}}{\mathrm{R}T}\eta\right) \tag{30}$$

$$j_{cathode} = j_0\exp\left(-\frac{\alpha_{\mathrm{cathode}}\mathrm{zF}}{\mathrm{R}T}\eta\right) \tag{31}$$

Under self-corrosion conditions and in the absence of an externally applied current, $j_{\mathrm{anode}}(\eta) = j_{\mathrm{cathode}}(\eta) = i_{\mathrm{corr}}$, where i_{corr} represents the corrosion current density. In addition, the polarization resistance is defined as the reciprocal of the small-signal slope near the polarization potential (η, overpotential).

$$Z = \left(\frac{\mathrm{d}j}{\mathrm{d}E}\right)^{-1}_{\eta\to 0} \tag{32}$$

$$\left(\frac{\mathrm{d}j}{\mathrm{d}E}\right)_{\eta\to 0} = j_0\frac{\mathrm{zF}}{\mathrm{R}T}(\alpha_{\mathrm{anode}} + \alpha_{\mathrm{cathode}}) \tag{33}$$

$$Z = \frac{\mathrm{R}T}{j_0\mathrm{zF}}\frac{1}{(\alpha_{\mathrm{anode}}+\alpha_{\mathrm{cathode}})} \tag{34}$$

The anodic and cathodic Tafel slopes are defined by **Supplementary Equations (35) and (36)**, respectively.

$$\beta_{\text{anode}} = \frac{2.303\ \text{R}T}{\alpha_{\text{anode}}\text{zF}}\ ,\ \beta_{\text{cathode}} = \frac{2.303\ \text{R}T}{\alpha_{\text{cathode}}\text{zF}} \tag{35)\&(36}$$

The units are typically expressed as millivolts per decade of current. In corrosion kinetics analysis, the anodic and cathodic polarizations are often treated as independently manifested and can be approximated as follows:

$$j_{\text{anode}} \approx j_{\text{corr}} 10^{\eta/\beta_{\text{anode}}}\ ,\ j_{\text{cathode}} \approx j_{\text{corr}} 10^{\eta/\beta_{\text{cathode}}} \tag{37)\&(38}$$

In the small-perturbation regime, the exponential terms can be expanded using a first-order approximation. Substituting into the net current expression $j = j_{\text{anode}} - j_{\text{cathode}}$, and expanding with respect to small overpotential η, **Supplementary Equation (39)** is obtained.

$$j \approx \eta j_{\text{corr}} \left[\ln(10)\left(\frac{1}{\beta_{\text{anode}}} + \frac{1}{\beta_{\text{cathode}}}\right)\right] \tag{39}$$

Therefore, the above **Supplementary Equation (33)** can be rewritten as:

$$\left(\frac{\text{d}j}{\text{d}E}\right)_{\eta\to 0} = j_{\text{corr}} \left[\ln(10)\left(\frac{1}{\beta_{\text{anode}}} + \frac{1}{\beta_{\text{cathode}}}\right)\right] \tag{40}$$

Therefore, Z can be obtained as:

$$Z = \left(\frac{\text{d}j}{\text{d}E}\right)^{-1}_{\eta\to 0} = \frac{1}{j_{\text{corr}}\ln(10)}\frac{1}{\left(\frac{1}{\beta_{\text{anode}}}+\frac{1}{\beta_{\text{cathode}}}\right)} = \frac{\beta_{\text{anode}}*\beta_{\text{cathode}}}{2.303 j_{\text{corr}}(\beta_{\text{anode}}+\beta_{\text{cathode}})} \tag{41}$$

Thus, on a unit area basis, the Stern-Geary equation is obtained:[1-3]

$$i_{\text{corr}} * Z = \frac{\beta_{\text{anode}}*\beta_{\text{cathode}}}{2.303(\beta_{\text{anode}}+\beta_{\text{cathode}})} = B \tag{42}$$

Therefore, the dimensionless factor W_{f} is defined as the ratio of the overpotential in the nucleation process to the product of the corrosion current and the polarization resistance (**Supplementary Equation (34)**).

$$W_{\text{f}} = \frac{\Delta E}{I\times Z} = \frac{\eta}{B} \tag{43}$$

Here, ΔE denotes the nucleation overpotential (η), I the corrosion current, and Z the polarization resistance.

Electrochemical impedance spectra can often be approximated using a simple equivalent circuit. A commonly adopted model consists of the solution resistance R_{s} in series with a parallel combination of the charge transfer resistance R_{ct} and the double-layer capacitance C_{dl} , as shown in **Supplementary Equation (44)**.

$$Z(\omega) = R_{\text{s}} + \frac{1}{\frac{1}{R_{\text{ct}}}+j\omega C_{\text{dl}}} = R_{\text{s}} + \frac{R_{\text{ct}}}{1+j\omega R_{\text{ct}}C_{\text{dl}}} \tag{44}$$

Here ω denotes the angular frequency. In the high-frequency limit, the magnitude of the second term tends to zero, so $Z(\omega \to \infty) \approx R_{\text{s}}$. In the low-frequency limit, $Z(\omega \to 0) \approx R_{\text{s}} + R_{\text{ct}}$. Because R_{ct} is much larger than R_{s} in the present system (Zn||Zn asymmetric cells), the contribution of R_{s} can be neglected, the polarization resistance therefore dominates the low-frequency real part of the impedance. Under typical conditions, in which no significant diffusion-controlled processes or pseudocapacitive behavior are present, and an ideal capacitor can approximate the interfacial

capacitance, this polarization resistance is equivalent to the charge-transfer resistance obtained from fitting, namely $Z \approx R_{\mathrm{ct}}$, as described in Supplementary Equation (45).[3-5]

$$W_{\mathrm{f}} = \frac{\eta}{i_{\mathrm{corr}} \times R_{\mathrm{ct}}} \tag{45}$$

To examine its dimensionless nature, η has units of V, i_{corr} has units of A, and R_{ct} has units of Ω. Each term is measured per unit area. Substituting these into **Supplementary Equation (46)** confirms that the quantity is properly normalized and thus satisfies the definition of a dimensionless factor.

$$[W_{\mathrm{f}}] = \frac{V}{(A)(\Omega)} = 1 \tag{46}$$

Supplementary Figures

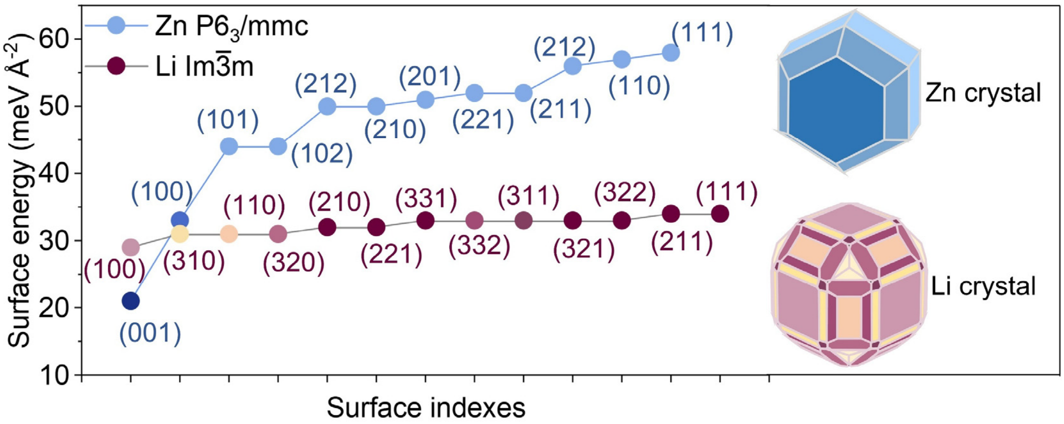

Supplementary Fig. 1 The surface energies of the various crystal planes of Li metal and Zn metal. The distinct color blocks in the figure correspond to the specific crystal planes of the respective metals.

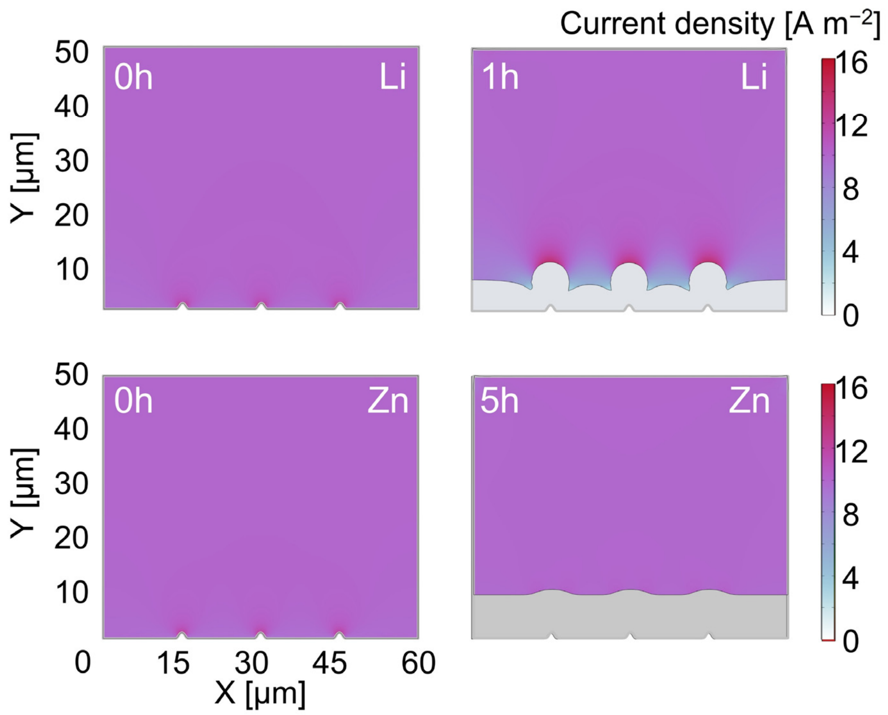

Supplementary Fig. 2 Simulation of the electric field distribution after a period of electroplating of Li metal and Zn metal. Tracking the evolution of the deformed boundaries throughout the deposition process reveals that the Li anode exhibits a non-uniform current density.

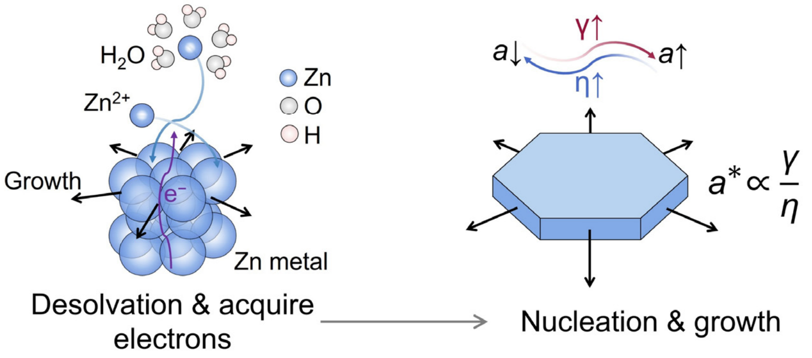

Supplementary Fig. 3 Schematic diagram of nucleation and growth process. As depicted in the model, the critical nucleation size (a^*) exhibits a direct proportionality to the interfacial surface energy (γ), while demonstrating an inverse dependence on the applied overpotential (η).

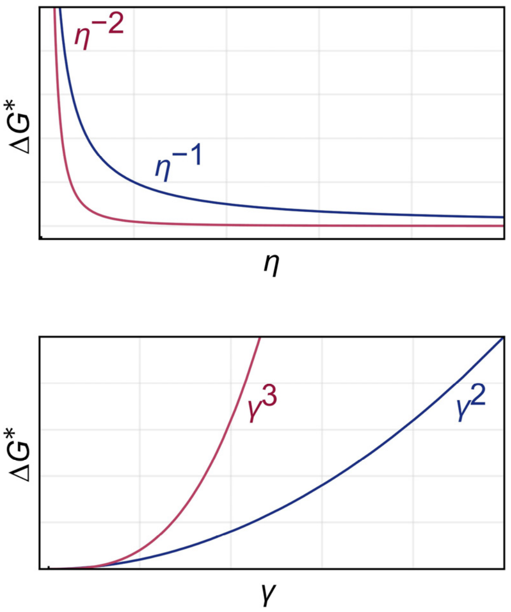

Supplementary Fig. 4 Variation of the critical nucleation free energy (ΔG^*) under different nucleation models. The analysis investigates the dependence of ΔG^* on an individual thermodynamic parameter. It highlights the distinct mathematical relationships when evaluated against either the interfacial surface energy (γ) or the applied overpotential (η).

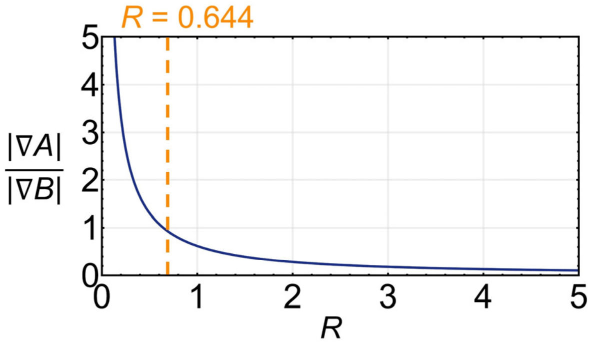

Supplementary Fig. 5 Relationship between the ratio *R* and $\frac{|\nabla \boldsymbol{A}|}{|\nabla \boldsymbol{B}|}$ within the range of 0-5, illustrating their correlated variation. When $R = 0.644$, the corresponding value of $\frac{|\nabla A|}{|\nabla B|}$ reaches 1, indicating that the rates of free energy change for the two nucleation models are identical at this point.

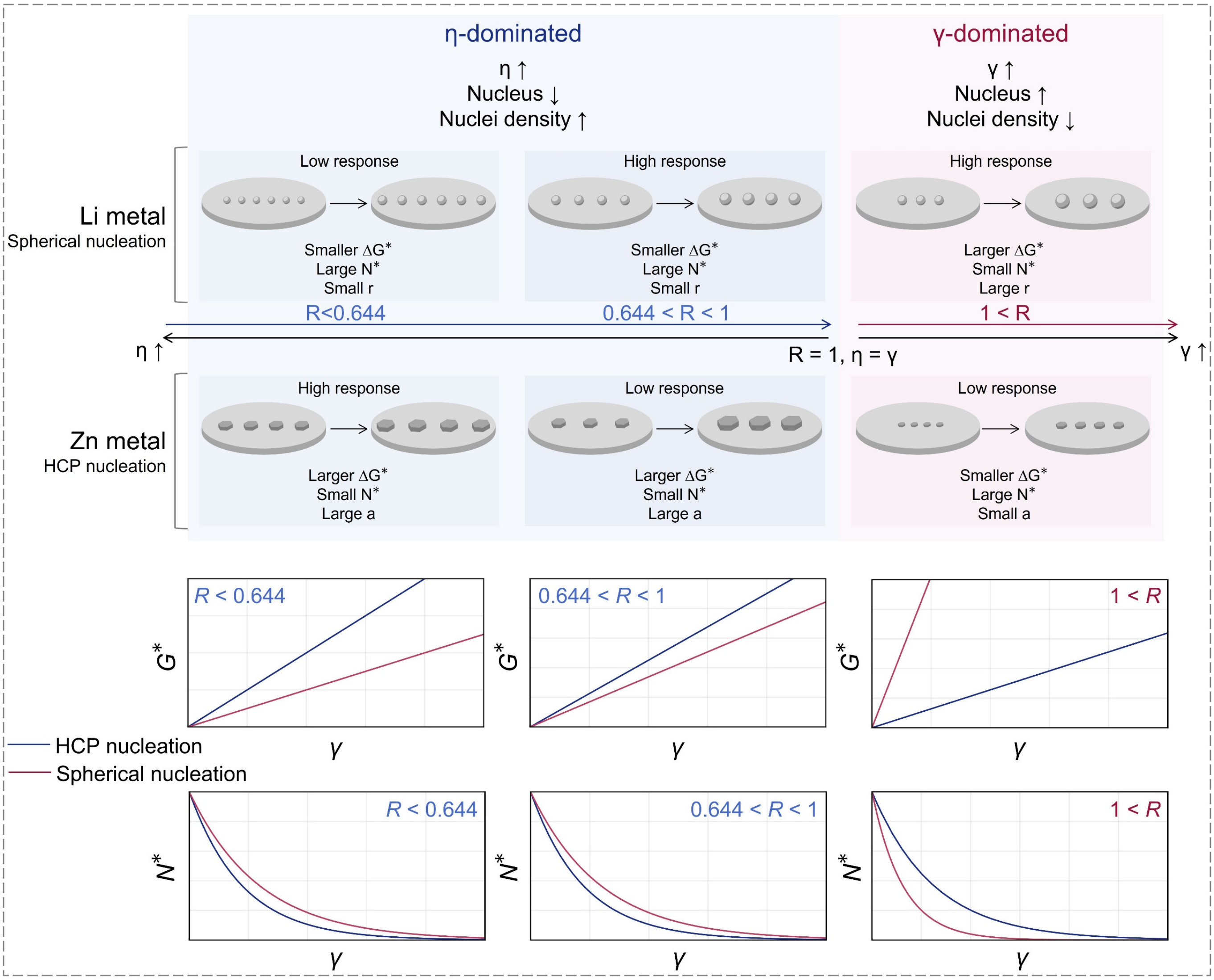

Supplementary Fig. 6 Schematic models and thermodynamic relationships for spherical and HCP nucleation processes.

The diagrams illustrate the nucleation and subsequent growth behaviors under various R values, accompanied by the numerical relationships between the critical free-energy difference (ΔG^*) and the critical nuclei density (N^*). As depicted, the stability of metal deposition is fundamentally governed by the critical nucleus size (a or r) and N^*. Specifically, a higher nucleation density, driven by a larger overpotential or a lower surface energy, provides more available nucleation sites, thereby promoting uniform deposition. Concurrently, a smaller critical nucleus radius effectively mitigates the risk of large dendritic structures forming and penetrating the separator. Consequently, under ideal planar nucleation, these models indicate that larger applied currents tend to favor a highly homogeneous electrodeposition interface.

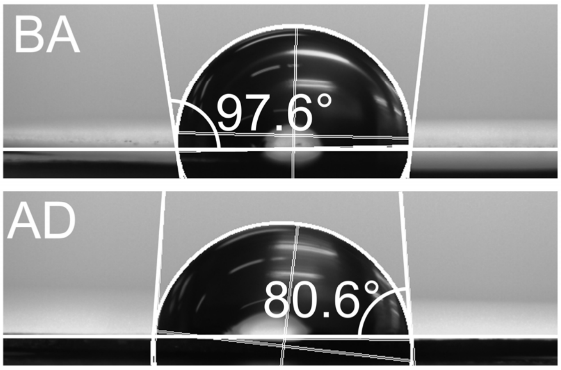

Supplementary Fig. 7 Contact angle test between BA and AD electrolytes. Compared to the BA electrolyte, the AD electrolyte exhibits a decreased contact angle.

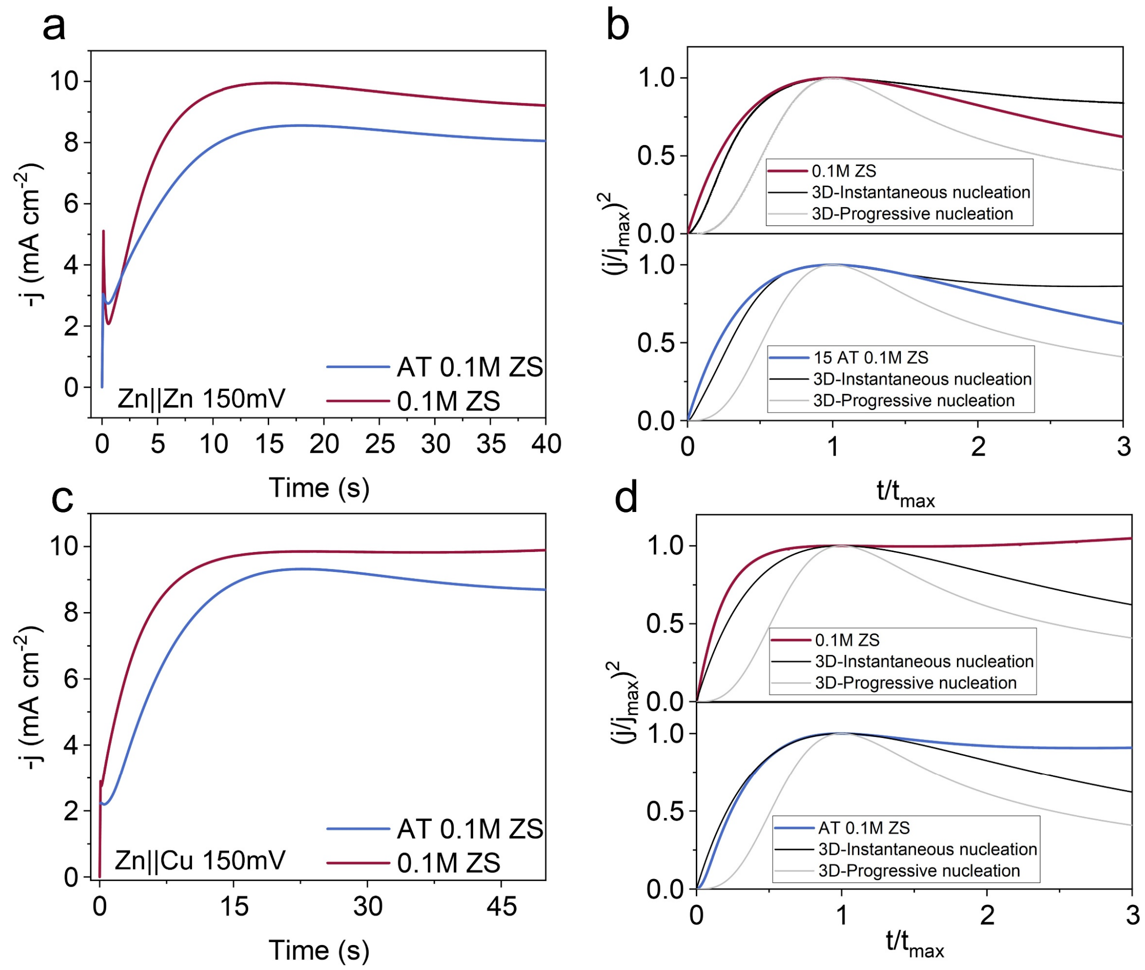

Supplementary Fig. 8 Chronoamperometry (CA) analysis of Zn||Zn (100 μm) and Zn||Cu cells with 0.1 M ZS and AD (15 mM AT)-0.1 M ZS electrolytes. Chronoamperometry (CA) curves of (**a**) Zn||Zn and (**b**) Zn||Cu cells. Nucleation behavior analysis based on the Scharifker-Hills model for (**c**) Zn||Zn and (**d**) Zn||Cu cells.[6] All electrochemical tests in this figure are conducted at 25 ± 2 °C.

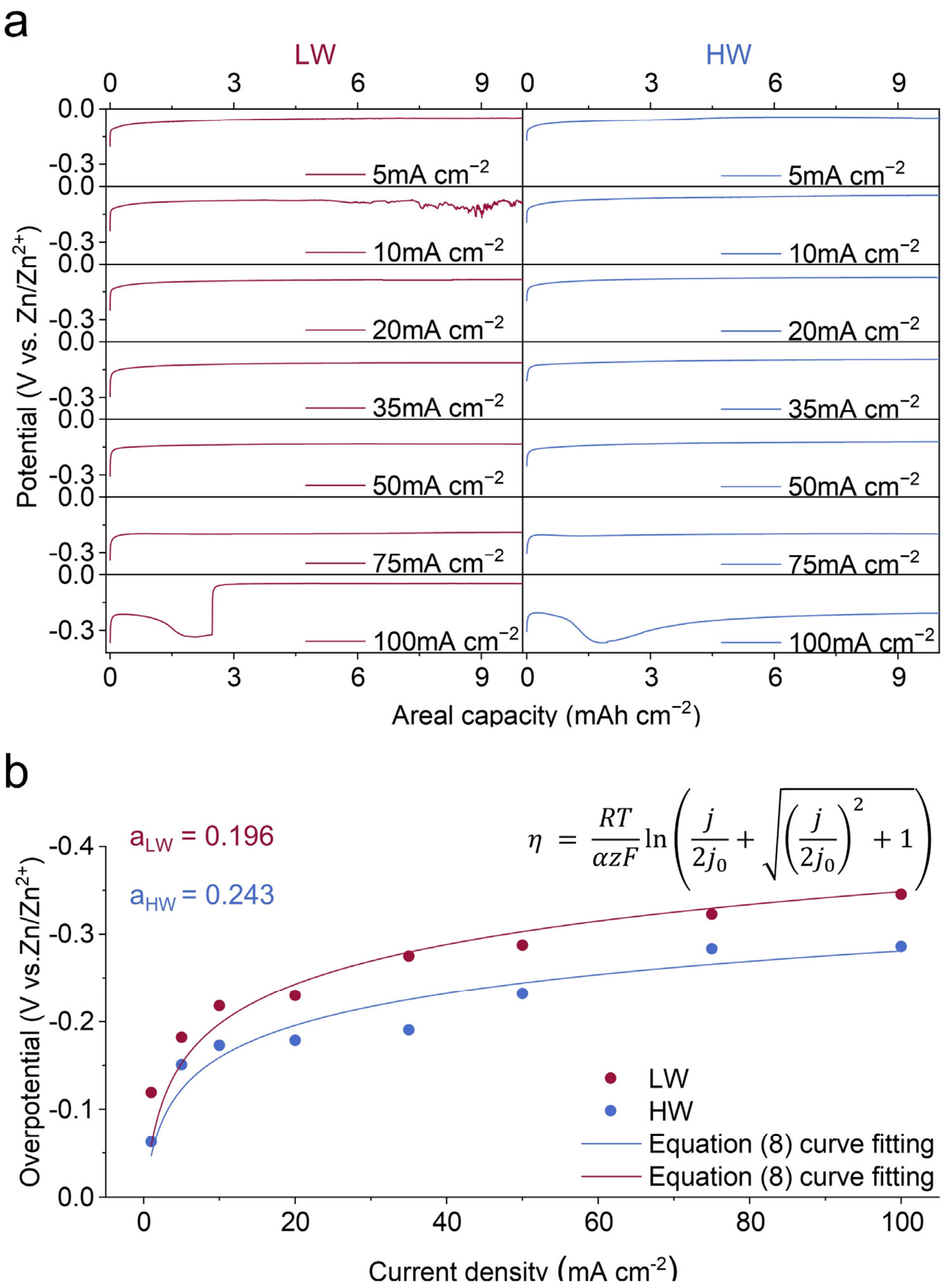

Supplementary Fig. 9 Electrochemical kinetics and nucleation overpotential analysis of the Zn anodes. **a** Voltage curves of Zn||Zn (100 μm) symmetric cells using LW and HW electrolytes at current densities of 5, 10, 20, 35, 50, 75, and 100 mA cm^{-2}. **b** Curve fitting of the nucleation overpotential as a function of current density based on Equation (8) (Supplementary Equation (29)). All electrochemical tests in this figure are conducted at 25 ± 2 °C.

By analyzing the dependence of the nucleation overpotential on current density, we extracted the interfacial charge-transfer coefficient for the HW electrolyte as $\alpha_{HW} = 0.243$, which is larger than that for the LW electrolyte ($\alpha_{LW} = 0.196$). This difference indicates faster interfacial charge-transfer kinetics in the HW electrolyte.

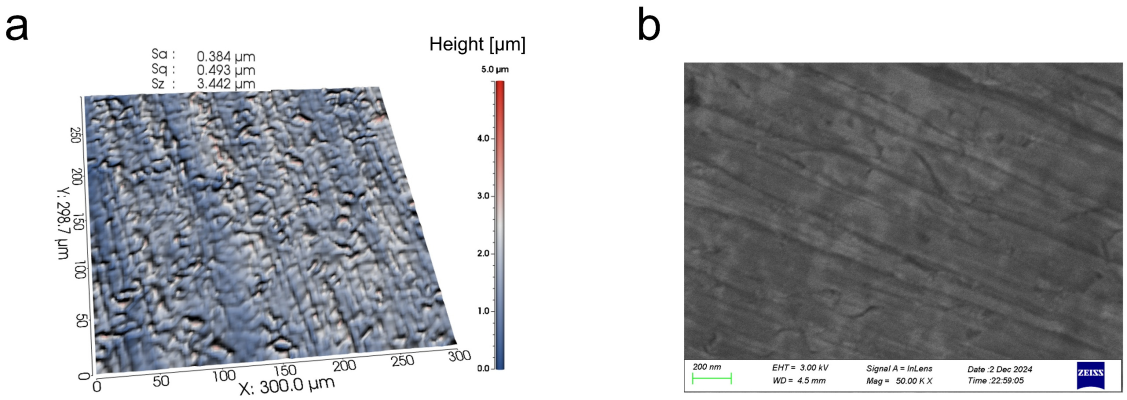

Supplementary Fig. 10 Surface morphology of the Zn foil. a Confocal microscopy image of the Zn foil, with a scanned area of 300µm × 300µm. **b** Scanning electron microscopy (SEM) image displaying the surface topography of the Zn metal electrode.

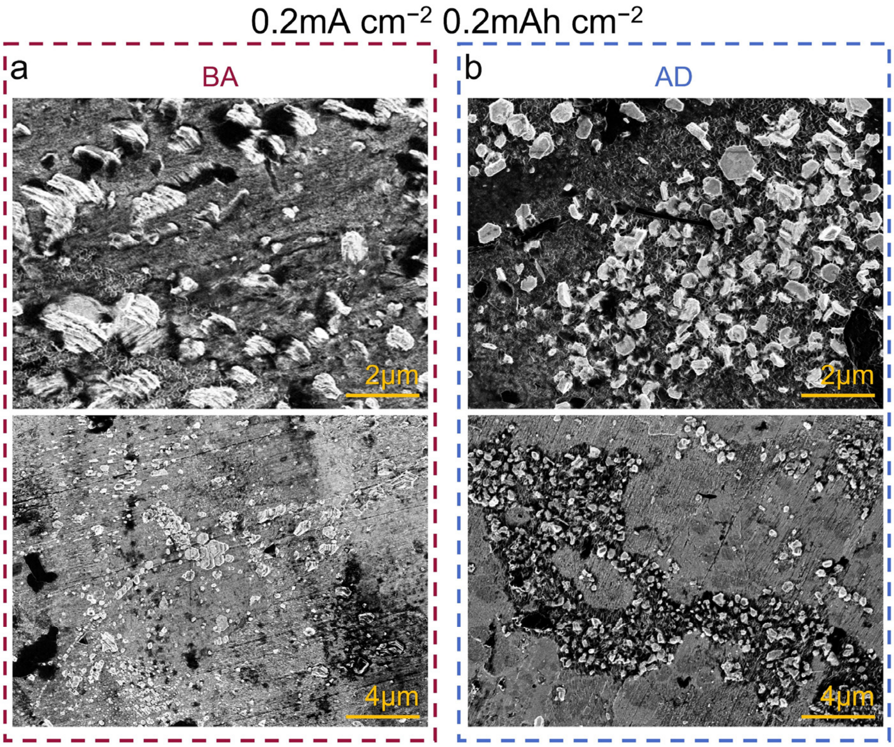

Supplementary Fig. 11 SEM images of Zn crystal nuclei electrodeposited in the BA (a) and AD (b) electrolytes. The electrodeposition is performed on a pristine Zn foil working electrode (observation electrode). The plating process is controlled at a constant current density of 0.2 mA cm^{-2} with an areal capacity of 0.2 mAh cm^{-2} under an ambient temperature of 25 ± 2 °C.

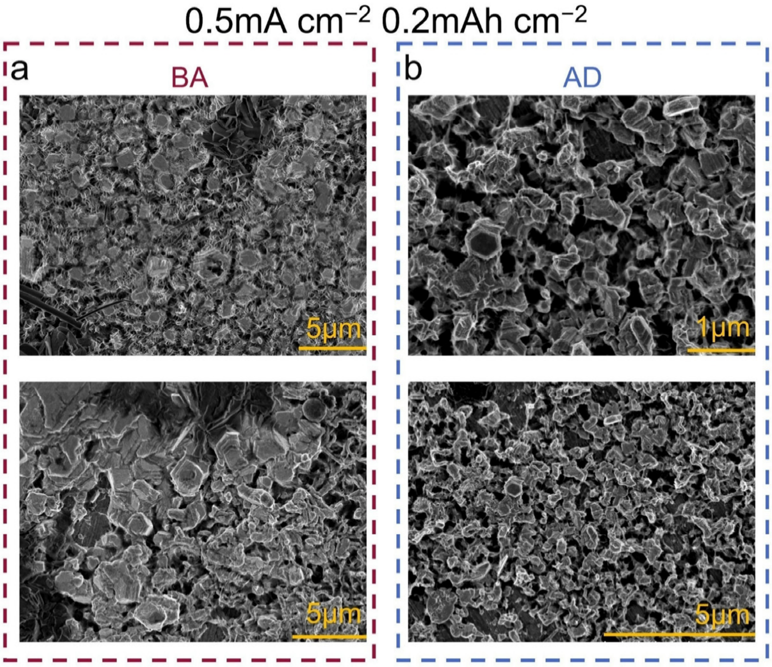

Supplementary Fig. 12 SEM images of Zn crystal nuclei electrodeposited in the BA (a) and AD (b) electrolytes. The electrodeposition is performed on a pristine Zn foil working electrode (observation electrode). The plating process is controlled at a constant current density of 0.5 mA cm^{-2} with an areal capacity of 0.2 mAh cm^{-2} under an ambient temperature of 25 ± 2 ℃.

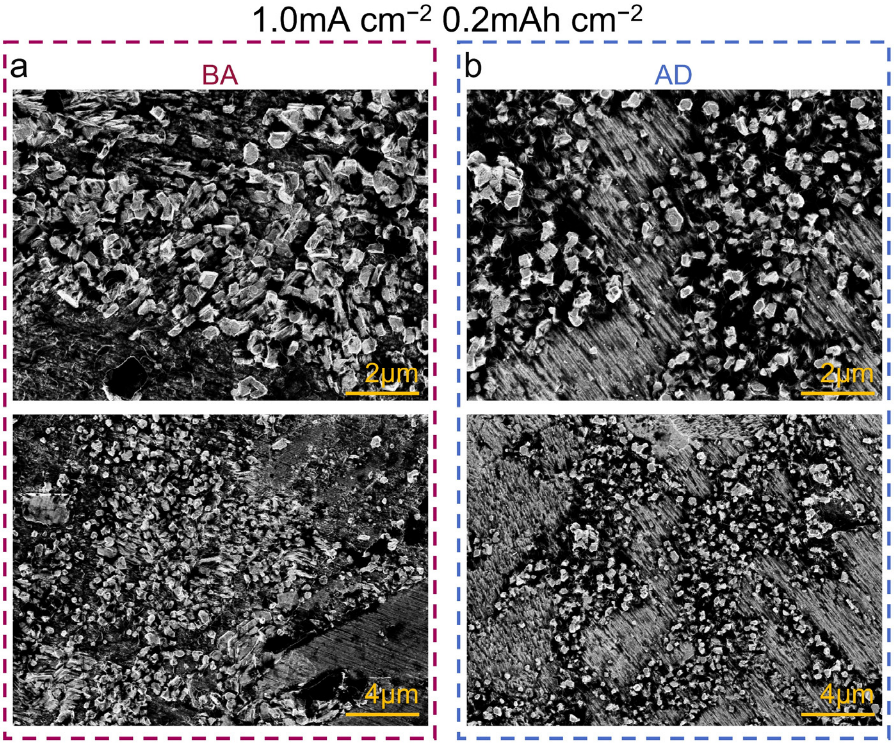

Supplementary Fig. 13 SEM images of Zn crystal nuclei electrodeposited in the BA (a) and AD (b) electrolytes. The electrodeposition is performed on a pristine Zn foil working electrode (observation electrode). The plating process is controlled at a constant current density of 1.0 mA cm^{-2} with an areal capacity of 0.2 mAh cm^{-2} under an ambient temperature of 25 ± 2 °C.

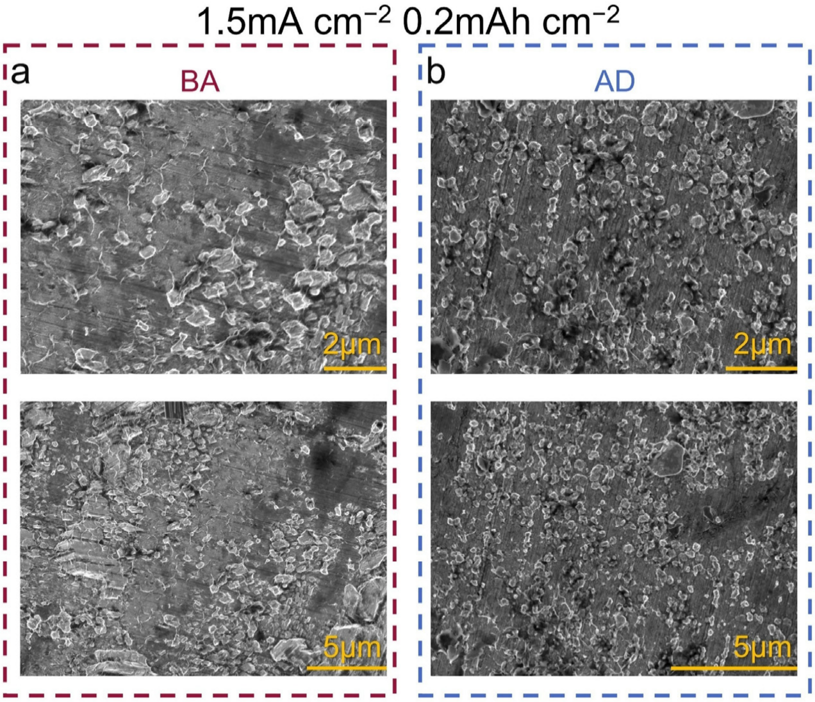

Supplementary Fig. 14 SEM images of Zn crystal nuclei electrodeposited in the BA (a) and AD (b) electrolytes. The electrodeposition is performed on a pristine Zn foil working electrode (observation electrode). The plating process is controlled at a constant current density of 1.5 mA cm^{-2} with an areal capacity of 0.2 mAh cm^{-2} under an ambient temperature of 25 ± 2 °C.

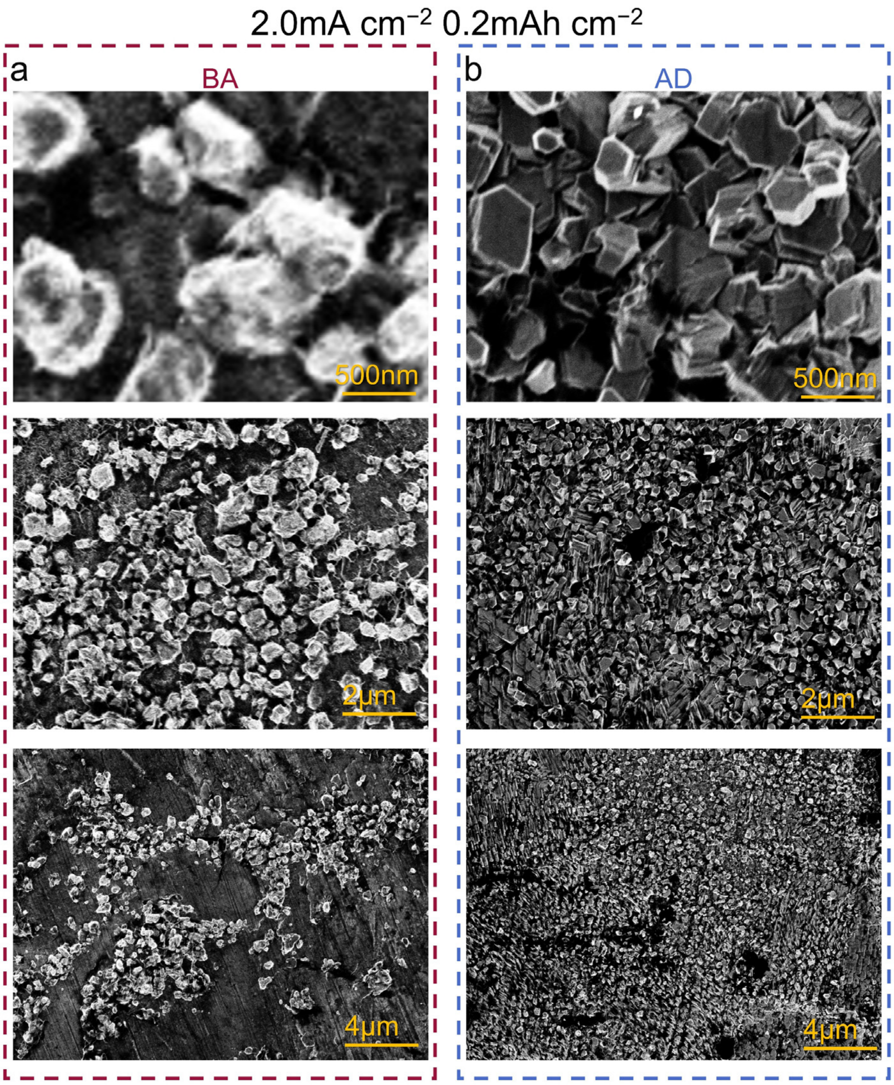

Supplementary Fig. 15 SEM images of Zn crystal nuclei electrodeposited in the BA (a) and AD (b) electrolytes. The electrodeposition is performed on a pristine Zn foil working electrode (observation electrode). The plating process is controlled at a constant current density of 2.0 mA cm^{-2} with an areal capacity of 0.2 mAh cm^{-2} under an ambient temperature of 25 ± 2 °C.

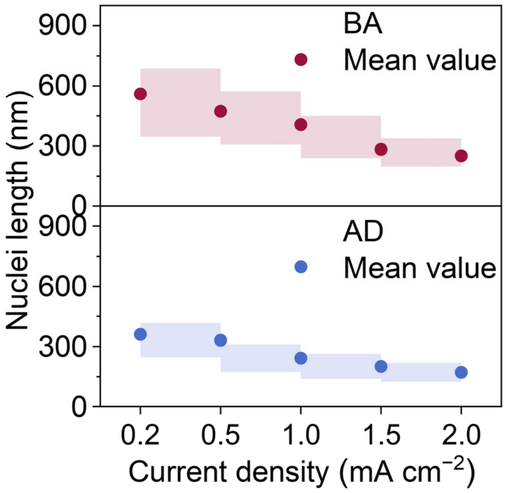

Supplementary Fig. 16 The average size and variance of Zn crystal nuclei during electro-deposition at different current densities (0.2, 0.5, 1.0, 1.5, and 2.0 mA cm^{-2}) to achieve a surface capacity of 0.2 mAh cm^{-2}.

The sample sizes (n, representing the number of statistically counted crystal nuclei) for BA at current densities of 0.2, 0.5, 1.0, 1.5, and 2.0 mA cm^{-2} are 204, 181, 222, 112, and 151, respectively. For AD, the corresponding sample sizes are 201, 186, 152, 130, and 156, respectively. Data points represent the mean values, and error bars indicate the standard deviation (SD). All electrochemical tests in this figure are conducted at 25 ± 2 °C.

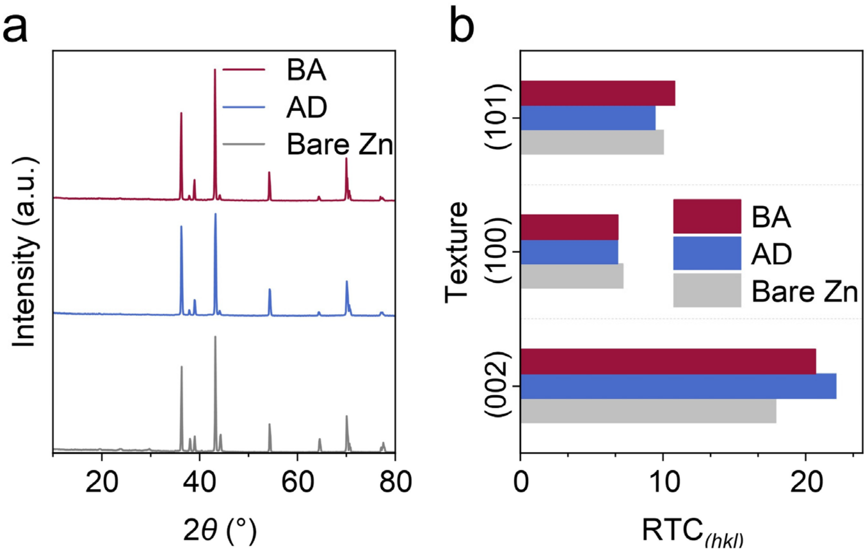

Supplementary Fig. 17 XRD patterns and corresponding RTC analysis of the deposited Zn. a XRD spectrum of the Zn foil surface after deposition of 0.2mAh cm^{-2}. **b** The derived Relative Texture Coefficient (RTC) values for the major Zn crystal planes. The RTC analysis highlights the specific textural evolution and orientation of the Zn nuclei during the early stages of electrodeposition.

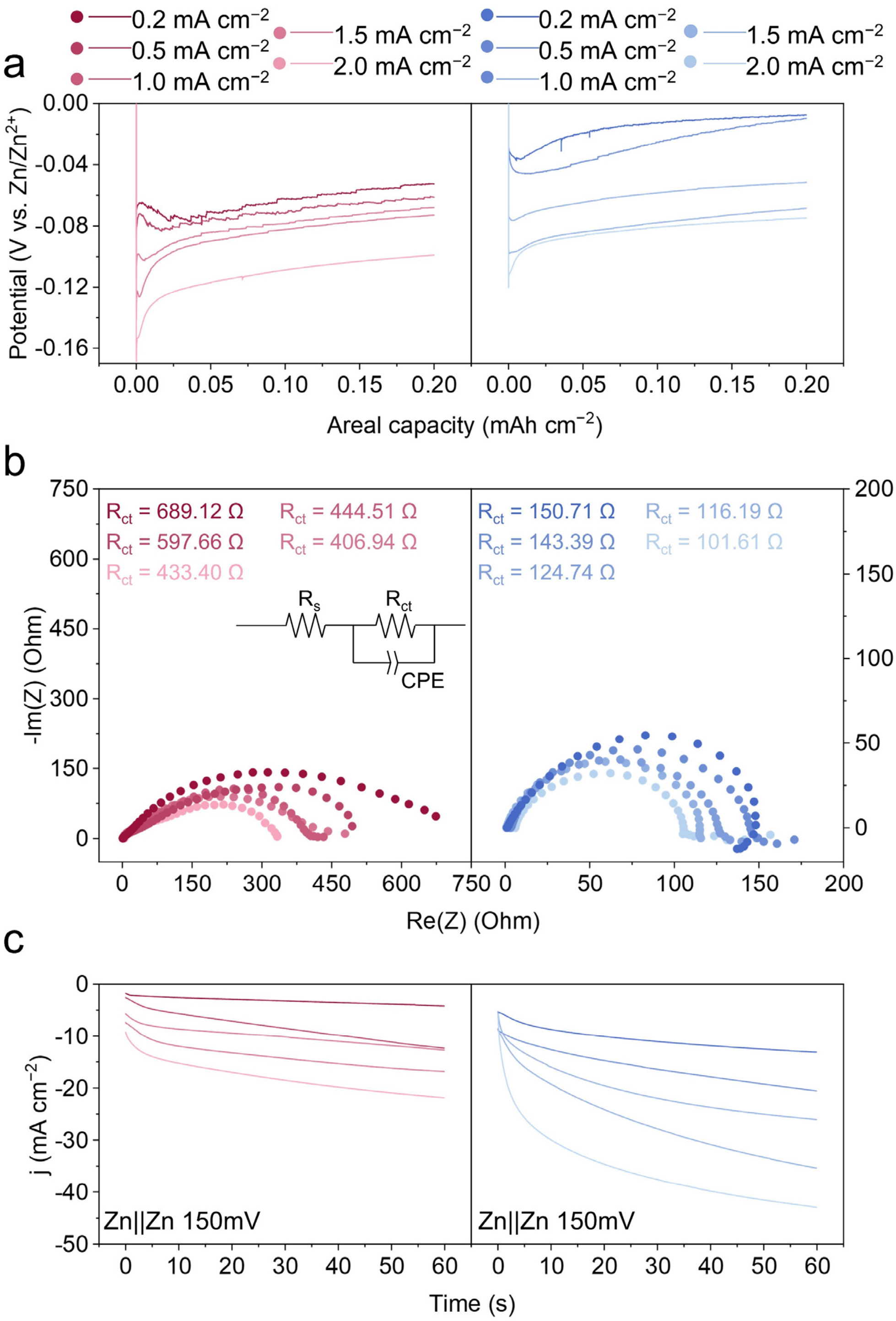

Supplementary Fig. 18 Electrochemical characterization of initial nucleation kinetics. Zn||Zn (100 μm) symmetric cells with a deposition capacity of 0.2 mAh cm^{-2} (0.2, 0.5, 1.0, 1.5, and 2.0 mA cm^{-2}) (**a**) deposition voltage curves (**b**) electrochemical impedance spectroscopy (EIS) measured after deposition (**c**) chronoamperometric (CA) current-time curves recorded after deposition. All electrochemical tests in this figure are conducted at 25 ± 2 °C.

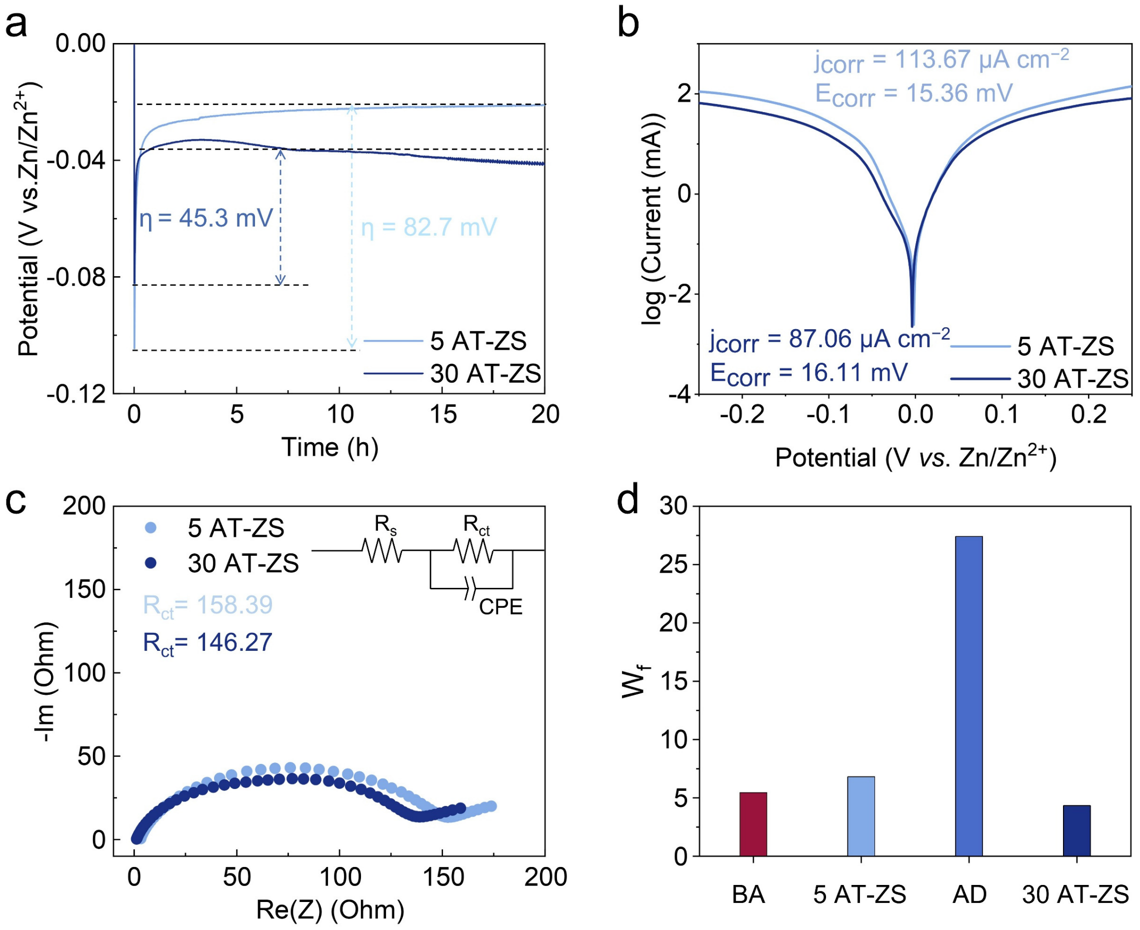

Supplementary Fig. 19 Evaluation of W_f in different electrolytes. a The nucleation overpotential curves of the 5 mM AT-ZS and 30 mM AT-ZS electrolytes Zn symmetric cells. **b** Tafel polarization curves of 5 mM AT-ZS and 30 mM AT-ZS electrolytes. **c** Impedance spectra of Zn-Zn symmetric cells in 5 mM AT-ZS and 30 mM AT-ZS electrolytes **d** Dimensionless calculations for different addition amounts of ammonia tartrate (0mM, 5mM, 15mM and 30mM). All electrochemical tests in this figure are conducted at 25 ± 2 °C.

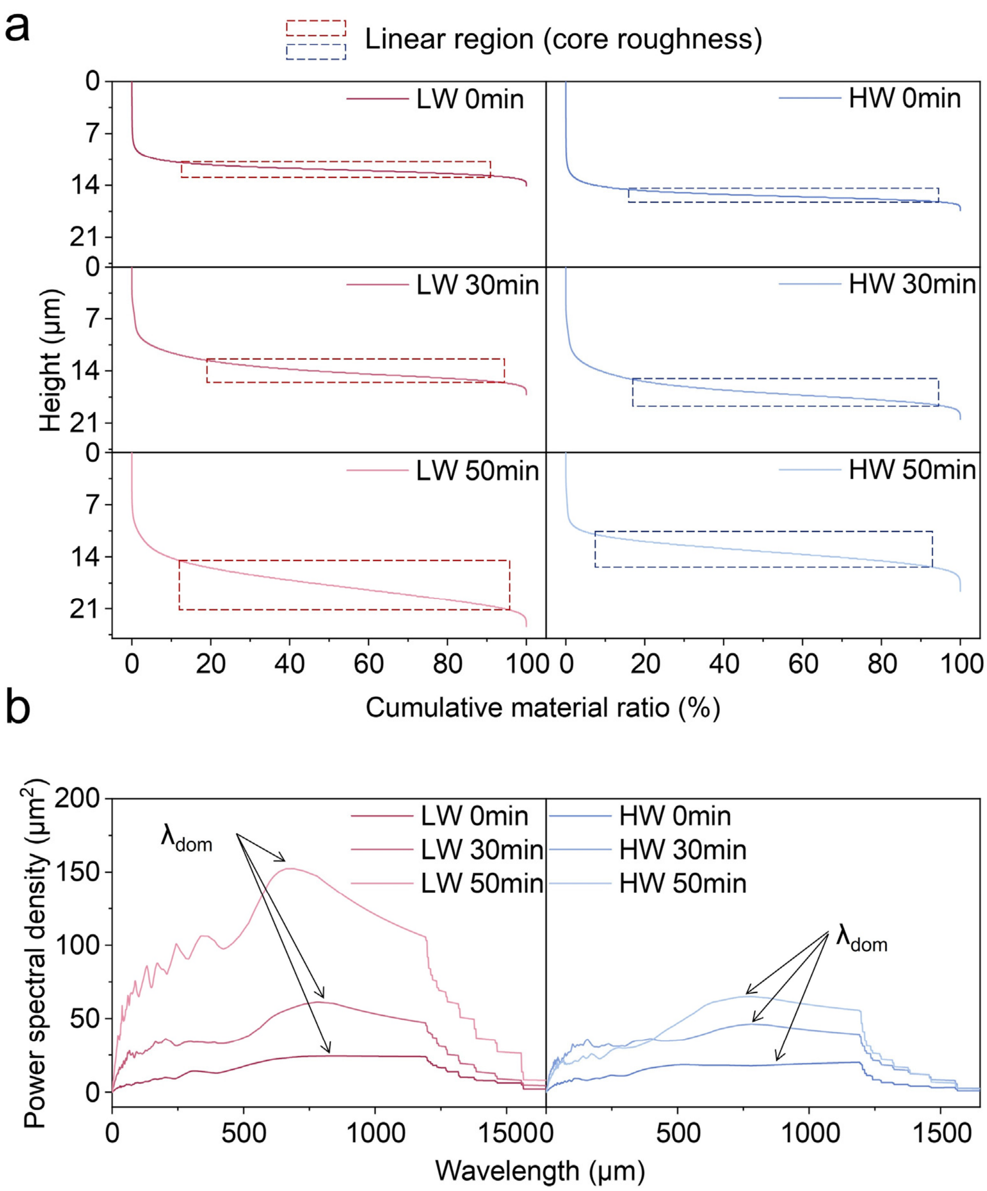

Supplementary Fig. 20 Statistical and frequency-domain analysis of the Zn surface topography. **a** Abbott-Firestone bearing area curve analysis showing the core roughness depth (Sk), together with the reduced peak height (Spk), reduced valley depth (Svk), and the upper and lower material ratios (Mr1 and Mr2), which collectively quantify the height distribution and uniformity of Zn deposition. **b** Power spectral density (PSD) analysis of the surface height profile, from which the dominant spatial wavelength (λ_{dom}) and the maximum PSD amplitude ($A_{PSD,max}$) are extracted to characterize the characteristic length scale and intensity of surface height fluctuations.

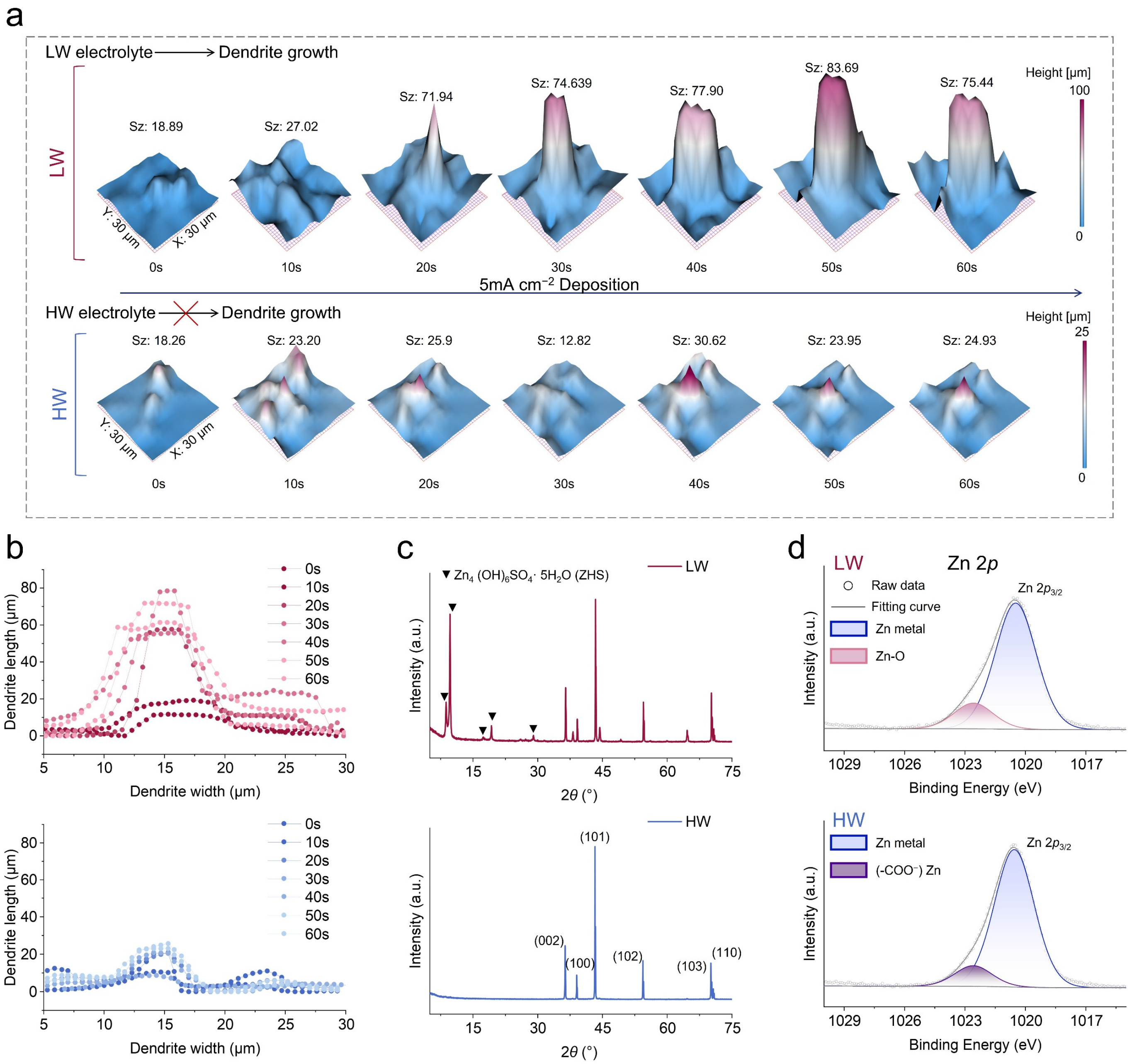

Supplementary Fig. 21 In situ morphological evolution and interfacial composition analysis of Zn deposition. Three-dimensional reconstructed image (**a**) and cross-sectional profile (**b**) of in situ dendrite growth. The images are obtained by in situ laser confocal microscopy in a Zn||Zn (100 μm) symmetric cell under a current density of 5 mA cm^{-2}, with surface reconstruction performed at 10 s intervals corresponding to a capacity increment of 0.0139 mAh cm^{-2} per frame. Post-deposition XRD (**c**) and XPS (**d**) spectra indicate the formation of basic zinc sulfate (ZHS) in the LW electrolyte. In contrast, the HW electrolyte containing the additive exhibits an interfacial protective layer with no detectable parasitic reaction products. All electrochemical tests in this figure are conducted at 25 ± 2 ℃.

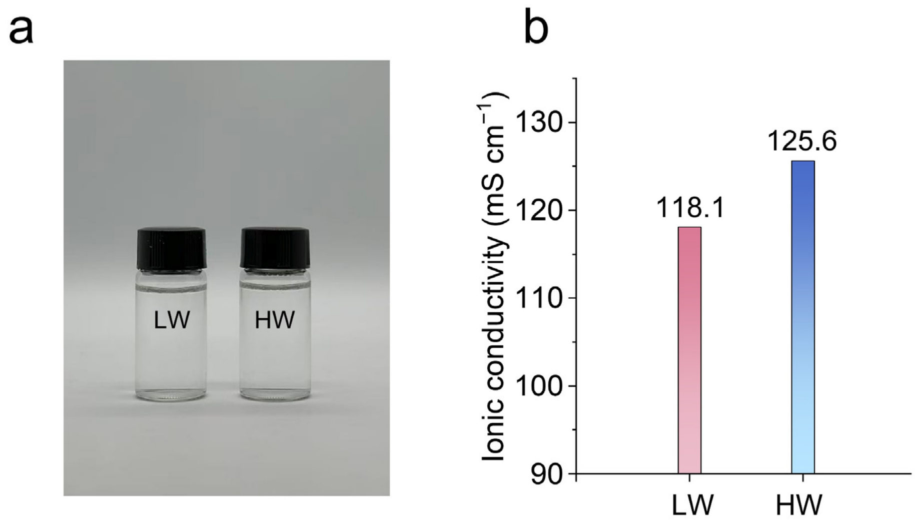

Supplementary Fig. 22 Basic characterization of the LW and HW electrolytes. a Optical photographs of the LW and HW electrolytes. **b** Comparison of the ionic conductivities between the two different electrolyte systems.

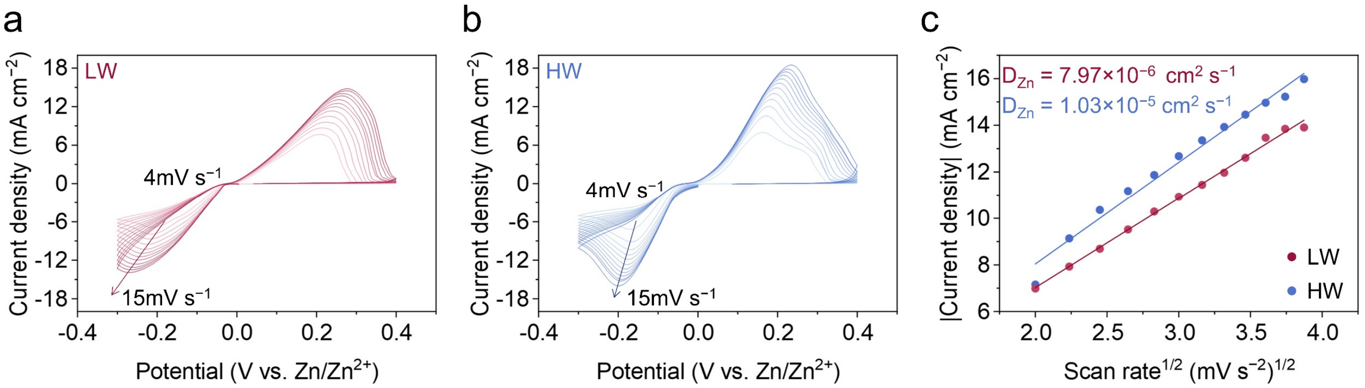

Supplementary Fig. 23 The CV tests of Zn||Cu asymmetric cells with (**a**) LW electrolyte (0.05 M $ZnSO_4$) and (**b**) HW electrolyte (15 mM AT-0.05 M $ZnSO_4$), and (**c**) the corresponding diffusion coefficient calculated from simplified Randles-Sercik curves. The scan rate is from 4 to 15 mV s^{-1}. All electrochemical tests in this figure are conducted at 25 ± 2 °C.

Based on the simplified Randles-Sevcik equation (**Supplementary Equation (47)**), the Zn^{2+} diffusion coefficient ($D_{Zn}{}^{2+}$) is determined by fitting the peak currents obtained from cyclic voltammetry measurements of Zn||Cu asymmetric cells at varying scan rates.

$$I_p = 269000n^{\frac{3}{2}}ACD^{\frac{1}{2}}v^{\frac{1}{2}} \quad \textbf{(47)}$$

where D represents the ion diffusion coefficient, I_p is the peak current, v denotes the scan rate, C is the concentration of electrolyte ions, n is the number of electrons transferred in the redox reaction, and A is the electrode area.

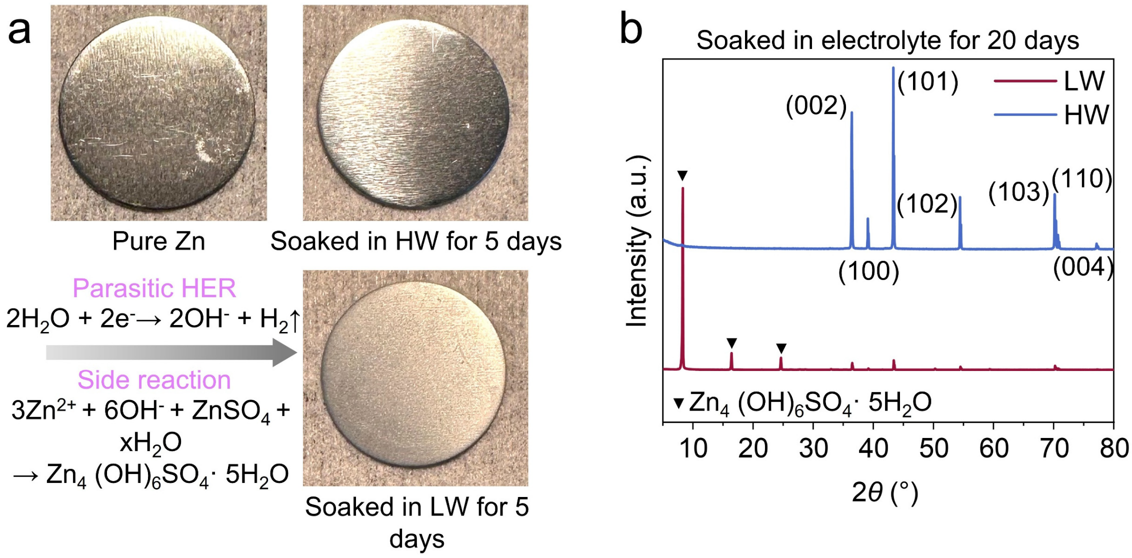

Supplementary Fig. 24 Surface changes of Zn metal after long-term immersion. a Optical images of pure Zn foil immersed in LW and HW electrolytes for 5 days, obvious corrosion layers appeared on the Zn foil in the LW. **b** XRD curves of Zn electrodes after soaking in different electrolytes for 20 days. All tests are conducted at 25 ± 2 °C.

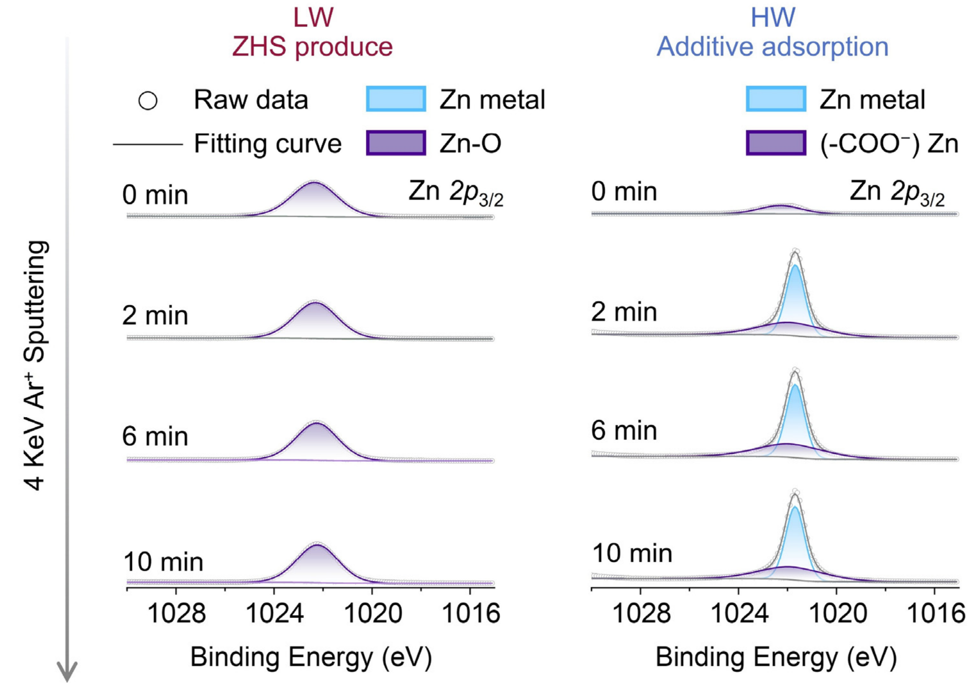

Supplementary Fig. 25 XPS depth profiling of the Zn anodes. Zn 2*p* XPS spectra of the Zn metal after 5 days of immersion in the LW and HW electrolytes. The spectra are collected at corresponding Ar^+ sputtering times of 0, 2, 6, and 10 min using an ion beam energy of 4 keV.

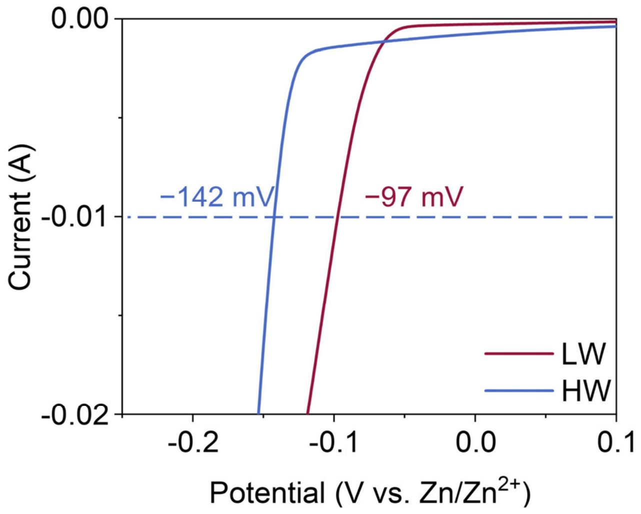

Supplementary Fig. 26 The hydrogen evolution potential curves of LW and HW electrolytes in the Zn (100 μm)||Ti asymmetric cell. The electrochemical test is conducted at 25 ± 2 ℃.

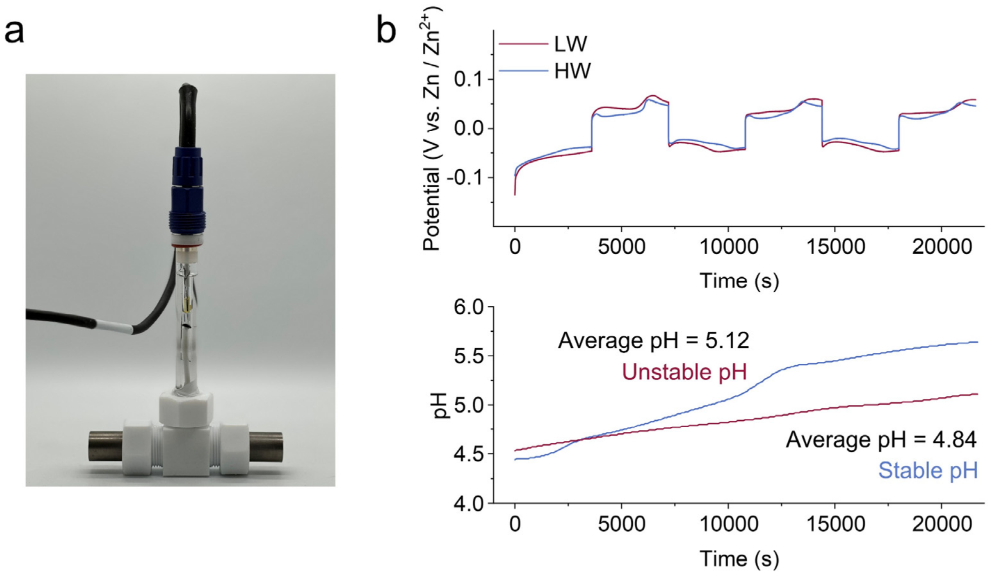

Supplementary Fig. 27 Local electrolyte pH variations during long-term charge-discharge. a Optical image of the in situ long-term effects of the local pH-shifts measurement setup. Voltage curves of Zn||Zn (100 µm) symmetric cells in (**b**) LW electrolyte and HW electrolyte at a current density of 1 mA cm^{-2}, together with the corresponding evolution of in situ local pH. The electrochemical test is conducted at 25 ± 2 ℃.

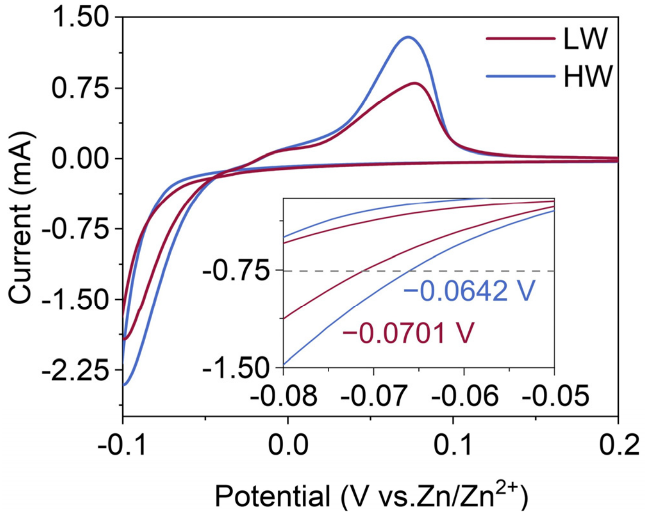

Supplementary Fig. 28 The CV curves of LW and HW electrolytes in Zn||Zn (100 μm) symmetrical batteries. The electrochemical test is conducted at 25 ± 2 °C.

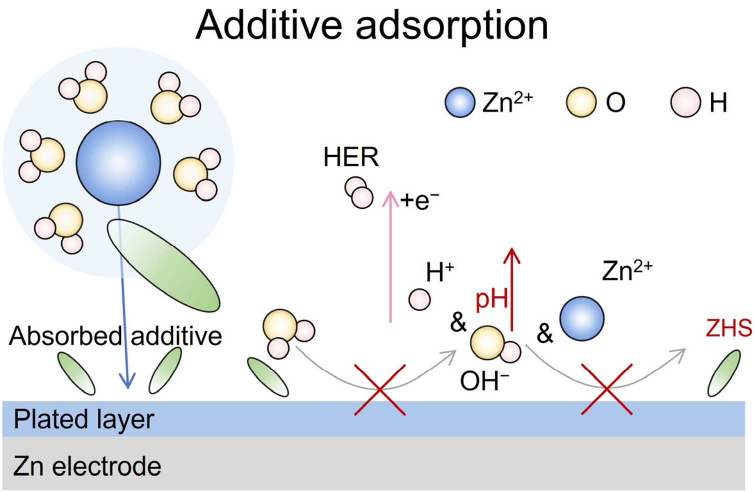

Supplementary Fig. 29 Schematic illustration of an additive-induced protective interphase on the Zn electrode.

The additive molecules preferentially adsorb on the Zn surface to construct a dense and robust interphase, which effectively screens water molecules from direct contact with Zn. This interphase suppresses the hydrogen evolution reaction (HER) and other parasitic side reactions, regulates interfacial ion transport, promotes homogeneous Zn^{2+} deposition, and thereby inhibits dendritic growth.

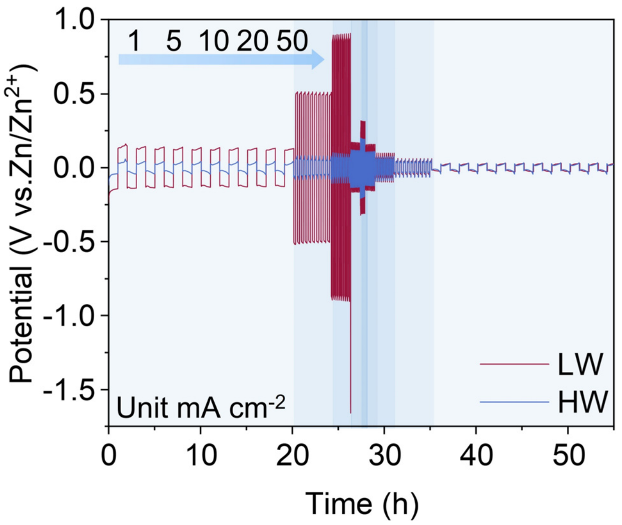

Supplementary Fig. 30 The voltage curves of LW and HW electrolytes in Zn||Zn (100 μm) symmetrical batteries. The electrochemical test is conducted at 25 ± 2 °C.

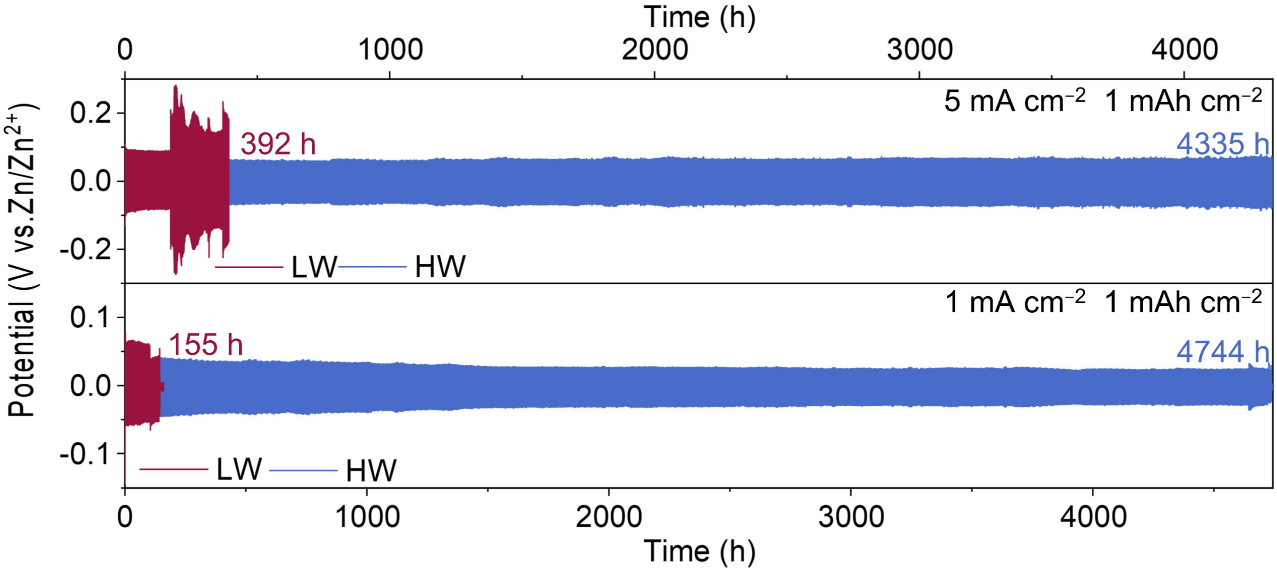

Supplementary Fig. 31 Voltage curves of Zn||Zn (100 μm) symmetric cells with LW and HW electrolytes tested at 1 and 5 mA cm^{-2} under a unit areal capacity of 1 mAh cm^{-2}. The electrochemical test is conducted at 25 ± 2 °C.

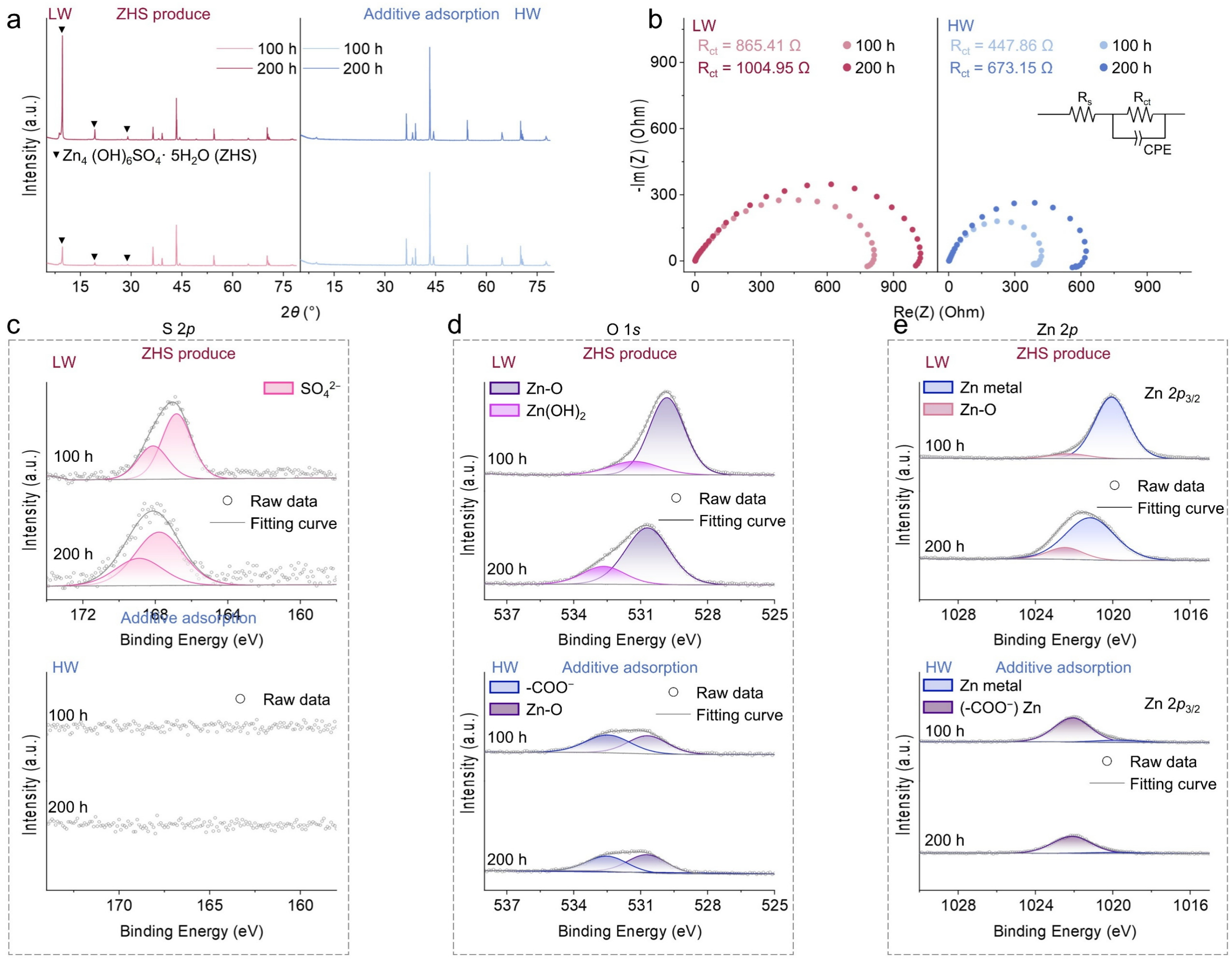

Supplementary Fig. 32 Interfacial composition analysis and kinetic evolution of Zn anodes during extended plating/stripping. XRD (**a**), electrochemical impedance spectroscopy (EIS) (**b**), and XPS spectra of S 2*p* (**c**), C 1*s* (**d**), and Zn 2*p* (e) collected after 100 h and 200 h Zn plating/stripping for cells using LW and HW electrolytes. The cells are tested for Zn plating/stripping at 1 mA cm^{-2} with an areal capacity of 1 mAh cm^{-2}. These measurements probe the evolution of interfacial dynamics and composition over multiple plating/stripping stages and demonstrate the additive's adsorption-mediated protective effect on the electrode/electrolyte interface. All electrochemical tests in this figure are conducted at 25 ± 2 °C.

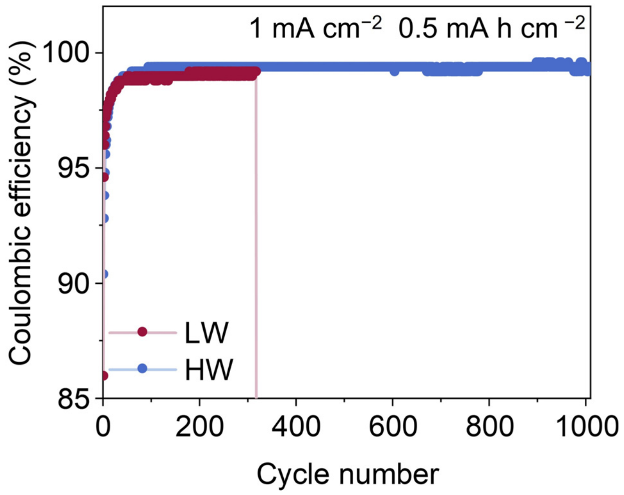

Supplementary Fig. 33 The CE test of LW and HW electrolytes in Zn(50 μm)||Cu(10 μm) half cells. The electrochemical test is conducted at 25 ± 2 °C.

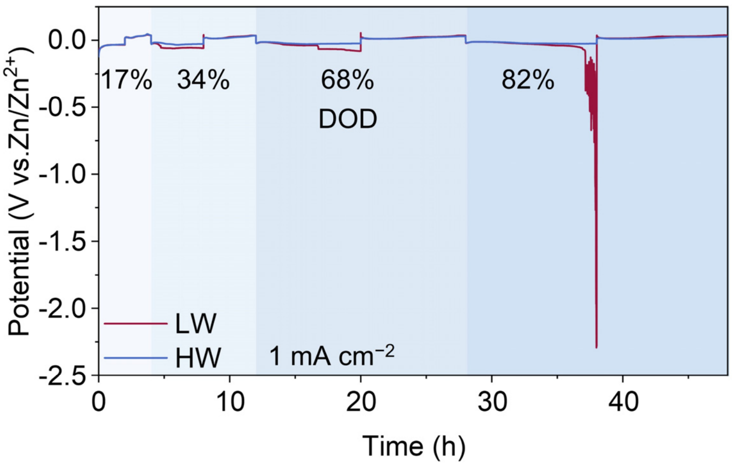

Supplementary Fig. 34 Depth of discharge (DOD) curves of LW and HW electrolytes in Zn(20 μm)||Zn(20 μm) symmetrical cells. The cells are rested for 1 h before testing. The electrochemical test is conducted at 25 ± 2 °C.

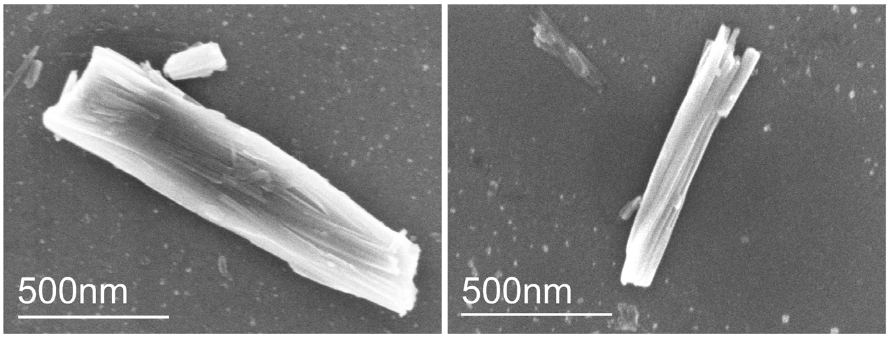

Supplementary Fig. 35 SEM characterization of the NaV_3O_8 nanorods. The scanning electron microscopy (SEM) images reveal the characteristic nanorod-like morphology of the as-synthesized NaV_3O_8 material.

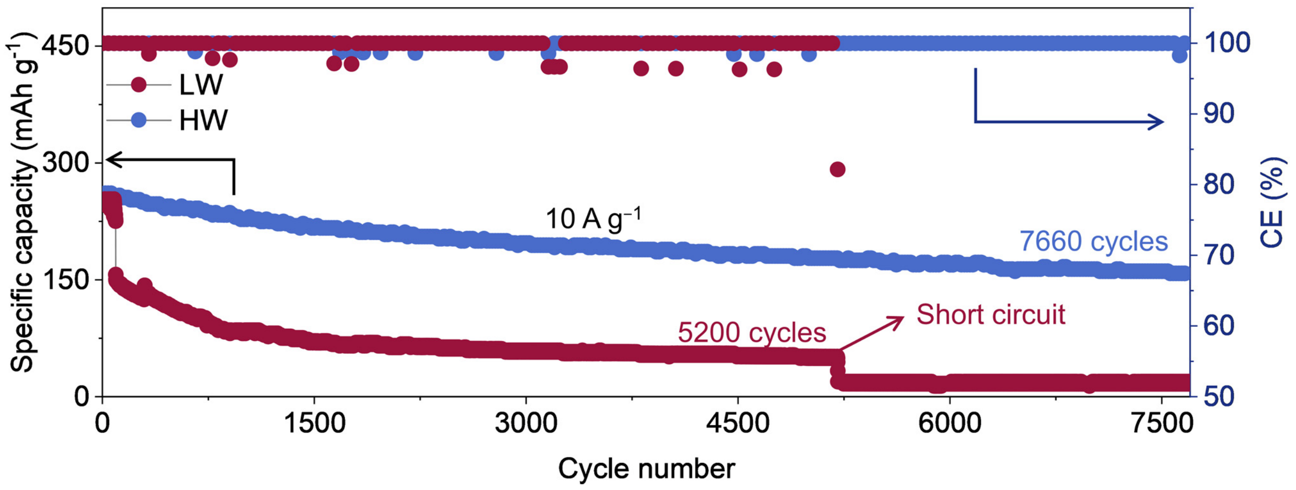

Supplementary Fig. 36 Capacity retention and coulombic efficiency of LW and HW in Zn||NaV_3O_8 coin cells. The electrochemical test is conducted at 25 ± 2 °C.

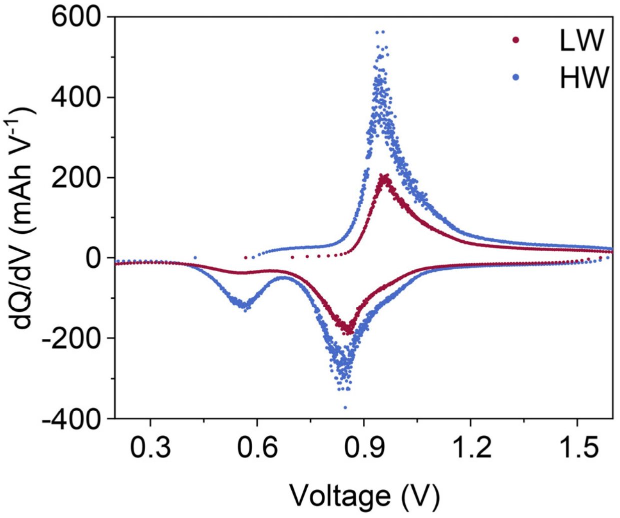

Supplementary Fig. 37 The dQ/dV curves of LW and HW electrolytes in Zn||NaV_3O_8 full cells. The electrochemical test is conducted at 25 ± 2 °C.

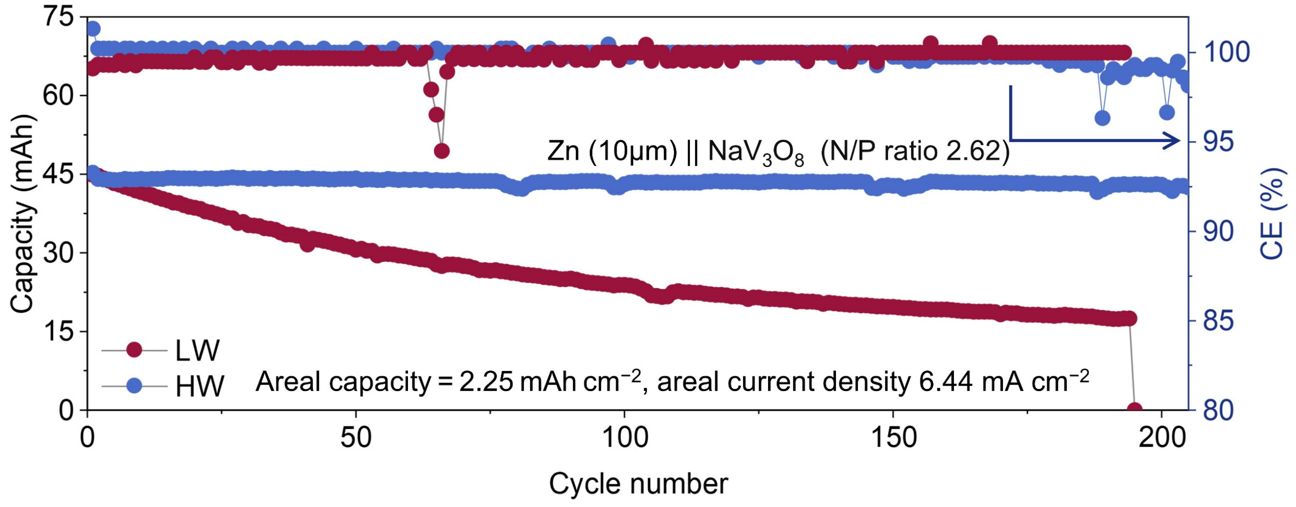

Supplementary Fig. 38 Cycling data graph of LW and HW electrolyte in a Zn(10 μm)||NaV_3O_8 pouch battery. The electrochemical test is conducted at 25 ± 2 ℃.

Supplementary Tables

Supplementary Table 1 Simulated electrolyte parameters in 1M $LiPF_6$ EC solution[7].

Param Name	Value and Unit	Description
Cinit	1000 (mol m^{-3})	Initial concentration
T0	298 (K)	System temperature
i0_ref	350 (A m^{-2})	Exchange current density for reference conditions
phis_anode	0.1 (V)	Anode potential
phis_cathode	−0.1 (V)	Cathode potential
alpha_a	1.5	Symmetry factor
z_Li	1	Charge, species Li^+
z_PF6	−1	Charge, species PF_6^-
D_Li	1^{-10} (m^2 s^{-1})	Diffusivity, species Li^+
D_PF6	1^{-10} (m^2 s^{-1})	Diffusivity, species PF_6^-

Supplementary Table 2 Simulated electrolyte parameters in 1M $ZnSO_4$ H_2O solution[8].

Param Name	Value and Unit	Description
Cinit	1000 (mol m^{-3})	Initial concentration
T0	298 (K)	System temperature
i0_ref	1^{-6} (A m^{-2})	Exchange current density for reference conditions
phis_anode	0.1 (V)	Anode potential
phis_cathode	−0.1 (V)	Cathode potential
alpha_a	1.5	Symmetry factor
z_Zn	2	Charge, species Zn^{2+}
z_SO4	−2	Charge, species SO_4^{-2}
D_Zn	1^{-9} (m^2 s^{-1})	Diffusivity, species Zn^{2+}
D_SO4	1^{-9} (m^2 s^{-1})	Diffusivity, species SO_4^{-2}

Supplementary Table 3 Data obtained from Abbott–Firestone bearing area curve analysis and power spectral density (PSD) analysis.

Surface texture parameters	LW-0min	LW-30min	LW-50min	HW-0min	HW-30min	HW-50min
Sz (maximum height of the surface, μm)	5.24	11.00	14.52	5.42	9.73	10.49
Sa (arithmetical mean height, μm)	0.58	1.48	2.01	0.58	1.24	1.2
Sk (core roughness depth, μm)	1.7	3.0	6.7	1.6	3.4	4.1
Spk (reduced peak height, μm)	1.5	3.1	3.7	1.6	3.4	2.0
Svk (reduced valley depth, μm)	0.5	0.6	0.9	0.4	0.7	1.0
Mr1 (upper material ratio, %)	13.9	19.4	11.8	16.2	17.9	9.7
Mr2 (lower material ratio, %)	91.3	94.5	95.2	93.6	94.2	92.2
λ_{dom} (dominant wavelength, μm)	843.3	780.3	690.6	851.2	780.3	776.9
$A_{PSD,max}$ (maximum PSD amplitude, μm^2)	4.9	7.8	12.3	4.5	6.8	8.1

Supplementary Table 4 Sputtering conditions and calibrated sputtering rates for XPS depth profiling and ToF-SIMS analysis.

Technique	Beam type / energy	Reference sample (matrix)	Reported sputter rate (nm s^{-1})	Citation
XPS depth profiling	Ar^+, 4 keV	Ta_2O_5 (on Si)	0.5 nm·s^{-1}	[9]
	Ar^+, 4 keV	ZnO (insulating film)	≈ 0.167 nm·s^{-1} (≈ 10 nm·min^{-1})	[10]
ToF-SIMS	Ar^+, 500 eV	PVD lithium reference (30 nm Li on Cu)	0.246 ± 0.03 nm·s^{-1} (500 eV Ar^+ @20 nA)	[11]
	Ar_{1500}^+, 5 keV	Li reference (30 nm Li on Cu)	(6.04 ± 0.13) × 10^{-2} nm·s^{-1}	
	Ar_{1500}^+, 10 keV	Li reference (30 nm Li on Cu)	2.69 ± 0.13 nm·s^{-1}	

Supplementary Table 5 Statistical comparison of Depth of Discharge (DOD) testing at low current density (<5mA cm^{-2}) with published work.[12]

Sequence number	Current density (mA cm^{-2})	Lifespan (h)	DOD (%)	Citation
1	2.5	300	20	[13]
2	1	160	80	[14]
3	0.5	200	50	[15]
4	2.5	150	85	[16]
5	1	250	77.7	[17]
6	1	400	50	[18]
7	4	300	85	[19]
8	1	400	90	[20]
9	1	460	56.9	[21]
10	2	280	11.4	[22]
11	2	300	67	[23]
12	3	100	85	[24]
13	2	200	71	[25]
14	2	110	35	[26]
15	1	50	20	[27]
16	2	280	80	[28]

Supplementary Table 6 Statistics related to electrolyte additives and calculation of W_f.

Sequence number	Additives	$\eta_{Additive}$ / η_{Bare} (↓ or ↑)	$W_{f\ Additive}$ / $W_{f\ Bare}$ (↓ or ↑)	Citation
[1]	2 M $ZnSO_4$ + 0.2 M 3,3′-dithiobis-1-propanesulfonic acid disodium salt	0.198 (↓)	1.96 (↑)	29
[2]	1 M $ZnSO_4$ + 0.1 M HTFSI	0.567 (↓)	2.1 (↑)	30
[3]	30 m $ZnCl_2$ + 5 mg vanillin	0.65 (↓)	3.28 (↑)	31
[4]	0.5 M $ZnCl_2$ + 1 M 1-ethyl-3-methyl imidazolium iodide	0.688 (↓)	1.35 (↑)	32
[5]	2 M $ZnSO_4$ + 0.1 M Lithium bis(oxalate)borate	0.716 (↓)	4.04 (↑)	33
[6]	$ZnCl_2 \cdot 3H_2O$ + 0.1g cellulose + 0.1 g PEG	0.72 (↓)	1.60 (↑)	34
[7]	2M $Zn(OTf)_2$ + 50% H_2O + 50% DME	0.750 (↓)	2.38 (↑)	35
[8]	2 M $ZnSO_4$ + 0.5 mg mL^{-1} Fu	0.886 (↓)	1.71 (↑)	36
[9]	2 M $Zn(OTf)_2$ + PVEMA	1	2.82 (↑)	17
[10]	2 M $Zn(ClO_4)_2$ + PMCNA	1.094 (↑)	4.57 (↑)	37
[11]	0.5 M $Zn(TFSI)_2$ 1,3-dioxolane (DOL) / H_2O = 2 / 1	1.25 (↑)	7.10 (↑)	38
[12]	2 M $ZnSO_4$ + 0.5 g L^{-1} saccharin	1.562 (↑)	2.12 (↑)	39
[13]	1 M $ZnSO_4$ + 10 mM Glucose	1.598 (↑)	4.61 (↑)	40
[14]	1 M $Zn(CF_3SO_3)_2$ + n-propyl alcohol	1.660 (↑)	4.42 (↑)	18
[15]	2 M $ZnSO_4$ + 0.2 M PNO	1.667 (↑)	4.70 (↑)	16
[16]	1 m $ZnSO_4$ + 4 m Glycerophosphorylcholine	1.874 (↑)	3.86 (↑)	41
[17]	2 M $ZnSO_4$ + 2.0 wt% Tween-20	2.163 (↑)	4.04 (↑)	42
[18]	2 M $ZnSO_4$ + 0.12 M pyridine	3.3 (↑)	1.65 (↑)	43
[19]	3 M $ZnSO_4$ + 10 mM Cyclodextrins	0.64 (↓)	Data scarcity	44
[20]	1 M $ZnSO_4$ + 5 mM D-mannose and sodium lignosulfonate	0.67 (↓)	Data scarcity	45
[21]	2 M $ZnSO_4$ + Trehalose	1	Data scarcity	46
[22]	2 M $ZnSO_4$ + DMS + LS	1.1 (↑)	Data scarcity	47
[23]	$ZnSO_4$ + 50 mM methylurea	2.98 (↑)	Data scarcity	48

Supplementary Table 7 Equivalent circuit model and residual error between experimental and fitted data.

Figure	Corresponding data	Root-mean-square error (RMSE)	Solution resistance (R_s)	Charge transfer resistance (R_{ct})	CPE exponent (p_CPE)	CPE coefficient (T_CPE)
**Supplementary Fig. S18 b** LW electrolyte	2.0 mA cm^{-2}	5.71	2.67	406.94	0.407	84.0E-05
	1.5 mA cm^{-2}	7.12	3.34	444.51	0.534	31.4E-05
	1.0 mA cm^{-2}	5.56	1.20	433.40	0.508	87.3E-05
	0.5 mA cm^{-2}	6.44	1.02	597.66	0.387	1.35E-03
	0.2 mA cm^{-2}	7.76	1.32	689.12	0.510	62.6E-05
**Supplementary Fig. S18 b** HW electrolyte	2.0 mA cm^{-2}	2.19	5.73	101.61	0.744	10.8E-05
	1.5 mA cm^{-2}	2.25	0.88	116.19	0.778	6.8E-05
	1.0 mA cm^{-2}	2.16	2.71	124.74	0.758	6.7E-05
	0.5 mA cm^{-2}	2.49	3.12	143.39	0.758	5.9E-05
	0.2 mA cm^{-2}	5.14	2.64	150.71	0.768	12.1E-05
**Supplementary Fig. S19 c**	LW	2.30	0.15	158.39	0.631	12.0E-05
	HW	2.34	1.22	146.27	0.597	23.4E-05
**Supplementary Fig. S32 b**	HW-100 h	4.99	3.75	447.86	0.861	2.2E-05
	HW-200 h	6.74	5.08	673.15	0.842	2.5E-05
	LW-100 h	8.39	4.75	865.41	0.729	3.5E-05
	LW-200 h	8.30	3.74	1104.95	0.707	3.8E-05

Supplementary References

1 Matthews, D. The Stern-Geary and related methods for determining corrosion rates. *Aust. J. Chem.* **28**, 243-251 (1975).

2 Stern, M. & Geary, A. L. Electrochemical Polarization: I . A Theoretical Analysis of the Shape of Polarization Curves. *J. Electrochem. Soc.* **104**, 56 (1957).

3 Schalenbach, M., Durmus, Y. E., Tempel, H., Kungl, H. *et al.* Double layer capacitances analysed with impedance spectroscopy and cyclic voltammetry: validity and limits of the constant phase element parameterization. *PCCP* **23**, 21097-21105 (2021).

4 Scully, J. R. Polarization Resistance Method for Determination of Instantaneous Corrosion Rates. *Corrosion* **56**, 199-218 (2000).

5 Lazanas, A. C. & Prodromidis, M. I. Electrochemical Impedance Spectroscopy-A Tutorial. *ACS Meas Sci Au* **3**, 162-193 (2023).

6 Scharifker, B. & Hills, G. Theoretical and experimental studies of multiple nucleation. *Electrochim. Acta* **28**, 879-889 (1983).

7 Shi, F., Pei, A., Vailionis, A., Xie, J. *et al.* Strong texturing of lithium metal in batteries. *Proc. Natl. Acad. Sci.* **114**, 12138-12143 (2017).

8 Xie, X., Liang, S., Gao, J., Guo, S. *et al.* Manipulating the ion-transfer kinetics and interface stability for high-performance zinc metal anodes. *Energy Environ. Sci.* **13**, 503-510 (2020).

9 Baer, D. R., Engelhard, M. H., Lea, A. S., Nachimuthu, P. *et al.* Comparison of the sputter rates of oxide films relative to the sputter rate of SiO2. *J. Vac. Sci. Technol., A* **28**, 1060-1072 (2010).

10 Schuler, L. P., Alkaisi, M. M., Miller, P., Reeves, R. J. *et al.* Comparison of DC and RF Sputtered Zinc Oxide Films with Post-Annealing and Dry Etching and Effect on Crystal Composition. *Jpn. J. Appl. Phys.* **44**, 7555 (2005).

11 Mense, M., Bela, M. M., Kühn, S. P., Cekic-Laskovic, I. *et al.* ToF-SIMS sputter depth profiling of interphases and coatings on lithium metal surfaces. *Commun. Chem.* **8**, 31 (2025).

12 Wang, Z., Chen, J., Yang, Z., Liu, Y. *et al.* Zinc utilization rate in aqueous batteries: regulation of interfacial thermodynamics and kinetics. *Natl. Sci. Open*, 20250044.

13 Ma, L., Pollard, T. P., Zhang, Y., Schroeder, M. A. *et al.* Functionalized Phosphonium Cations Enable Zinc Metal Reversibility in Aqueous Electrolytes. *Angew. Chem. Int. Ed.* **60**, 12438-12445 (2021).

14 Zhao, R., Wang, H., Du, H., Yang, Y. *et al.* Lanthanum nitrate as aqueous electrolyte additive for favourable zinc metal electrodeposition. *Nat. Commun.* **13**, 3252 (2022).

15 Han, D., Cui, C., Zhang, K., Wang, Z. *et al.* A non-flammable hydrous organic electrolyte for sustainable zinc batteries. *Nat. Sustain.* **5**, 205-213 (2022).

16 Li, Z., Wang, Z., Sun, W., Ma, Y. *et al.* Regulating Interface Engineering by Helmholtz Plane Reconstructed Achieves Highly Reversible Zinc Metal Anodes. *Adv. Mater.* **37**, 2420489 (2025).

17 Wu, C., Pan, Y., Jiao, Y. & Wu, P. α-Methyl Group Reinforced Amphiphilic Poly(Ionic Liquid) Additive for High-Performance Zinc–Iodine Batteries. *Angew. Chem. Int. Ed.* **64**, e202423326 (2025).

18 Zhu, Y., Li, H., Sun, X., Chen, A. *et al.* Minimizing Zn Loss Through Dual Regulation for Reversible Zinc Anode Beyond 90% Utilization Ratio. *Small* **21**, 2411986 (2025).

19 Zhu, M., Hu, J., Lu, Q., Dong, H. *et al.* A Patternable and In Situ Formed Polymeric Zinc

Blanket for a Reversible Zinc Anode in a Skin-Mountable Microbattery. *Adv. Mater.* **33**, 2007497 (2021).

20 Lee, D., Kim, H.-I., Kim, W.-Y., Cho, S.-K. *et al.* Water-Repellent Ionic Liquid Skinny Gels Customized for Aqueous Zn-Ion Battery Anodes. *Adv. Funct. Mater.* **31**, 2103850 (2021).

21 Cui, Y., Zhao, Q., Wu, X., Chen, X. *et al.* An Interface-Bridged Organic–Inorganic Layer that Suppresses Dendrite Formation and Side Reactions for Ultra-Long-Life Aqueous Zinc Metal Anodes. *Angew. Chem. Int. Ed.* **59**, 16594-16601 (2020).

22 Zhang, Q., Luan, J., Tang, Y., Ji, X. *et al.* Interfacial Design of Dendrite-Free Zinc Anodes for Aqueous Zinc-Ion Batteries. *Angew. Chem. Int. Ed.* **59**, 13180-13191 (2020).

23 Duan, F., Yin, X., Ba, J., Li, J. *et al.* A Hydrophobic and Zincophilic Interfacial Nanofilm as a Protective Layer for Stable Zn Anodes. *Adv. Funct. Mater.* **34**, 2310342 (2024).

24 Gao, Y., Yang, N., Bu, F., Cao, Q. *et al.* Double-sided engineering for space-confined reversible Zn anodes. *Energy Environ. Sci.* **17**, 1894-1903 (2024).

25 Guo, W., Cong, Z., Guo, Z., Chang, C. *et al.* Dendrite-free Zn anode with dual channel 3D porous frameworks for rechargeable Zn batteries. *Energy Stor. Mater.* **30**, 104-112 (2020).

26 Zeng, Y., Zhang, X., Qin, R., Liu, X. *et al.* Dendrite-Free Zinc Deposition Induced by Multifunctional CNT Frameworks for Stable Flexible Zn-Ion Batteries. *Adv. Mater.* **31**, 1903675 (2019).

27 Wang, Z., Huang, J., Guo, Z., Dong, X. *et al.* A Metal-Organic Framework Host for Highly Reversible Dendrite-free Zinc Metal Anodes. *Joule* **3**, 1289-1300 (2019).

28 Zhang, Q., Luan, J., Fu, L., Wu, S. *et al.* The Three-Dimensional Dendrite-Free Zinc Anode on a Copper Mesh with a Zinc-Oriented Polyacrylamide Electrolyte Additive. *Angew. Chem. Int. Ed.* **58**, 15841-15847 (2019).

29 Li, T., Naveed, A., Zheng, J., Chen, B. *et al.* Engineering Aqueous Electrolytes with Vicinal S-based Organic Additives for Highly Reversible Zinc-Ion Batteries. *Angew. Chem. Int. Ed.* **64**, e202424095 (2025).

30 Nian, Q., Luo, X., Ruan, D., Li, Y. *et al.* Highly reversible zinc metal anode enabled by strong Brønsted acid and hydrophobic interfacial chemistry. *Nat. Commun.* **15**, 4303 (2024).

31 Hoang, D., Li, Y., Jung, M. S., Sandstrom, S. K. *et al.* Vanillin: An Effective Additive to Improve the Longevity of Zn Metal Anode in a 30 m ZnCl2 Electrolyte. *Adv. Energy Mater.* **13**, 2301712 (2023).

32 Behera, A., Deb, D. & Bhattacharyya, A. J. Employing an EMImI ionic liquid additive in Zn-salt solutions for reversible Zn stripping and plating in an advanced aqueous Zn-ion battery. *J. Mater. Chem. A* **13**, 33784-33797 (2025).

33 Zhang, Z., Zhang, Y., Ye, M., Wen, Z. *et al.* Lithium Bis(oxalate)borate Additive for Self-repairing Zincophilic Solid Electrolyte Interphases towards Ultrahigh-rate and Ultra-stable Zinc Anodes. *Angew. Chem. Int. Ed.* **62**, e202311032 (2023).

34 Yang, L., Fu, Y., Liu, H., Nie, Q. *et al.* Investigating the Zinc Deposition Behavior in Aqueous Zinc-Ion Batteries with PEG/Cellulose/ZnCl2 Water-In-Salt Electrolytes via a Homemade Visualized Three-Electrode Tubular Cell. *ACS Sustainable Chem. Eng.* **11**, 10311-10323 (2023).

35 Zhou, K., Li, Z., Qiu, X., Yu, Z. *et al.* Boosting Zn Anode Utilization by Trace Iodine Ions in Organic-Water Hybrid Electrolytes through Formation of Anion-rich Adsorbing Layers. *Angew. Chem. Int. Ed.* **62**, e202309594 (2023).

36 Huang, J., Zhong, Y., Fu, H., Zhao, Y. *et al.* Interfacial Biomacromolecular Engineering Toward Stable Ah-Level Aqueous Zinc Batteries. *Adv. Mater.* **36**, 2406257 (2024).
37 Feng, D., Jiao, Y. & Wu, P. Guiding Zn Uniform Deposition with Polymer Additives for Long-lasting and Highly Utilized Zn Metal Anodes. *Angew. Chem. Int. Ed.* **62**, e202314456 (2023).
38 Liu, Z., Xi, M., Sheng, R., Huang, Y. *et al.* Zn(TFSI)2-Mediated Ring-Opening Polymerization for Electrolyte Engineering Toward Stable Aqueous Zinc Metal Batteries. *Nano-Micro Lett.* **17**, 120 (2025).
39 Huang, C., Zhao, X., Liu, S., Hao, Y. *et al.* Stabilizing Zinc Anodes by Regulating the Electrical Double Layer with Saccharin Anions. *Adv. Mater.* **33**, 2100445 (2021).
40 Sun, P., Ma, L., Zhou, W. H., Qiu, M. J. *et al.* Simultaneous Regulation on Solvation Shell and Electrode Interface for Dendrite-Free Zn Ion Batteries Achieved by a Low-Cost Glucose Additive. *Angew. Chem. Int. Ed.* **60**, 18247-18255 (2021).
41 Lyu, H., Zhao, S., Liao, C., Li, G. *et al.* Electric Double Layer Oriented Eutectic Additive Design toward Stable Zn Anodes with a High Depth of Discharge. *Adv. Mater.* **36**, 2400976 (2024).
42 Deng, Q., You, S., Min, W., Xu, Y. *et al.* Polymer Molecules Adsorption-Induced Zincophilic-Hydrophobic Protective Layer Enables Highly Stable Zn Metal Anodes. *Adv. Mater.* **36**, 2312924 (2024).
43 Luo, J., Xu, L., Zhou, Y., Yan, T. *et al.* Regulating the Inner Helmholtz Plane with a High Donor Additive for Efficient Anode Reversibility in Aqueous Zn-Ion Batteries. *Angew. Chem. Int. Ed.* **62**, e202302302 (2023).
44 Zhao, K., Fan, G., Liu, J., Liu, F. *et al.* Boosting the Kinetics and Stability of Zn Anodes in Aqueous Electrolytes with Supramolecular Cyclodextrin Additives. *J. Am. Chem. Soc.* **144**, 11129-11137 (2022).
45 Li, H., Ren, Y., Zhu, Y., Tian, J. *et al.* A Bio-Inspired Trehalose Additive for Reversible Zinc Anodes with Improved Stability and Kinetics. *Angew. Chem. Int. Ed.* **62**, e202310143 (2023).
46 Wu, F., Zhang, J., Ma, L., Ruan, P. *et al.* Directing Zn Growth with Biased Adsorption of Straight-chain Molecules for Superior Zn Anode Stability. *Angew. Chem. Int. Ed.* **64**, e202421787 (2025).
47 Chen, H.-B., Meng, H., Zhang, T.-R., Ran, Q. *et al.* Dynamic Molecular Interphases Regulated by Trace Dual Electrolyte Additives for Ultralong-Lifespan and Dendrite-Free Zinc Metal Anode. *Angew. Chem. Int. Ed.* **63**, e202402327 (2024).
48 Wang, M., Ma, J., Meng, Y., Tong, P. *et al.* In situ formation of solid electrolyte interphase facilitates anode-free aqueous zinc battery. *eScience* **5**, 100397 (2025).
