# Supplementary material for: Revisiting Zn-specific nucleation via a dimensionless factor to quantify interfacial electrochemistry of aqueous batteries
Source: Nat Commun. 2026 Jun 2;17:7067. doi: 10.1038/s41467-026-73953-w (PMC13392247; doi:10.1038/s41467-026-73953-w)
Supplement: Supplementary file 2 — Description of Additional Supplementary Files [file 41467_2026_73953_MOESM2_ESM.pdf]

Description of Additional Supplementary Files

File Name: Supplementary Data 1
Description: Structural models of the Zn (001), (100), and (101) surfaces.

File Name: Supplementary Data 2
Description: Adsorption configurations of the ammonium tartrate molecule on the Zn (001), (100), and (101) surfaces.

File Name: Supplementary Data 3
Description: Adsorption configurations of the H_2O molecule on the Zn (001), (100), and (101) surfaces.
