# Supplementary material for: Revisiting Zn-specific nucleation via a dimensionless factor to quantify interfacial electrochemistry of aqueous batteries
Source: Nat Commun. 2026 Jun 2;17:7067. doi: 10.1038/s41467-026-73953-w (PMC13392247; doi:10.1038/s41467-026-73953-w)
Supplement: Supplementary file 4 — Transparent Peer Review file [file 41467_2026_73953_MOESM4_ESM.pdf]

nature portfolio

Peer Review File

Revisiting Zn-specific nucleation via a dimensionless factor to quantify interfacial electrochemistry of aqueous batteries

Corresponding Author: Professor Dongliang Chao

This file contains all reviewer reports in order by version, followed by all author rebuttals in order by version.

Version 0:

Reviewer comments:

Reviewer #1

(Remarks to the Author)
This manuscript derives distinct nucleation models for lithium and zinc metals based on classical nucleation theory, establishes a nucleation framework specifically for hexagonal close-packed zinc, and validates the theoretical predictions through experimental observations. In addition, the authors introduce a dimensionless descriptor to quantitatively characterize interfacial electrodeposition behavior, elegantly coupling nucleation driving force and kinetic resistance into a unified framework. This descriptor provides a practical screening criterion for achieving efficient zinc deposition on aqueous Zn-ion battery anodes and is further validated against previously reported studies. Guided by this descriptor, the authors design an optimized electrolyte that enables highly uniform zinc electrodeposition and excellent full-cell performance. Overall, the study is of significant interest. I recommend publication in Nature Communications after the authors address the following comments.

1. The manuscript involves substantial mathematical equations when comparing different nucleation models. It would benefit from a more explicit and intuitive definition of the nucleation evolution process itself, rather than describing it primarily in terms of rate changes. In addition, a discussion of the subsequent growth behavior associated with different nucleation regimes would help provide a more complete physical picture.
2. In the statistical analysis of deposition morphology under different overpotentials and surface energy conditions, the linear fitting presented in Figure 2e is based on only three data points. The statistical representativeness of this analysis could be improved. Increasing the number of data points or experimental conditions is recommended to strengthen the reliability of the observed trend.
3. The manuscript derives a theoretical relationship between overpotential and current density under symmetric-cell conditions (Eq. 8). It is recommended that this relationship be quantitatively compared with experimental data, which would provide strong additional validation of the proposed model. Such results could be included in the main text or as supplementary information.
4. For clarity, the authors should explicitly state that all physical quantities involved in the dimensionless descriptor are defined on a per-unit-area basis. This clarification would help avoid potential ambiguity in interpretation and application.
5. In Figure 3f, the authors simulate the post-deposition surface potential distribution using an electrostatic model. While the qualitative trends appear reasonable, a more physically consistent approach would be to simulate the surface potential under an imposed current-density boundary condition at the electrode surface, which would more directly reflect realistic electrodeposition conditions.
6. In Figure 4, the designed electrolyte exhibits excellent corrosion resistance and suppression of side reactions, as claimed in the manuscript. A more detailed discussion of the underlying mechanisms at the electrolyte-Zn anode interface, such as solvation structure regulation, interfacial reaction pathways, or electric-field modulation, would further strengthen the interpretation of these results.

Reviewer #2

(Remarks to the Author)
The manuscript titled "Quantifying Interfacial Electrochemistry in Aqueous Batteries: A Dimensionless Factor via Revisiting Zn-Specific Nucleation Modeling" introduces promising concept by linking a new electrochemical descriptor to Zn electrodeposition stability. However, it currently over-relies on simplified models and limited experimental validation. The

authors must deepen the theoretical framework, rigorously validate the dimensionless factor Wf both experimentally and via simulations, incorporate advanced operando and microstructural analyses, and address practical considerations before drawing broad, mechanistically grounded conclusions. After these minor revisions, the work will meet the high standards for publication.
1. This work proposes dimensionless Wf parameter as a “universal” descriptor quantitatively coupling electrodeposition driving force and interfacial resistance. However, the validation of Wf relies primarily on some literature-derived data points and limited in-situ measurements, which are insufficient for establishing “universal” applicability. There is a notable absence of systematic, controlled experimental datasets spanning a broad parameter space (e.g., “different” electrolyte chemistries, concentrations, additives, and operational conditions) that explicitly test the correlation between computed Wf values and actual nucleation, morphology, and cycle life.
The authors should incorporate rigorous, quantitative validation through systematically controlled electrochemical measurements, e.g., electrochemical impedance spectroscopy over a wide frequency range, in conjunction with microscopy-characterized nucleation behavior, to directly correlate experimental Wf calculations with “microstructural evolution”.
2. The derivation of Wf appears to rest on a simplified model approximation, e.g., the ratio of polarization potential to the product of corrosion current and polarization resistance (Eq. 9). This oversimplification neglects possible nonlinearities inherent in electrochemical processes, such as the influence of double-layer capacitance, non-ideal solution behavior, surface heterogeneities, and complex parasitic reactions. There is scant theoretical underpinning or derivation from first principles justifying the form of Wf, particularly how the interplay of these parameters universally governs the nucleation mode and stability across different systems. So, it would be better to consider the fundamental thermodynamic and kinetic principles (including non-ideal solution effects) while deriving the Wf, to establish its theoretical generality.
3. While the authors extend CNT to account for Zn’s HCP crystal structure by modeling anisotropic nucleation, they do not sufficiently demonstrate that their modified nucleation energy barrier and critical nucleus size calculations incorporate all relevant atomic-scale energetic contributions. The reliance on classical expressions neglects the non-classical, non-spherical, or mesoscopic nucleation phenomena such as 2D nucleation, defect-mediated nucleation, or kinetic pathways involving pre-activation clusters. The implications of such pathways on deposition morphology, dendrite formation, and cycle life are underrepresented. It would be great to see if the authors could extend the theoretical modelling with molecular dynamics simulations to explore these circumstances.

4. Although SEM images and in-situ optical microscopy suggest improved morphological stability in high-Wf electrolytes, these characterizations are inadequate for conclusively linking the Wf value with nucleation and growth phenomena at the atomic and mesoscale. The deposition process is inherently stochastic, and factors such as grain orientation, defect density, and local current distribution critically influence dendrite suppression. The study overlooks advanced in-situ or operando techniques capable of capturing real-time, three-dimensional evolution of nucleation sites and dendritic growth modes. Without such data, the assertion that Wf predicts, and guides suppression of dendrite formation remains phenomenological. The authors may consider the operando electron microscopy (e.g., in-situ TEM or synchrotron-based X-ray tomography) during cycling, coupled with microstructural analysis, to directly observe nucleation sites, growth fronts, and dendritic features. It would be more impressive if the authors can quantify the correlation between computed Wf and microstructural parameters such as nucleation density, tip growth rates, and defect distribution.
4. The chemical analyses (XRD, XPS, ToF-SIMS) demonstrate the suppression of zinc hydroxide sulfate layers in high-Wf electrolytes, suggesting reduced parasitic reactions. However, the mechanistic link between Wf, interfacial chemistry, and suppression of side reactions is underdeveloped. In addition, the role of additive adsorption, solvation structure modifications, and double-layer restructuring are acknowledged but lack quantitative mechanistic insight. Notably, the long-term effects of local pH shifts, the evolution of interphases, and potential failure modes are not thoroughly characterized. The authors might conduct in-depth interfacial studies under operando conditions, including in-situ X-ray absorption spectroscopy, to elucidate real-time interphase formation, chemical evolution, and their direct relationship with Wf. Furthermore, it would be better to study the influence of additive chemistries on interfacial kinetics and composition at multiple cycling stages.
5. The in situ 3D confocal microscopy (Fig. 3e) is a highlight, but the analysis is qualitative. Quantitative metrics such as surface fractal dimension or local curvature analysis would strengthen the conclusion regarding deposition uniformity.

Reviewer #3

(Remarks to the Author)
This manuscript investigates, from the perspective of interfacial electrochemistry, the nucleation and growth behavior of zinc during electrodeposition in aqueous zinc-ion batteries. By combining theoretical analysis with a variety of electrochemical measurements and structural characterizations, the authors establish correlations between interfacial kinetic parameters and deposition stability. The descriptors proposed in the manuscript have a clear physical basis and offer valuable insight and guidance for understanding zinc-anode failure mechanisms and for designing electrolyte/interface regulation strategies. The work demonstrates high novelty with complete supports; the experimental and analytical results can support the authors' main conclusions. I think the manuscript is suitable for publication in Nature Communications and recommend acceptance after minor revision.

a) The manuscript primarily emphasizes interfacial electrochemical regulation (e.g., additive adsorption, reduction of γ, changes in Rct, and suppression of corrosion current), while bulk electrolyte properties such as ionic conductivity and diffusion coefficients are not adequately addressed. The authors should include basic characterizations of the electrolyte bulk phase and briefly discuss how these properties may influence the observed interfacial behavior.
b) Some parameters require clarification. In particular, Table S2 lists the system temperature T0 = 200 K, which is inconsistent with room-temperature experiments. Please confirm the simulation temperature and indicate whether this is a

typographical error. The authors should also provide basic modeling details, including boundary and initial conditions, mesh size and convergence checks, and whether Butler-Volmer kinetics are coupled, to ensure the simulations are physically meaningful and reproducible.
c) The Supplementary Information indicates that XPS depth profiling was performed using 4 keV Ar+ sputtering and that ToF-SIMS used 2 keV Ar+. The authors should provide sputtering-rate calibration data (in nm s−1) based on a reference or standard sample, and briefly discuss possible sputtering-induced effects such as chemical reduction or elemental migration, particularly for zinc valence states and oxygen-containing species, so that readers can assess the quantitative reliability of the depth profiles.
d) On pages 3 and 5, the subsection titles “Classical nucleation theory for Zn metal” and “Validation of the Zn classical nucleation theory” would benefit from revision because they do not clearly highlight the specific focus of each section and may inadvertently discourage careful reading. The authors are encouraged to revise these titles better to reflect the key emphasis of the respective sections.
e) In Figures 4c and 4d, the XPS data treatment appears inappropriate: the S-related peaks should be deconvoluted using a doublet-fitting approach. The authors should reprocess the XPS data and revise the analysis accordingly.

Version 1:

Reviewer comments:

Reviewer #1

(Remarks to the Author)
The authors have satisfactorily addressed the previous comments with additional experiments and responses. I recommend the manuscript for acceptance.

Reviewer #2

(Remarks to the Author)
The authors have answered all my questions.

Reviewer #3

(Remarks to the Author)
The authors have addressed my comments, and I recommend its publication in Nature Communications.

Open Access This Peer Review File is licensed under a Creative Commons Attribution 4.0 International License, which permits use, sharing, adaptation, distribution and reproduction in any medium or format, as long as you give appropriate credit to the original author(s) and the source, provide a link to the Creative Commons license, and indicate if changes were made.
In cases where reviewers are anonymous, credit should be given to 'Anonymous Referee' and the source.
The images or other third party material in this Peer Review File are included in the article’s Creative Commons license,

unless indicated otherwise in a credit line to the material. If material is not included in the article's Creative Commons license and your intended use is not permitted by statutory regulation or exceeds the permitted use, you will need to obtain permission directly from the copyright holder.
To view a copy of this license, visit https://creativecommons.org/licenses/by/4.0/

RESPONSE TO REVIEWS

We sincerely thank the Reviewers for the valuable and constructive comments. We are encouraged that all 3 Reviewers suggested publication with "minor revisions". Now, we have carefully considered and addressed all of the Reviewers' points to the best of our ability. For ease of reference, the Reviewers' comments and suggestions are reproduced in **blue**. Our responses are provided in **black**. Revisions in the manuscript (**R-MS**) and the Supporting Information (**R-SI**) are presented in ***'italics'***. All modified content has been **highlighted in yellow** in both **R-MS** and the **R-SI**.

Response to Reviewer #1

Remarks to the Author

This manuscript derives distinct nucleation models for lithium and zinc metals based on classical nucleation theory, establishes a nucleation framework specifically for hexagonal close-packed zinc, and validates the theoretical predictions through experimental observations. In addition, the authors introduce a dimensionless descriptor to quantitatively characterize interfacial electrodeposition behavior, elegantly coupling nucleation driving force and kinetic resistance into a unified framework. This descriptor provides a practical screening criterion for achieving efficient zinc deposition on aqueous Zn-ion battery anodes and is further validated against previously reported studies. Guided by this descriptor, the authors design an optimized electrolyte that enables highly uniform zinc electrodeposition and excellent full-cell performance. Overall, the study is of significant interest. I recommend publication in *Nature Communications* after the authors address the following comments.

Response

We sincerely thank Reviewer #1 for the insightful and constructive feedback, which has proved invaluable in enhancing the quality of our manuscript. All comments have been carefully considered and systematically addressed in the revised manuscript and Supporting Information. We herewith submit the **R-MS** and **R-SI** for publication in *Nature Communications*, together with a point-by-point response to the comments.

Original comment 1-1

The manuscript involves substantial mathematical equations when comparing different nucleation models. It would benefit from a more explicit and intuitive definition of the nucleation evolution process itself, rather than describing it primarily in terms of rate changes. In addition, a discussion of the subsequent growth behavior associated with different nucleation regimes would help provide a more complete physical picture.

Response

We appreciate the Reviewer's constructive comments, which have been instrumental in enhancing the quality of our manuscript. To address the Reviewer's suggestion, we have incorporated a new schematic (**Supplementary Fig. 6**) that elucidates the nucleation evolution process and its subsequent growth behavior. Specifically, the schematic provides a comparative analysis of nucleus size and surface density for spherical versus HCP nucleation across various R ratios. Furthermore, it provides

a direct numerical comparison of the critical free energy difference, ΔG^*, and the critical nuclei density, N^*, for both models, using graphical representations to facilitate a clearer interpretation of the underlying mechanisms.

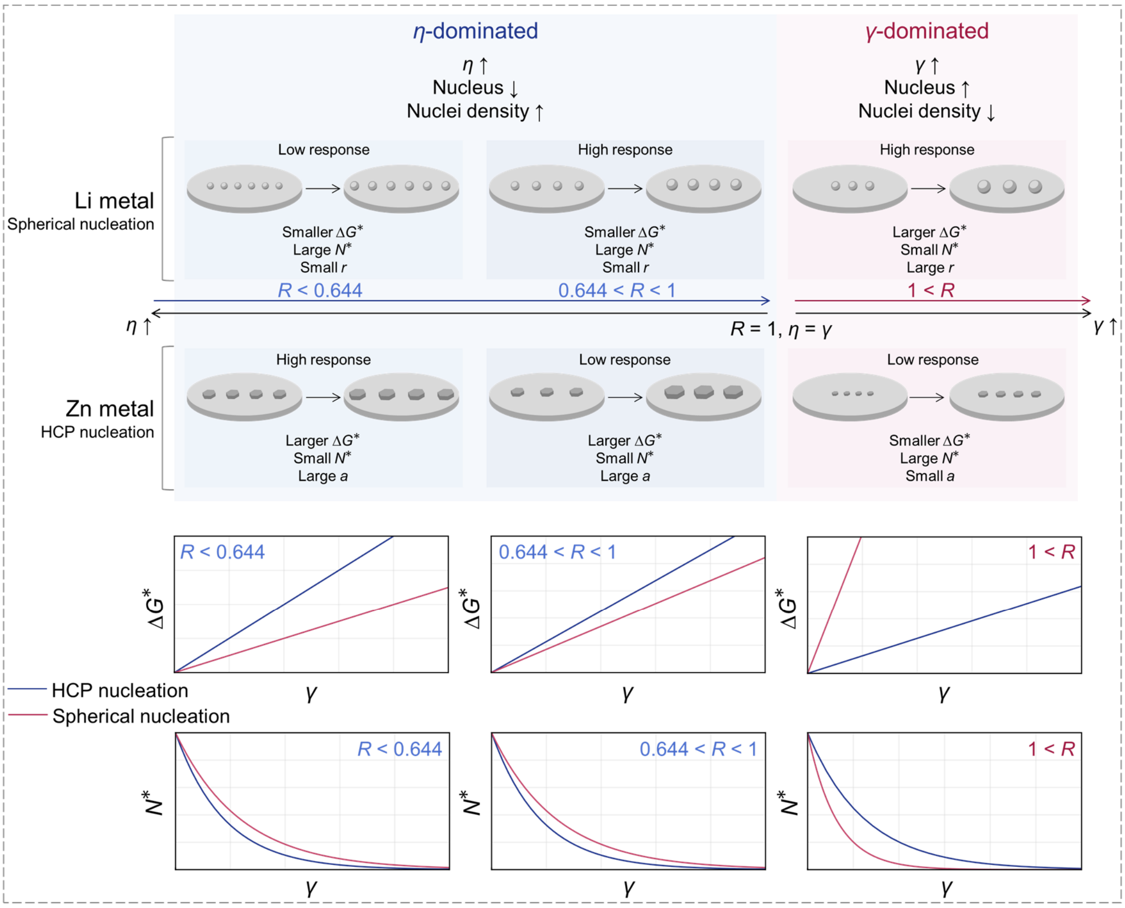

Supplementary Fig. 6 Schematic illustration of the nucleation and growth processes for spherical nucleation and HCP nucleation at different R values, together with the numerical relationships between the critical free-energy difference (ΔG^*) and the critical nuclei density (N^*).

In response, the relevant figure has been added as **Supplementary Fig. 6** in **R-SI** (**page 17**). Moreover, the additional descriptions have also been included in **R-MS** (**page 4**) and **R-SI** (**page 17**): *'A positive real solution $R = 0.644$ is obtained under the condition $|\nabla A| / |\nabla B| = 1$. This value identifies a clear threshold for the relative behavior of the two nucleation modes. For $R < 0.644$, HCP nucleation exhibits a stronger dependence on η, as both the ΔG^* and N^* evolve rapidly with increasing η. Furthermore, N^* exhibits an exponential decay, which indicates a precipitous decrease (Fig. 1d). In the intermediate range $0.644 < R < 1$, the spherical nucleation mode varies more quickly than the HCP mode (Fig. 1d). When the nucleation process is dominated by γ ($R \geq 1$), this trend continues and the rates of change of ΔG^* and N^* in the spherical nucleus accelerate significantly with increasing R.'* in ***R-MS (page 4)***

'The stability of metal nucleation and subsequent growth is governed by the critical nucleus size (a or r) and the N^*. Schematic illustrations of these specific variations are summarized in Supplementary

Fig. 6. In particular, the HCP nucleation yields a higher N^* when $R < 1$, whereas the N^* becomes lower for $R \geq 1$. Overall, the divergence between the two nucleation modes results in distinct patterns of variation in ΔG^* and N^*. Consequently, this analysis provides a theoretical rationale for applying the HCP nucleation model to Zn electrodeposition. These findings demonstrate that γ and η jointly govern nucleation thermodynamics and determine the spatial uniformity of Zn deposition.' in ***R-MS (page 4)***

*'**Supplementary Fig. 6** indicates that the stability of metal nucleation and subsequent growth is governed by the critical nucleus size (a or r) and the nucleation density N^*. For both spherical and HCP nucleation, a higher nucleation density, which corresponds to a larger overpoten 'tial or a lower surface energy, provides more available nucleation sites and promotes more uniform deposition. In addition, a smaller critical nucleus radius reduces the likelihood that large dendritic structures will form and penetrate the separator. Therefore, under cross-flow charge–discharge conditions and assuming ideal planar nucleation, larger applied currents (Equation (8)) tend to favor a more uniform electrodeposition interface.' in* ***R-SI (page 17)***

Original comment 1-2

In the statistical analysis of deposition morphology under different overpotentials and surface energy conditions, the linear fitting presented in Figure 2e is based on only three data points. The statistical representativeness of this analysis could be improved. Increasing the number of data points or experimental conditions is recommended to strengthen the reliability of the observed trend.

Response

We sincerely thank the Reviewer for this valuable suggestion. In response, to enhance the reliability of the statistical analysis and the robustness of the model validation, we have conducted additional experiments to characterize deposition morphologies at current densities of 0.5 mA cm^{-2} and 1.5 mA cm^{-2}, maintaining a constant areal capacity of 0.2 mAh cm^{-2}. The grain morphologies were characterized by scanning electron microscopy (Hitachi SU8600) and are shown in **Fig. 2c, and Supplementary Figs. 12, 14**. The corresponding statistical correlations are summarized in **Fig. 2d**. Furthermore, the linear relationship between η^{-1} and the nuclei length (**Fig. 2e**) remains valid with the expanded dataset, confirming the consistency of the observed trend. Additionally, the nuclei length distributions for these conditions are detailed in **Supplementary Fig. 16**.

In response to this comment, the corresponding figures have been added to the **R-MS**, with **Fig. 2** on **page 7** and **Supplementary Figs. 12**, **14**, and **16** included in the **R-SI** (**pages 23**, **25**, and **27**). In addition, an expanded description has been incorporated into the **R-MS** (**page 6**):

'Subsequently, galvanostatic deposition of 0.2 mAh cm^{-2} Zn is performed on Zn foil (Supplementary Fig. 10) at current densities of 0.2, 0.5, 1.0, 1.5, and 2.0 mA cm^{-2}. Scanning electron microscopy (SEM) imaging (Fig. 2c, Supplementary Figs. 11-15) reveals HCP nucleation morphology, and statistical analysis (Fig. 2d) demonstrates that increasing current density elevates the nucleation overpotential (BA: 67.5 → 169.1 mV, AD: 35.0 → 120.0 mV) while reducing the nuclei length (BA: 560.0 → 251.4 nm, AD: 359.9 → 171.8 nm).' in ***R-MS (page 6)***

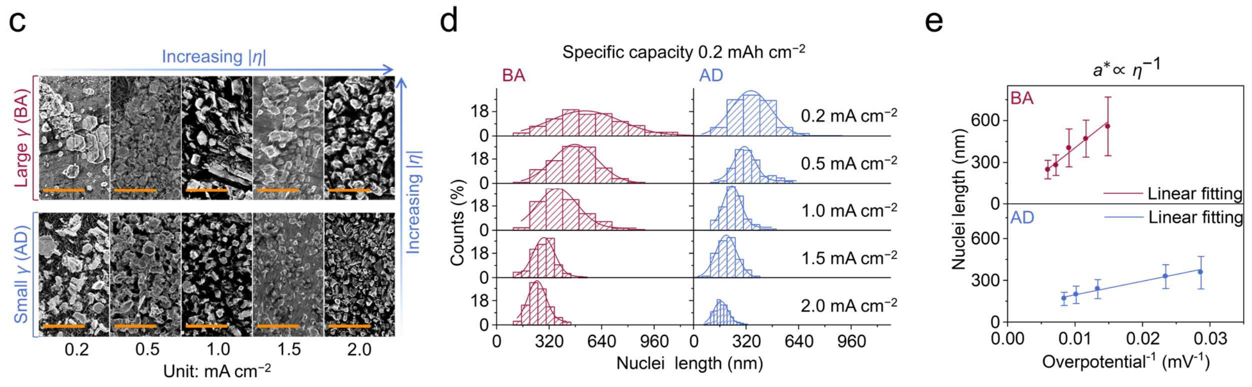

Figure 2. **Experimental validation of the Zn-HCP nucleation theory. c** Scanning electron microscopy (SEM) images of Zn deposition morphologies under varying η and γ (scale bar: 2 μm). **d** Statistical correlation between nuclei length and polarization, where BA is shown in red and AD in blue. **e** Linear fitting of η^{-1} and the nuclei length.

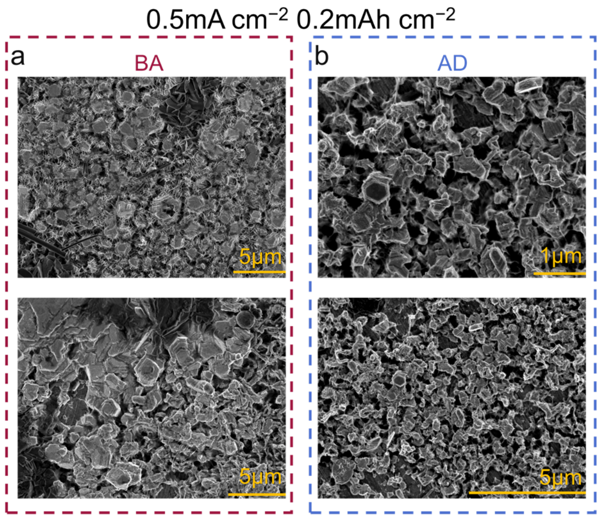

Supplementary Fig. 12 SEM images of Zn crystal nuclei during electro-deposition (0.5 mA cm^{-2} 0.2 mAh cm^{-2}) of BA and AD electrolyte on Zn foil.

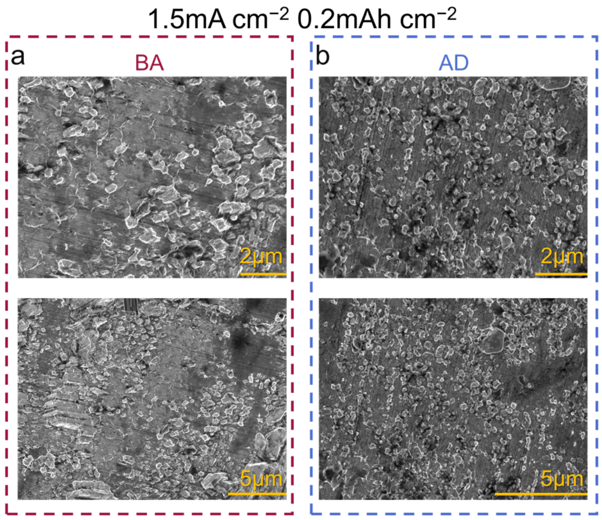

Supplementary Fig. 14 SEM images of Zn crystal nuclei during electro-deposition (1.5 mA cm^{-2} 0.2 mAh cm^{-2}) of BA and AD electrolyte on Zn foil.

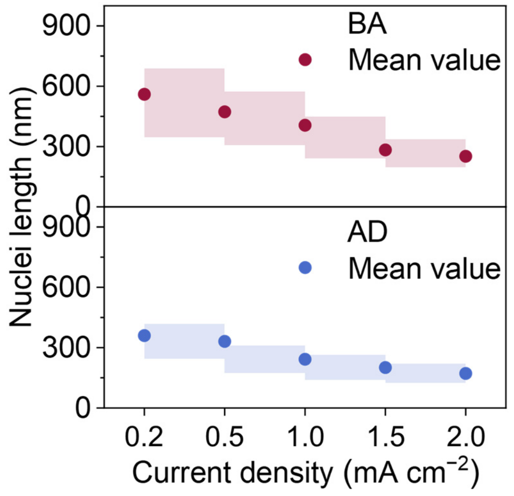

Supplementary Fig. 16 The average size and variance of Zn crystal nuclei during electro-deposition at different current densities (0.2, 0.5, 1.0, 1.5, and 2.0 mA cm^{-2}) to achieve a surface capacity of 0.2 mAh cm^{-2}.

Original comment 1-3

The manuscript derives a theoretical relationship between overpotential and current density under symmetric-cell conditions (Eq. 8). It is recommended that this relationship be quantitatively compared with experimental data, which would provide strong additional validation of the proposed model. Such results could be included in the main text or as supplementary information.

Response

We appreciate the Reviewer's insightful comment. As illustrated in **Supplementary Fig. 9**, the measured nucleation overpotentials in symmetric cells at various current densities closely align with the theoretical predictions from **Equation (8)**. Furthermore, quantitative analysis via curve fitting yielded charge-transfer coefficients at the electrode-electrolyte interface: $a_{HW} = 0.243$ and $a_{LW} = 0.196$. These results underscore the validity of employing Zn||Zn symmetric cell measurements as a robust framework for evaluating interfacial charge-transfer kinetics.

In response, to address this comment, the relevant figure has been added as **Supplementary Fig. 9** in **R-SI** (**page 20**). Moreover, the additional descriptions have also been included in **R-MS** (**page 6**) and **R-SI (page 20)**:

'As derived from the Butler-Volmer relation (Supplementary Equation (27)), the current density and overpotential during cross-flow electrodeposition in symmetric cells satisfy Equation (8) (derivation in Supplementary Note 3). Accordingly, under galvanostatic conditions, the overpotential η increases with increasing current density, a trend confirmed experimentally (Supplementary Fig. 9).' in ***R-MS (page 6)***

'By analyzing the dependence of the nucleation overpotential on current density, we extracted the interfacial charge-transfer coefficient for the HW electrolyte as a_{HW} = 0.243, which is larger than that for the LW electrolyte (a_{LW} = 0.196). This difference indicates faster interfacial charge-transfer kinetics in the HW electrolyte.' in ***R-SI (page 20)***

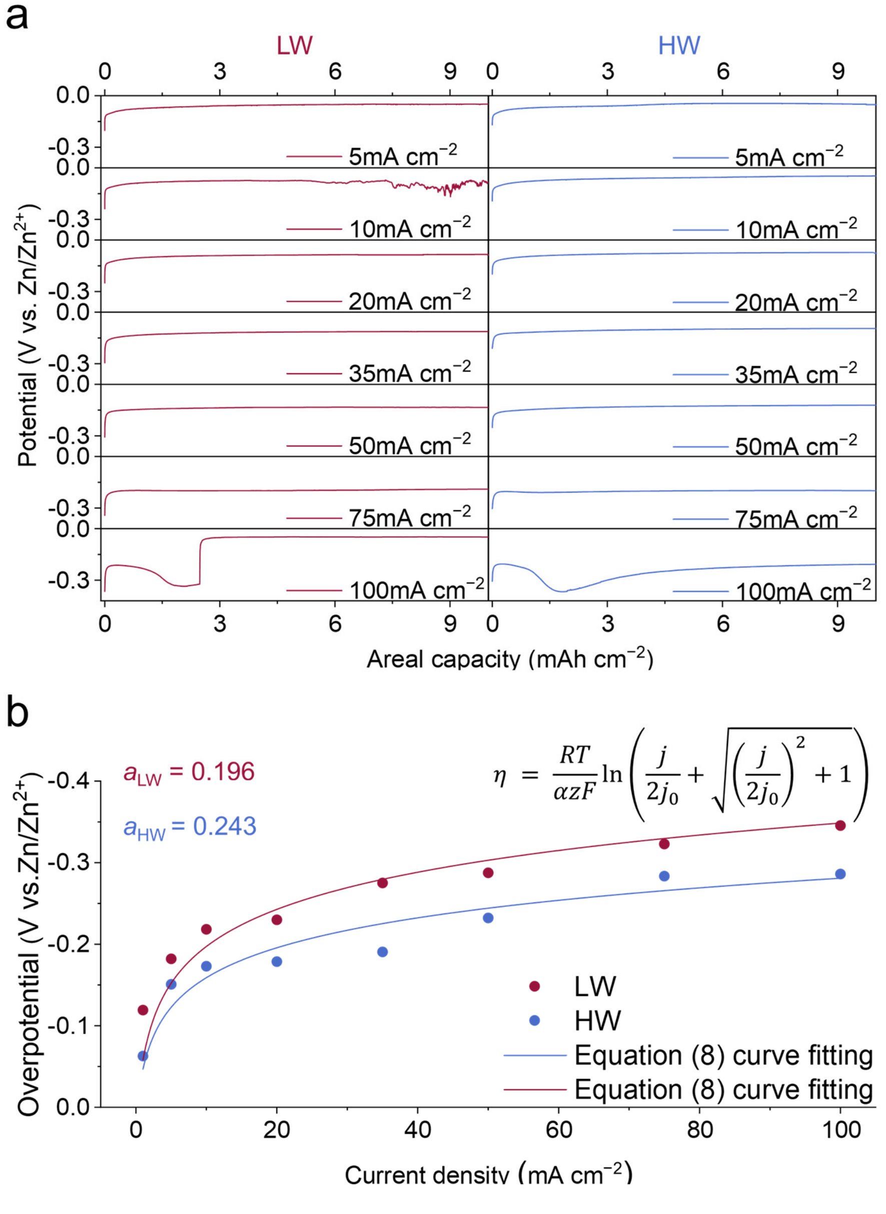

Supplementary Fig. 9 (a) Voltage curves of Zn||Zn (100 μm) symmetric cells using LW and HW electrolytes at current densities of 5, 10, 20, 35, 50, 75, and 100 mA cm^{-2}. (b) Curve fitting of the nucleation overpotential as a function of current density based on Equation (8) (Supplementary Equation (29)).

Original comment 1-4

For clarity, the authors should explicitly state that all physical quantities involved in the dimensionless descriptor are defined on a per-unit-area basis. This clarification would help avoid potential ambiguity in interpretation and application.

Response

We appreciate the Reviewer's constructive suggestion. Accordingly, we have incorporated a detailed derivation of the dimensional normalization process in the **R-SI (page 8)**. Furthermore, we now explicitly specify that all extensive physical quantities are normalized per unit area. This addition elucidates the physical significance of the dimensionless factor, ensuring its consistent interpretation and application by the reader.

In response to this comment, the relevant equation has been added as **Supplementary Equation (46)**. Moreover, an additional description has also been included in **R-SI (page 8)**:

*'To examine its dimensionless nature, η has units of V, i_{corr} has units of A, and R_{ct} has units of Ω. Each term is measured per unit area. Substituting these into **Supplementary Equation (46)** confirms that the quantity is properly normalized and thus satisfies the definition of a dimensionless factor.*

$$[W_f] = \frac{V}{(A)(\Omega)} = 1 \tag{46}$$

*' in **R-SI (page 8)***

Original comment 1-5

In Figure 3f, the authors simulate the post-deposition surface potential distribution using an electrostatic model. While the qualitative trends appear reasonable, a more physically consistent approach would be to simulate the surface potential under an imposed current-density boundary condition at the electrode surface, which would more directly reflect realistic electrodeposition conditions.

Response

We appreciate the Reviewer's insightful suggestion. To address this concern, we have performed additional galvanostatic simulations to evaluate the surface electric potential distribution and the corresponding interfacial field response at a current density of 5 mA cm^{-2}. As illustrated in **Fig. 3f**, the HW electrolyte exhibits a superior uniformity in surface potential compared to the LW electrolyte (variation < 0.16 V). These results more closely replicate the electrode behavior under practical battery operating conditions, thereby reinforcing the conclusions drawn from our previous potentiostatic analysis.

In response to this comment, the relevant figure has been added as **Fig. 3f** in the **R-MS** (**page 9**). An additional description has also been included in **R-MS** (**page 8**):

*'Finite-element simulations reveal that, at a current density of 5 mA cm^{-2}, the HW maintains a more uniform surface electric-potential distribution (variation < 0.16 V) than LW (Fig. 3f), thereby mitigating tip-induced parasitic reactions and inhibiting dendrite propagation[50].' in **R-MS (page 8)***

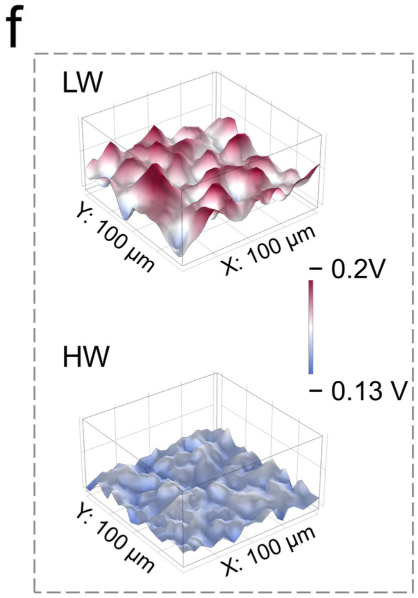

Figure 3. Dimensionless quantification of electrodeposition kinetics. f Finite-element simulations of the surface electric-potential distribution.

Original comment 1-6

In Figure 4, the designed electrolyte exhibits excellent corrosion resistance and suppression of side reactions, as claimed in the manuscript. A more detailed discussion of the underlying mechanisms at the electrolyte-Zn anode interface, such as solvation structure regulation, interfacial reaction pathways, or electric-field modulation, would further strengthen the interpretation of these results.

Response

We appreciate the Reviewer's insightful comments regarding the interfacial chemistry. Accordingly, we have incorporated a new schematic (**Supplementary Fig. 29**) to elucidate the multifaceted role of additive molecules at the electrode/electrolyte interface. The illustration demonstrates how the preferential adsorption of additives shields the electrode surface during Zn^{2+} electrodeposition, thereby effectively suppressing the hydrogen evolution reaction (HER) and attenuating local pH fluctuations. Consequently, the formation of insulating basic zinc sulfate (ZHS) by-products is significantly inhibited. This addition provides a more robust mechanistic framework for understanding the stabilized Zn interface.

In response to this comment, the relevant figure has been added as **Supplementary Fig. 29** in **R-SI** (**page 40**). Moreover, an additional description has also been included in **R-MS** (**page 10**):

'These stable chemical interfaces are attributed to the adsorption of additive molecules at the electrode surface (Supplementary Fig. 29).' in ***R-MS (page 10)***

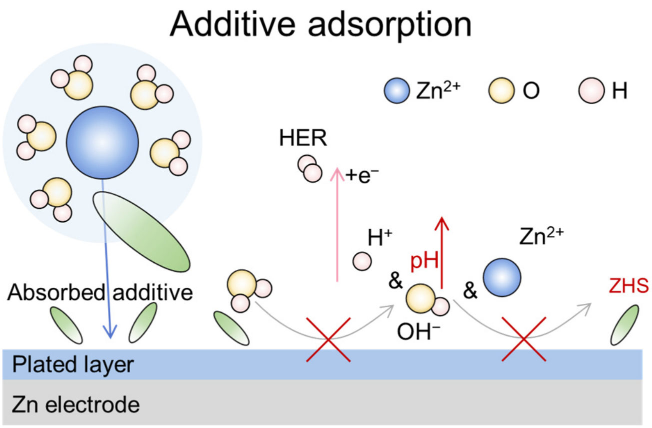

Supplementary Fig. 29 Schematic illustration of an additive-induced protective interphase on the Zn electrode.

Response to Reviewer #2

Remarks to the Author

The manuscript titled "Quantifying Interfacial Electrochemistry in Aqueous Batteries: A Dimensionless Factor via Revisiting Zn-Specific Nucleation Modeling" introduces promising concept by linking a new electrochemical descriptor to Zn electrodeposition stability. However, it currently over-relies on simplified models and limited experimental validation. The authors must deepen the theoretical framework, rigorously validate the dimensionless factor W_f both experimentally and via simulations, incorporate advanced operando and microstructural analyses, and address practical considerations before drawing broad, mechanistically grounded conclusions. After these minor revisions, the work will meet the high standards for publication.

Response

We sincerely thank Reviewer #2 for the insightful assessment and constructive feedback. The suggestions, regarding the theoretical framework, the evaluation of the W_f descriptor, the expansion of operando and microstructural characterizations, and the discussion of practical implications, have been instrumental in improving the manuscript. We have addressed these points through systematic revisions in the **R-MS** and **R-SI**.

We appreciate the Reviewer's acknowledgment of W_f as a promising concept. The W_f is designed as a compact, dimensionless descriptor that normalizes diverse physical quantities into a unified scale. This approach facilitates direct comparison across disparate electrochemical testing conditions while maintaining sensitivity to the complex interplay between mass transport, interfacial chemistry, and reaction kinetics. In principle, W_f aligns with established dimensionless numbers used to quantify competing processes in electrochemical systems, such as the Damkohler number (*Combust. Flame. 1992, 88, 37-49*), the Peclet number *(Transport Phenomena, 2nd ed; Wiley, New York, 2002*), and the Thiele moduli (*Ind. Eng. Chem. Res. 2020, 59, 6874-6885*).

Physically, W_f is defined as the ratio of driving force (overpotential) to the interfacial resistance metric (the product of corrosion current and polarization resistance). Consequently, a higher W_f value reflects either a stronger driving force or a reduced interfacial resistance, both of which correlate with enhanced deposition efficiency. For Zn electrodes in aqueous electrolytes, where parasitic side reactions are thermodynamically favored by the proximity of the Zn standard potential to the hydrogen evolution potential, this driving-force/resistance-based descriptor provides a robust, engineering-oriented metric for the rational design of electrolytes and additives.

Furthermore, we have taken the Reviewer's concerns regarding the depth of characterization seriously. Relevant experimental data and analyses have now been adequately added to the **R-MS** and **R-SI** and are discussed in detail in our point-by-point response. We thank the Reviewer again for the helpful and encouraging comments.

Original comment 2-1

This work proposes dimensionless W_f parameter as a "universal" descriptor quantitatively coupling electrodeposition driving force and interfacial resistance. However, the validation of W_f relies

primarily on some literature-derived data points and limited in-situ measurements, which are insufficient for establishing "universal" applicability. There is a notable absence of systematic, controlled experimental datasets spanning a broad parameter space (e.g., "different" electrolyte chemistries, concentrations, additives, and operational conditions) that explicitly test the correlation between computed W_f values and actual nucleation, morphology, and cycle life.

The authors should incorporate rigorous, quantitative validation through systematically controlled electrochemical measurements, e.g., electrochemical impedance spectroscopy over a wide frequency range, in conjunction with microscopy-characterized nucleation behavior, to directly correlate experimental W_f calculations with "microstructural evolution".

Response

We appreciate the Reviewer's insightful and constructive feedback, which has significantly improved the quality of this manuscript. To evaluate the broader applicability of the W_f descriptor, we have expanded the literature survey (**Supplementary Table 6**) to encompass a wider range of zinc electrolyte chemistries: zinc sulfate ($ZnSO_4$), zinc chloride ($ZnCl_2$), zinc trifluoromethanesulfonate ($Zn(OTf)_2$), zinc bis(trifluoromethanesulfonyl)imide ($Zn(TFSI)_2$), and zinc perchlorate ($ZnClO_4$). Analysis of these datasets reveals a consistent increase in W_f across the cited systems (**Fig. 5c**), providing further evidence for the descriptor's generality.

Furthermore, we have refined the manuscript's terminology to ensure scientific rigor. Following the Reviewer's suggestion, we have moved away from claims of "universality" and instead defined W_f as a generally applicable descriptor that correlates the electrodeposition driving force and interfacial resistance. This adjustment, coupled with the expanded literature context, offers a more nuanced and explicit account of the study's scope and conclusions.

To establish a more direct and transparent link between the W_f and microstructural evolution, we have consolidated our analysis by pairing controlled electrochemical measurements with microscopy. Each computed W_f value is now directly compared with complementary kinetic and morphological indicators, including nucleation overpotential curves, electrochemical impedance spectroscopy (EIS), chronoamperometry transients (**Fig. 2c** and **Supplementary Fig. 18)**, SEM imaging, and the Zn charge-transfer coefficient**,** and the Zn charge-transfer coefficient in **Supplementary Fig. 9**. This multimodal approach probes both interfacial kinetics and early-stage morphology, facilitating a more coherent, mechanistic comparison between the calculated descriptor and experimentally observed nucleation behavior. Regarding the Reviewer's concern over concentration and operational variations, we emphasize that the ratio $W_{f\,Additive}$ / $W_{f\,Bare}$ effectively removes many extrinsic, system-level scaling factors (e.g., cell geometry, effective electrochemically active surface area, imposed current density, and bulk ionic conductivity) and therefore normalizes the various factors that differ across electrolyte systems.

Finally, we acknowledge that exhaustive factorial validation across all possible chemistry, concentration, additive family, and operating regimes lies beyond the scope of this work. Accordingly, the manuscript frames the current contribution as providing strong, cross-chemistry evidence that W_f

is a robust and generally applicable descriptor. Collectively, these results demonstrate that a higher W_f corresponds to accelerated reaction kinetics, aligning with the proposed mechanistic framework.

In response to this comment, the corresponding materials have been added: **Figs. 5b** and **c** to the **R-MS** (**page 13**), **Supplementary Fig. 18** to the **R-SI** (**page 29**), and **Supplementary Table 6** to the **R-SI** (**page 55**). In addition, an expanded description has also been incorporated into the **R-MS** (**page 6**): *'Supplementary Fig. 18 presents further characterization of the interfacial kinetics of the electrode after nucleation under the specified current density and areal capacity conditions. The AD electrolyte shows a higher current response and faster interfacial kinetics in the CA test, which are associated with a higher nucleation site density.'* in ***R-MS (page 6)***

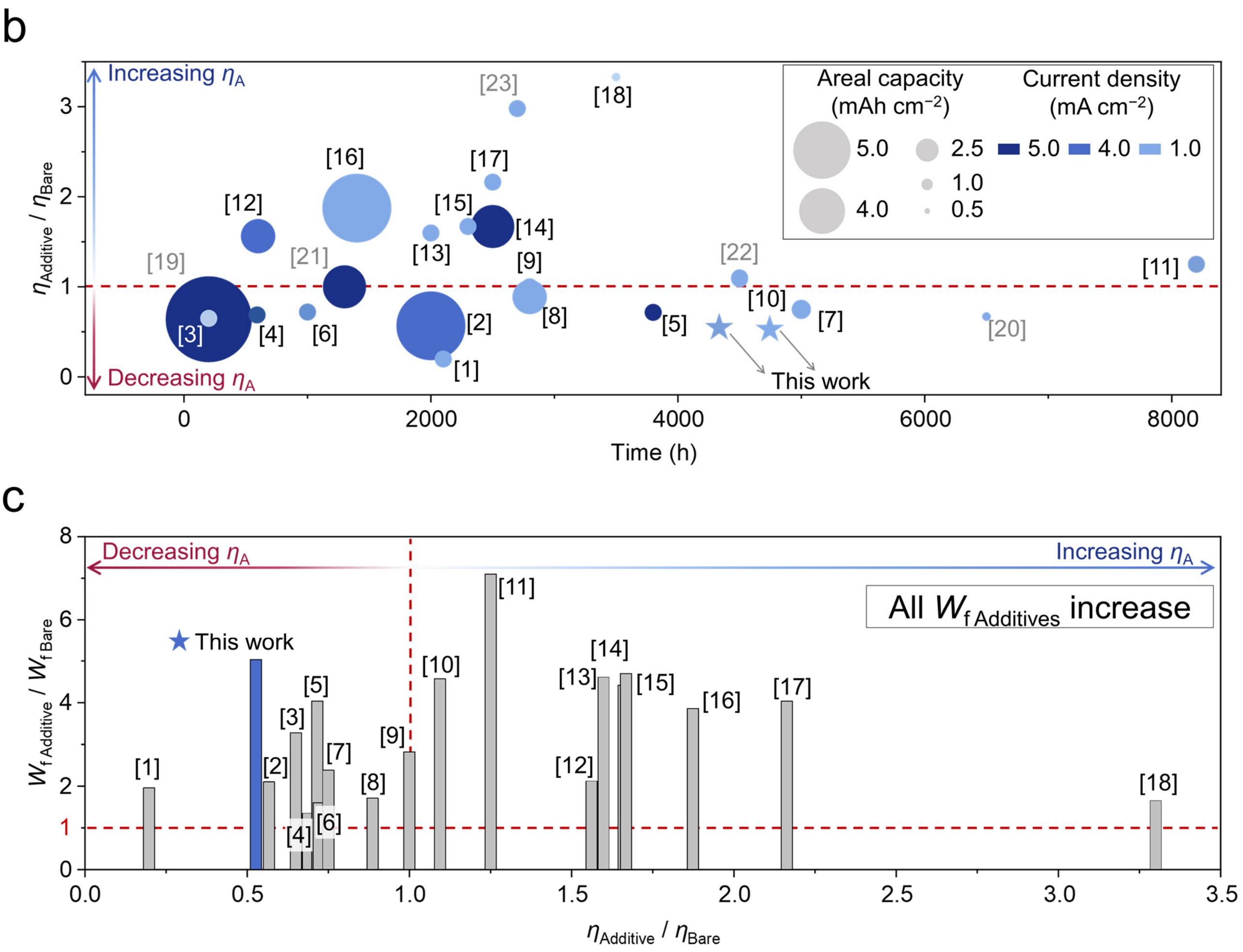

Figure 5. Analysis and verification of the dimensionless descriptor. b Statistical correlations among cycling time, current density, areal capacity, and polarization magnitude for various additives. **c** Statistics of the dimensionless descriptor W_f reported in related works (serial numbers correspond to Supplementary Table 6).

Supplementary Table 6 Statistics related to electrolyte additives and calculation of W_f.

Sequence number	Additives	$\eta_{Additive} / \eta_{Bare}$ (↓ or ↑)	$W_{f\,Additive} / W_{f\,Bare}$ (↓ or ↑)	Citation
[1]	2 M $ZnSO_4$ + 0.2 M 3,3′-dithiobis-1-propanesulfonic acid disodium salt	0.198 (↓)	1.96 (↑)	39
[2]	1 M $ZnSO_4$ + 0.1 M HTFSI	0.567 (↓)	2.1 (↑)	40
[3]	30 m $ZnCl_2$ + 5 mg vanillin	0.65 (↓)	3.28 (↑)	41
[4]	0.5 M $ZnCl_2$ + 1 M 1-ethyl-3-methyl imidazolium iodide	0.688 (↓)	1.35 (↑)	42
[5]	2 M $ZnSO_4$ + 0.1 M Lithium bis(oxalate)borate	0.716 (↓)	4.04 (↑)	43
[6]	$ZnCl_2 \cdot 3H_2O$ + 0.1g cellulose + 0.1 g PEG	0.72 (↓)	1.60 (↑)	44
[7]	2M $Zn(OTf)_2$ + 50% H_2O + 50% DME	0.750 (↓)	2.38 (↑)	45
[8]	2 M $ZnSO_4$ + 0.5 mg mL^{-1} Fu	0.886 (↓)	1.71 (↑)	46
[9]	2 M $Zn(OTf)_2$ + PVEMA	1	2.82 (↑)	27
[10]	2 M $Zn(ClO_4)_2$ + PMCNA	1.094 (↑)	4.57 (↑)	47
[11]	0.5 M $Zn(TFSI)_2$ 1,3-dioxolane (DOL) / H_2O = 2 / 1	1.25 (↑)	7.10 (↑)	48
[12]	2 M $ZnSO_4$ + 0.5 g L^{-1} saccharin	1.562 (↑)	2.12 (↑)	49
[13]	1 M $ZnSO_4$ + 10 mM Glucose	1.598 (↑)	4.61 (↑)	50
[14]	1 M $Zn(CF_3SO_3)_2$ + n-propyl alcohol	1.660 (↑)	4.42 (↑)	28
[15]	2 M $ZnSO_4$ + 0.2 M PNO	1.667 (↑)	4.70 (↑)	26
[16]	1 m $ZnSO_4$ + 4 m Glycerophosphorylcholine	1.874 (↑)	3.86 (↑)	51
[17]	2 M $ZnSO_4$ + 2.0 wt% Tween-20	2.163 (↑)	4.04 (↑)	52
[18]	2 M $ZnSO_4$ + 0.12 M pyridine	3.3 (↑)	1.65 (↑)	53
[19]	3 M $ZnSO_4$ + 10 mM Cyclodextrins	0.64 (↓)	Data scarcity	54
[20]	1 M $ZnSO_4$ + 5 mM D-mannose and sodium lignosulfonate	0.67 (↓)	Data scarcity	55
[21]	2 M $ZnSO_4$ + Trehalose	1	Data scarcity	56
[22]	2 M $ZnSO_4$ + DMS + LS	1.1 (↑)	Data scarcity	57
[23]	$ZnSO_4$ + 50 mM methylurea	2.98 (↑)	Data scarcity	58

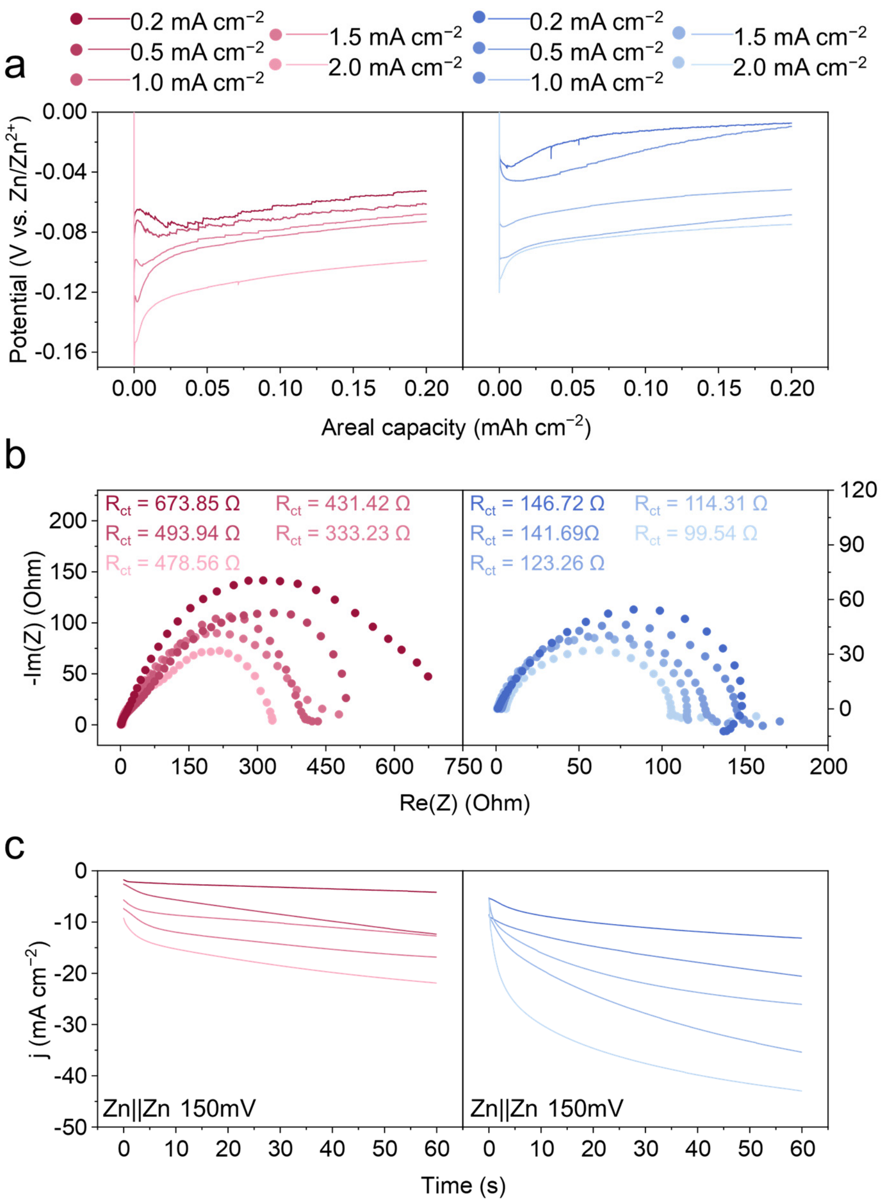

Supplementary Fig. 18 Zn||Zn (100 μm) symmetric cells with a deposition capacity of 0.2 mAh cm^{-2} (0.2, 0.5, 1.0, 1.5, and 2.0 mA cm^{-2}) (**a**) deposition voltage curves (**b**) electrochemical impedance spectroscopy (EIS) measured after deposition (**c**) chronoamperometric (CA) current-time curves recorded after deposition.

Original comment 2-2

The derivation of W_f appears to rest on a simplified model approximation, e.g., the ratio of polarization potential to the product of corrosion current and polarization resistance (Eq. 9). This oversimplification neglects possible nonlinearities inherent in electrochemical processes, such as the influence of double-layer capacitance, non-ideal solution behavior, surface heterogeneities, and complex parasitic reactions. There is scant theoretical underpinning or derivation from first principles justifying the form of W_f, particularly how the interplay of these parameters universally governs the nucleation mode and

stability across different systems. So, it would be better to consider the fundamental thermodynamic and kinetic principles (including non-ideal solution effects) while deriving the W_f, to establish its theoretical generality.

Response

Many thanks for the Reviewer's professional comments. We acknowledge that the expression $W_f = \eta / (i_{corr} \cdot Z)$ is a simplified form. It is noticed that the expression has precedent in classical electrochemical theory and is reasonable under well-defined conditions. By applying the Stern-Geary approach, the Butler-Volmer equation can be linearized under sufficiently small potential perturbations (typically within about ±5~10 mV around the corrosion potential E_{corr}). This linearization establishes a reciprocal relationship between the polarization resistance Z and the corrosion current i_{corr} (*J. Electrochem. Soc. 1957, 104, 56; PCCP. 2021, 23, 21097-21105*). To justify this link, a detailed step-by-step derivation in the linear regime has been included in the **R-SI (pages 6-8)**.

In practical application, particularly for the aqueous battery systems investigated here, the low-frequency impedance Z serves as a reliable proxy for the polarization resistance. As detailed in the **R-SI**, under steady-state conditions, absent significant diffusion or double-layer interference, Z primarily comprises the solution resistance R_s and the charge transfer resistance R_{ct}. Specifically, Z converges to R_s in the high-frequency limit and to $R_s + R_{ct}$ in the low-frequency limit (*ACS Meas. Sci. Au. 2023, 3, 162-193*). Given that R_{ct} is typically orders of magnitude larger than R_s in the aqueous systems, the contribution of R_s can be neglected ($Z \approx R_{ct}$). Consequently, W_f can be practically approximated as $\eta / (i_{corr} \cdot R_{ct})$, a term that underscores the balance between the electrochemical driving force and the resulting corrosive flux.

W_f thus functions as a parsimonious, dimensionless operator. It projects complex, atomistic interactions (e.g., surface energy, desolvation energy, charge transfer) onto a set of macroscopic, measurable parameters, as a physical descriptor that captures the dominant coupling between transport and interfacial kinetics. By design, this metric subsumes higher-order complexities, including double-layer capacitance and surface heterogeneities, into its constituent electrochemical parameters. This approach enables a direct, macroscopic comparison of interfacial stability across diverse electrolyte systems without requiring explicit modeling of every secondary interaction.

During the derivation of W_f, thanks to the Reviewer's kind reminder, we have carefully examined the fundamental thermodynamic and kinetic principles to establish its theoretical generality. Taking these limitations into account, the simplified form of W_f has a clear physical interpretation and practical utility: it serves as a reliable proxy for assessing the balance between interfacial driving potential and resistance, offering a convenient metric for comparative studies of nucleation behavior.

We thank the Reviewer again for the valuable suggestion and note that we have strengthened the derivation and the accompanying discussion accordingly. In response to this comment, the relevant content has been added under the **R-SI** (**pages 6-8**):

'In addition, the polarization resistance is defined as the reciprocal of the small-signal slope near the polarization potential (η, overpotential).

$$Z = \left(\frac{\mathrm{d}j}{\mathrm{d}E}\right)_{\eta\to 0}^{-1} \tag{32}$$

$$\left(\frac{\mathrm{d}j}{\mathrm{d}E}\right)_{\eta\to 0} = j_0 \frac{zF}{RT}(\alpha_{\mathrm{anode}} + \alpha_{\mathrm{cathode}}) \tag{33}$$

$$Z = \frac{RT}{j_0 zF} \frac{1}{(\alpha_{\mathrm{anode}}+\alpha_{\mathrm{cathode}})} \tag{34}$$

*The anodic and cathodic Tafel slopes are defined by **Supplementary Equations (35) and (36)**, respectively.*

$$\beta_{\mathrm{anode}} = \frac{2.303\,RT}{\alpha_{\mathrm{anode}} zF}\ ,\ \beta_{\mathrm{cathode}} = \frac{2.303\,RT}{\alpha_{\mathrm{cathode}} zF} \tag{35)\&(36}$$

The units are typically expressed as millivolts per decade of current. In corrosion kinetics analysis, the anodic and cathodic polarizations are often treated as independently manifested and can be approximated as follows:

$$j_{\mathrm{anode}} \approx j_{\mathrm{corr}} 10^{\eta/\beta_{\mathrm{anode}}}\ ,\ j_{\mathrm{cathode}} \approx j_{\mathrm{corr}} 10^{\eta/\beta_{\mathrm{cathode}}} \tag{37)\&(38}$$

In the small-perturbation regime, the exponential terms can be expanded using a first-order approximation. Substituting into the net current expression $j = j_{\mathrm{anode}} - j_{\mathrm{cathode}}$ *, and expanding with respect to small overpotential η, **Supplementary Equation (39)** is obtained.*

$$j \approx \eta j_{\mathrm{corr}} \left[\ln(10)\left(\frac{1}{\beta_{\mathrm{anode}}} + \frac{1}{\beta_{\mathrm{cathode}}}\right)\right] \tag{39}$$

*Therefore, the above **Supplementary Equation (33)** can be rewritten as:*

$$\left(\frac{\mathrm{d}j}{\mathrm{d}E}\right)_{\eta\to 0} = j_{\mathrm{corr}} \left[\ln(10)\left(\frac{1}{\beta_{\mathrm{anode}}} + \frac{1}{\beta_{\mathrm{cathode}}}\right)\right] \tag{40}$$

Therefore, Z can be obtained as:

$$Z = \left(\frac{\mathrm{d}j}{\mathrm{d}E}\right)_{\eta\to 0}^{-1} = \frac{1}{j_{\mathrm{corr}} \ln(10)} \frac{1}{\left(\frac{1}{\beta_{\mathrm{anode}}}+\frac{1}{\beta_{\mathrm{cathode}}}\right)} = \frac{\beta_{\mathrm{anode}} * \beta_{\mathrm{cathode}}}{2.303 j_{\mathrm{corr}}(\beta_{\mathrm{anode}}+\beta_{\mathrm{cathode}})} \tag{41}$$

Thus, on a unit area basis, the Stern-Geary equation is obtained:[1-3]

$$i_{\mathrm{corr}} * Z = \frac{\beta_{\mathrm{anode}} * \beta_{\mathrm{cathode}}}{2.303(\beta_{\mathrm{anode}}+\beta_{\mathrm{cathode}})} = B \tag{42}$$

Therefore, the dimensionless factor W_f *is defined as the ratio of the overpotential in the nucleation process to the product of the corrosion current and the polarization resistance (**Supplementary Equation (34)**).*

$$W_{\mathrm{f}} = \frac{\Delta E}{I \times Z} = \frac{\eta}{B} \tag{43}$$

Here, ΔE denotes the nucleation overpotential (η), I the corrosion current, and Z the polarization resistance.

Electrochemical impedance spectra can often be approximated using a simple equivalent circuit. A commonly adopted model consists of the solution resistance R_{s} *in series with a parallel combination of the charge transfer resistance* R_{ct} *and the double-layer capacitance* C_{dl} *, as shown in **Supplementary Equation (44)**.*

$$Z(\omega) = R_\mathrm{s} + \frac{1}{\frac{1}{R_\mathrm{ct}} + j\omega C_\mathrm{dl}} = R_\mathrm{s} + \frac{R_\mathrm{ct}}{1 + j\omega R_\mathrm{ct} C_\mathrm{dl}} \quad (44)$$

Here ω denotes the angular frequency. In the high-frequency limit, the magnitude of the second term tends to zero, so $Z(\omega \to \infty) \approx R_\mathrm{s}$. In the low-frequency limit, $Z(\omega \to 0) \approx R_\mathrm{s} + R_\mathrm{ct}$. Because R_ct is much larger than R_s in the present system (Zn||Zn asymmetric cells), the contribution of R_s can be neglected, the polarization resistance therefore dominates the low-frequency real part of the impedance. Under typical conditions, in which no significant diffusion-controlled processes or pseudocapacitive behavior are present, and an ideal capacitor can approximate the interfacial capacitance, this polarization resistance is equivalent to the charge-transfer resistance obtained from fitting, namely $Z \approx R_\mathrm{ct}$, as described in Supplementary Equation (45).[3-5]

$$W_\mathrm{f} = \frac{\eta}{i_\mathrm{corr} \times R_\mathrm{ct}} \quad (45)$$

' in ***R-SI (pages 6-8)***

Original comment 2-3

While the authors extend CNT to account for Zn's HCP crystal structure by modeling anisotropic nucleation, they do not sufficiently demonstrate that their modified nucleation energy barrier and critical nucleus size calculations incorporate all relevant atomic-scale energetic contributions. The reliance on classical expressions neglects the non-classical, non-spherical, or mesoscopic nucleation phenomena such as 2D nucleation, defect-mediated nucleation, or kinetic pathways involving pre-activation clusters. The implications of such pathways on deposition morphology, dendrite formation, and cycle life are underrepresented. It would be great to see if the authors could extend the theoretical modelling with molecular dynamics simulations to explore these circumstances.

Response

We appreciate the Reviewer's thoughtful engagement with the theoretical underpinnings of our work, particularly regarding the role of non-classical and atomistic nucleation pathways. We recognize that different theoretical frameworks are optimized for distinct spatiotemporal scales: ab initio molecular dynamics (AIMD) and DFT-based calculations excel at resolving short-range bonding and pre-nucleation clusters (*Proc. Natl. Acad. Sci. U. S. A. 2016, 113, E4937-E4945*); Reactive force field (ReaxFF) molecular dynamics bridge the gap to larger-scale chemical reactivity (*npj Comput. Mater. 2016,2, 15011*); Kinetic Monte Carlo (KMC) and Phase-field models are better suited for defect-mediated migration and mesoscale morphology evolution, respectively (*Front. Phys. 2021, 9, 631918; Phys. Rev. E. 2015, 92, 011301*). Within this hierarchy, our application of Classical Nucleation Theory (CNT), augmented by anisotropic corrections, represents a deliberate choice to provide a descriptor that is both computationally tractable and experimentally parameterizable.

In this work, we specifically tailored the CNT framework to the HCP crystal structure of Zn. This approach was motivated by two primary requirements. First, by integrating lattice and interfacial anisotropy into the CNT framework, the crystallographic symmetry and the orientation-dependent surface-energy landscape can be formally captured through analytical expressions (*Phys. Rev. 1951,*

82, 87). This facilitates a direct link between theoretical energy barriers and experimentally accessible quantities such as nucleation overpotential, polarization resistance, and corrosion current. Second, our central objective is to develop W_f as an engineering-oriented descriptor for the rapid screening of electrolytes and additives. Accordingly, the major energetic contributions considered in our model, including bulk free-energy driving force, anisotropic surface energy, and interfacial kinetic dissipation (resistance), together with the approximations made and their domains of applicability, are explicitly stated in the **R-SI**.

Notably, while atomic-scale energetic perturbations might shift the absolute values of the nucleation barrier or critical nucleus size, our sensitivity analysis confirms that the stability trends derived from W_f remain robust within the investigated experimental window. This suggests that these higher-order corrections do not fundamentally alter the comparative ranking of deposition stability across disparate electrolyte systems. Consequently, we propose W_f as a trend-based criterion rather than an atomistically exhaustive metric.

We fully concur with the Reviewer regarding the scientific importance of molecular dynamics (MD) or reactive MD in elucidating early-stage interfacial structures and defect-assisted pathways (*Science, 2026, 391,622-627*). However, faithfully capturing aqueous electrodeposition at the atomic scale remains challenging. Accounting simultaneously for electrode potential, explicit solvent structure, ion coordination, and chemical reactivity often requires carefully parameterized reactive force fields or ab initio molecular dynamics, both of which are computationally demanding. To bridge the gap to experimentally relevant scales, researchers frequently complement these simulations with enhanced-sampling methods or multiscale coupling strategies. In addition, establishing a direct, quantitative connection between atomistic energy landscapes and experimentally measurable macroscopic descriptors, such as overpotential, charge-transfer resistance, or W_f, constitutes a substantial methodological challenge (*Materials (Basel), 2021, 14(6), 1408)*.

Therefore, while fully recognizing the importance of non-classical nucleation pathways and atomistic simulations, we consider these investigations to be beyond the central scope of the present study. The focus of this work is to propose a practically measurable descriptor and to validate its predictive power through electrochemical experiments and mesoscale analyses. The approximations involved and the limits of applicability of our theoretical framework are stated transparently in the **R-MS** and the **R-SI**. Systematic atomistic simulations that explicitly address non-classical nucleation pathways are identified as an important and meaningful direction for future work.

Original comment 2-4

Although SEM images and in-situ optical microscopy suggest improved morphological stability in high-W_f electrolytes, these characterizations are inadequate for conclusively linking the W_f value with nucleation and growth phenomena at the atomic and mesoscale. The deposition process is inherently stochastic, and factors such as grain orientation, defect density, and local current distribution critically influence dendrite suppression. The study overlooks advanced in-situ or operando techniques capable of capturing real-time, three-dimensional evolution of nucleation sites and dendritic growth modes.

Without such data, the assertion that W_f predicts, and guides suppression of dendrite formation remains phenomenological. The authors may consider the operando electron microscopy (e.g., in-situ TEM or synchrotron-based X-ray tomography) during cycling, coupled with microstructural analysis, to directly observe nucleation sites, growth fronts, and dendritic features. It would be more impressive if the authors can quantify the correlation between computed W_f and microstructural parameters such as nucleation density, tip growth rates, and defect distribution.

Response

We appreciate the Reviewer's constructive suggestion regarding the multiscale characterization of morphological evolution. We agree that operando techniques, such as in-situ TEM and synchrotron X-ray imaging, are highly valuable for directly resolving the stochastic nature of nucleation and dendritic growth. However, performing in-situ TEM in an aqueous system is technically challenging due to potential water leakage and electron beam-induced radiolysis. These effects can generate byproducts, such as hydrogen and oxygen, which render the observed kinetics inconsistent with the intrinsic state of the system (*R. Soc. Open Sci. 2020, 7, 191204*). Furthermore, the constrained cell geometry and specific operating conditions required for these techniques may not fully represent practical devices (*Nat. Commun. 2017, 8, 15806*). Similarly, synchrotron X-ray imaging at high photon flux can cause sample heating and enhanced side reactions, posing significant challenges for aqueous electrolytes (*Chem. Mater. 2024, 36, 5596-5610*).

Although transforming the electrolyte into a quasi-solid gel form might reduce experimental complications, it fundamentally alters the transport regime and interfacial thermodynamics of the aqueous system. We note that the primary objective of this study is to propose and validate a descriptor based on measurable parameters with practical engineering applicability. We intend to provide a metric that can predict and compare deposition stability under various electrolyte conditions rather than to image atomic-scale dynamics at a single nucleation site. High spatiotemporal-resolution operando electron microscopy and synchrotron tomography typically require highly customized experimental setups and complex data-reconstruction workflows. While we hope to implement these advanced techniques in our future work, we have instead employed a home-made specialized technology known as in-situ Electrochemical Laser Scanning Confocal Microscopy (ELSCM) to analyze microstructural changes quantitatively.

To address the concern that the correlation between W_f and nucleation or growth behavior remains largely phenomenological, we have incorporated new quantitative data and statistical analyses in the **R-MS**. These additions include ELSCM surface reconstructions of dendritic growth under different W_f conditions, shown in **Supplementary Figs. 21 a** and **b**, and compositional analyses of the corresponding interfaces, presented in **Supplementary Figs. 21 c** and **d**. The new data indicate that W_f provides meaningful discrimination of deposition stability across the examined systems.

Overall, this quantitative evidence strengthens the link between W_f and morphology stability while preserving the simplicity and generality of the descriptor for experimental screening and engineering use. We thank the Reviewer for this important suggestion, which has helped clarify the positioning of our study and identify useful directions for our future research.

In response to this comment, the relevant figure has been added as **Supplementary Fig. 21** in the **R-SI (page 32)**. An additional description has also been included in the **R-MS (page 8)**: *'Furthermore, in-situ monitoring of dendrite growth (Supplementary Fig. 21) during Zn deposition at a current density of 5 mA cm^{-2}, with interface reconstruction every 10 s, reveals the detailed growth process. In the LW, dendrite height increases substantially from 18.89 μm at 0 s to 75.44 μm at 60 s. This sharp increase indicates a high risk of separator piercing. In contrast, the dendrite height in HW rises only modestly from 18.26 μm at 0 s to 24.93 μm at 60 s, resulting in a more uniform and stable deposition interface.'* in **R-MS (page 8)**

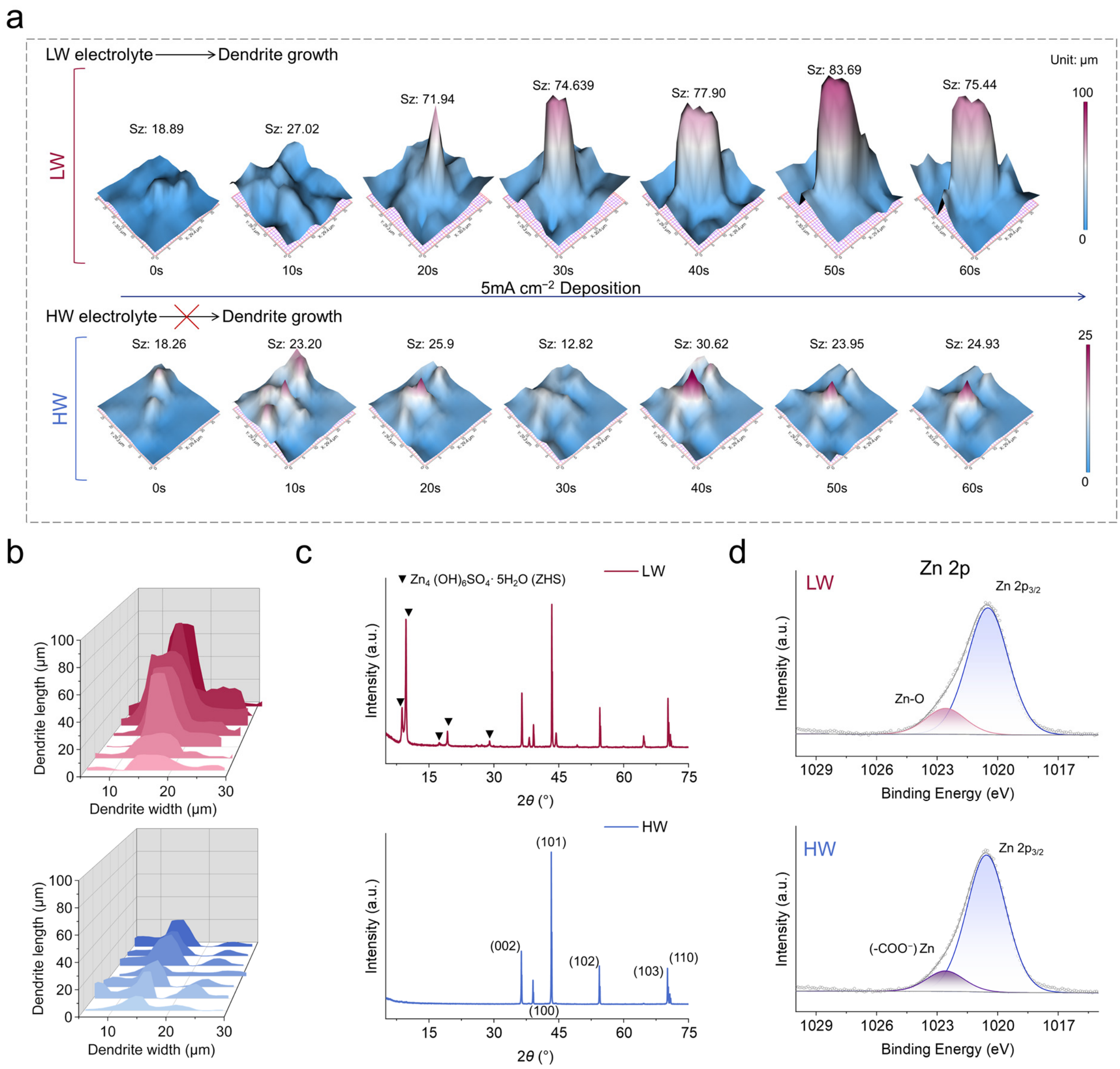

Supplementary Fig. 21 Three-dimensional reconstructed image (a) and cross-sectional profile (b) of in-situ dendrite growth. The images were obtained by in-situ laser confocal microscopy in a Zn||Zn (100 μm) symmetric cell under a current density of 5 mA cm^{-2}, with surface reconstruction performed at 10 s intervals. Post-deposition XRD (c) and XPS (d) spectra indicate the formation of basic zinc sulfate (ZHS) in the LW electrolyte. In contrast, the HW electrolyte containing the additive exhibits an interfacial protective layer with no detectable parasitic reaction products.

Original comment 2-5

The chemical analyses (XRD, XPS, ToF-SIMS) demonstrate the suppression of zinc hydroxide sulfate layers in high-W_f electrolytes, suggesting reduced parasitic reactions. However, the mechanistic link between W_f, interfacial chemistry, and suppression of side reactions is underdeveloped. In addition, the role of additive adsorption, solvation structure modifications, and double-layer restructuring are acknowledged but lack quantitative mechanistic insight. Notably, the long-term effects of local pH shifts, the evolution of interphases, and potential failure modes are not thoroughly characterized.

The authors might conduct in-depth interfacial studies under operando conditions, including in-situ X-ray absorption spectroscopy, to elucidate real-time interphase formation, chemical evolution, and their direct relationship with W_f. Furthermore, it would be better to study the influence of additive chemistries on interfacial kinetics and composition at multiple cycling stages.

Response

We appreciate the Reviewer's suggestion to elucidate the mechanistic connection between W_f and interfacial chemistry. Comprehensive chemical characterization, including XRD, XPS, and ToF-SIMS, confirms a clear correlation between elevated W_f values and the significant mitigation of zinc hydroxide sulfate (ZHS) formation. Specifically, ToF-SIMS data presented in **Fig. 4b** and **Supplementary Table 4** indicate the presence of an additive adsorption layer with a thickness of less than 3 nm. This layer provides physical evidence of a tripartite stabilization mechanism in which the concurrent effects of reactive-site blocking, solvation-shell modulation to inhibit Zn^{2+} hydrolysis, and double-layer restructuring collectively mitigate local ionic supersaturation. To substantiate this interpretation, we have incorporated additional experimental evidence, including cycle-resolved XPS across different stages (**Supplementary Fig. 32**) and long-term local (in-situ) pH measurements (**Supplementary Fig. 27**). We also included multi-cycle EIS(**Supplementary Fig. 32**) to track the evolution of charge-transfer and polarization resistance, and in-situ ELSCM (**Supplementary Fig. 21**). These complementary datasets demonstrate that high-W_f electrolytes effectively suppress parasitic surface chemistry. Crucially, they establish a statistical relationship between these chemical shifts and the kinetic resistance term in the W_f denominator, thereby validating the physical relevance of the descriptor.

We recognize that processes such as additive adsorption, solvation structure, and double-layer reconstruction play critical roles at the atomic scale in influencing side reactions and nucleation. However, the primary objective of this work is to propose and validate W_f as an engineering criterion based on experimentally accessible parameters, such as η, i_{corr}, and Z. This approach is intended to capture the collective interfacial behavior within a practical engineering framework rather than to replace dedicated atomistic mechanistic studies. The combined evidence from cycle-resolved XPS, EIS, and morphology statistics supports a quantitative correspondence between higher W_f values and effective suppression of side reactions.

We also concur that in situ X-ray absorption spectroscopy (XAS) at synchrotron facilities could provide valuable real-time chemical and morphological insight. As mentioned in Response to

Comment 2-4, such experiments require specialized cells, substantial beamtime allocation, and careful mitigation of X-ray beam effects in aqueous systems, including the use of thin electrolyte layers and low-dose data acquisition to ensure that the observed changes arise from electrochemical processes rather than radiation artifacts (*Chem. Rev. 2021, 121, 882-961; PCCP 2024, 26, 3897-3906*). Given these practical constraints, we prioritized the above multi-technique approach to produce reproducible, quantitative evidence within the scope of the present study. We are grateful for the Reviewer's suggestion, as the newly added data substantially strengthen the linkage between W_f, interfacial chemistry, and the suppression of side reactions.

In response to this comment, the relevant figures have been added as **Supplementary Figs. 27** and **32** in the **R-SI** (**pages 38** and **43**). In addition, corresponding descriptions have been included in the **R-MS** (**pages 10** and **12**):

'To further validate the long-term pH stability, in-situ local pH measurements (Supplementary Fig. 27) were performed during Zn deposition/stripping at 1 mA cm^{-2} for 6 h. The HW showed more stable pH fluctuations, with an average of 4.44, compared to 4.84 for the LW, confirming its superior pH stability.' in **R-MS (page 10)**

'To further investigate the evolution of interfacial chemistry and deposition/stripping kinetics during prolonged cycling, cells after 50 and 100 cycles were characterized using EIS, XRD, and XPS (Supplementary Fig. 32). The LW led to the formation of ZHS byproducts during cycling. In contrast, the HW maintained a more stable deposition/stripping interface.' in **R-MS (page 12)**

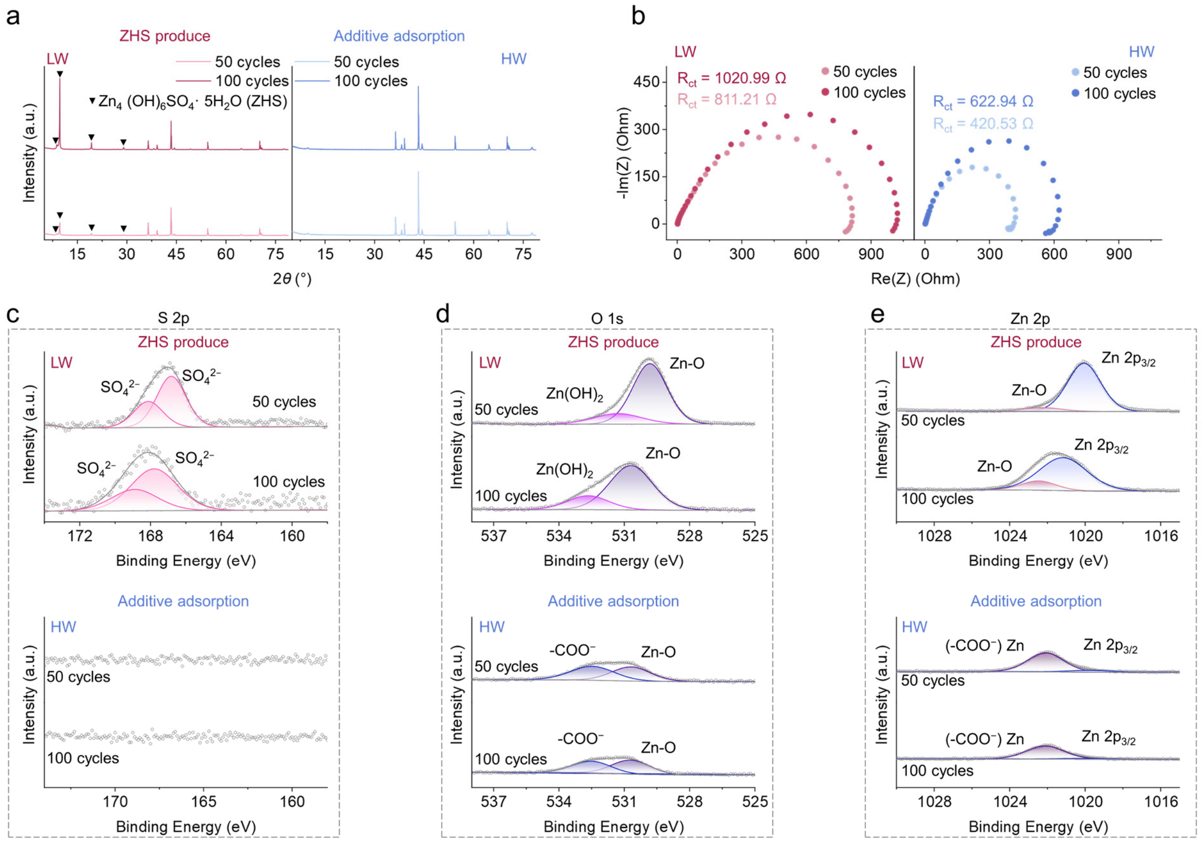

Supplementary Fig. 32 XRD (a), electrochemical impedance spectroscopy (EIS) (b), and XPS spectra of S 2p (c), C 1s (d), and Zn 2p (e) collected after 50 and 100 cycles for cells using LW and HW electrolytes. The cells were cycled at 1 mA cm^{-2} with an areal capacity of 1 mAh cm^{-2}. These measurements probe the evolution of interfacial dynamics and composition over multiple cycling stages and demonstrate the additive's adsorption-mediated protective effect on the electrode/electrolyte interface.

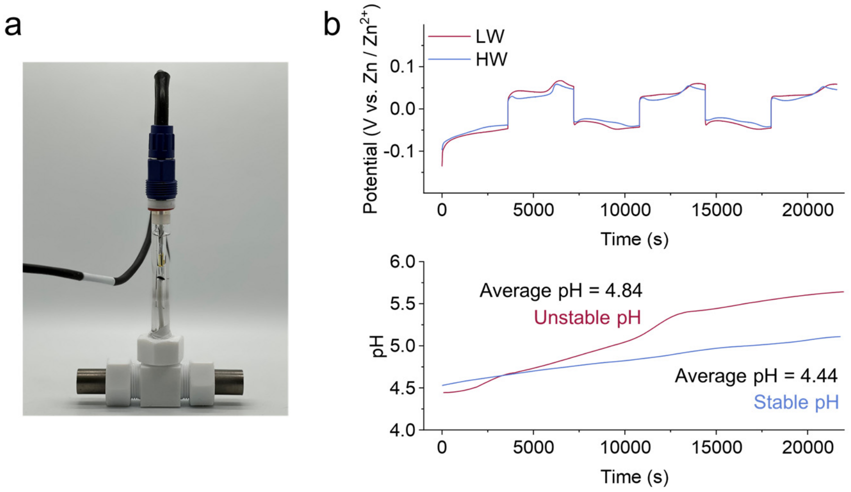

Supplementary Fig. 27 Optical image of the in-situ long-term effects of the local pH-shifts measurement setup. Voltage curves of Zn||Zn (100 μm) symmetric cells in (b) LW electrolyte and HW electrolyte at a current density of 1 mA cm^{-2}, together with the corresponding evolution of in-situ local pH.

Original comment 2-6

The in-situ 3D confocal microscopy (Fig. 3e) is a highlight, but the analysis is qualitative. Quantitative metrics such as surface fractal dimension or local curvature analysis would strengthen the conclusion regarding deposition uniformity.

Response

We appreciate the Reviewer's perceptive comment and agree that quantitative surface analysis significantly strengthens our conclusions regarding deposition uniformity. To address this point, we utilized a home-made specialized technology involving in-situ 3D Electrochemical Laser Scanning Confocal Microscope (ELSCM) (**Fig. 3e**). We performed detailed surface characterization through two widely recognized metrology methods, including Abbott Firestone bearing area curve analysis in **Supplementary Fig. 20a** and power spectral density (PSD) analysis in **Supplementary Fig. 20b**. Specifically, the Abbott-Firestone method quantifies the proportion of surface area at various height levels, and the PSD analysis characterizes the distribution of surface-height variance across spatial frequencies. The parameters extracted from these comprehensive analyses are summarized in **Supplementary Table 3**. These results demonstrate that the HW electrolyte enables a more uniform distribution of electrode surface heights and a more homogeneous spatial arrangement of surface

features during deposition. We believe that incorporating these additional quantitative metrics provides a more robust and objective evaluation of electrode surface uniformity.

In response to this comment, the relevant figure has been added as **Supplementary Fig. 20** in **R-SI (page 31)**, and the relevant table has been added to **Supplementary Table 3** in **R-SI (page 52)**. Moreover, an additional description has also been included in **R-MS (page 8)**:

'Abbott-Firestone bearing area curve analysis and power spectral density (PSD) analysis (Supplementary Fig. 20) reveal that the HW yields a more uniform electrode interface height and a more uniform spatial distribution of deposited features during Zn deposition (Supplementary Table 3).' in **R-MS (page 8)**

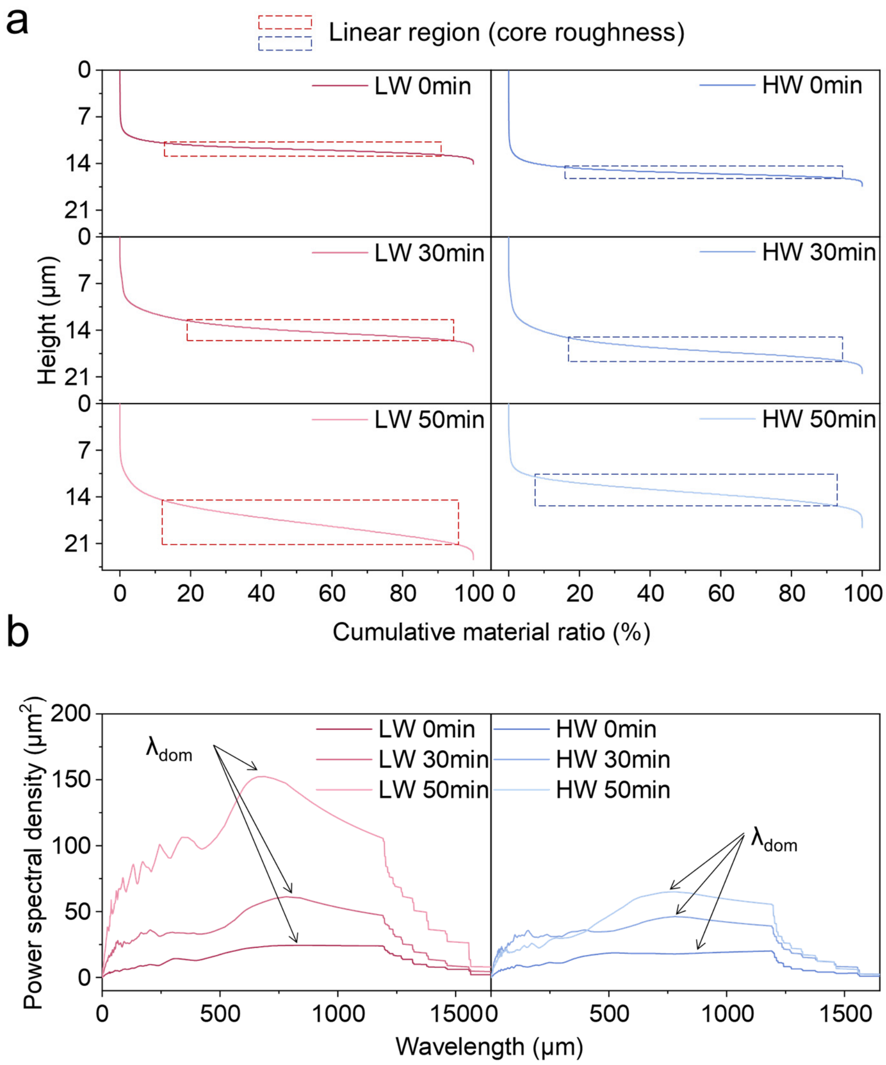

Supplementary Fig. 20 (a) Abbott–Firestone bearing area curve analysis showing the core roughness depth (Sk), together with the reduced peak height (Spk), reduced valley depth (Svk), and the upper and lower material ratios (Mr1 and Mr2), which collectively quantify the height distribution and uniformity of Zn deposition. (b) Power spectral density (PSD) analysis of the surface height profile, from which the dominant spatial wavelength (λ_{dom}) and the maximum PSD amplitude ($A_{PSD,max}$) are extracted to characterize the characteristic length scale and intensity of surface height fluctuations.

Supplementary Table 3 Data obtained from Abbott-Firestone bearing area curve analysis and power spectral density (PSD) analysis.

Surface texture parameters	LW-0min	LW-30min	LW-50min	HW-0min	HW-30min	HW-50min
Sz (maximum height of the surface, μm)	5.24	11.00	14.52	5.42	9.73	10.49
Sa (arithmetical mean height, μm)	0.58	1.48	2.01	0.58	1.24	1.2
Sk (core roughness depth, μm)	1.7	3.0	6.7	1.6	3.4	4.1
Spk (reduced peak height, μm)	1.5	3.1	3.7	1.6	3.4	2.0
Svk (reduced valley depth, μm)	0.5	0.6	0.9	0.4	0.7	1.0
Mr1 (upper material ratio, %)	13.9	19.4	11.8	16.2	17.9	9.7
Mr2 (lower material ratio, %)	91.3	94.5	95.2	93.6	94.2	92.2
λ_{dom} (dominant wavelength, μm)	843.3	780.3	690.6	851.2	780.3	776.9
$A_{PSD,max}$ (maximum PSD amplitude, μm^2)	4.9	7.8	12.3	4.5	6.8	8.1

Response to Reviewer #3

Remarks to the Author

This manuscript investigates, from the perspective of interfacial electrochemistry, the nucleation and growth behavior of zinc during electrodeposition in aqueous zinc-ion batteries. By combining theoretical analysis with a variety of electrochemical measurements and structural characterizations, the authors establish correlations between interfacial kinetic parameters and deposition stability. The descriptors proposed in the manuscript have a clear physical basis and offer valuable insight and guidance for understanding zinc-anode failure mechanisms and for designing electrolyte/interface regulation strategies. The work demonstrates high novelty with complete supports; the experimental and analytical results can support the authors' main conclusions. I think the manuscript is suitable for publication in *Nature Communications* and recommend acceptance after minor revision.

Response

We appreciate Reviewer #3 for the thoughtful and constructive feedback, which helped us clarify and strengthen the presentation of our study. All comments have been addressed in detail, and the revised manuscript (**R-MS**) and Supplementary Information (**R-SI**) have been submitted to *Nature Communications*.

Original comment 3-1

The manuscript primarily emphasizes interfacial electrochemical regulation (e.g., additive adsorption, reduction of γ, changes in R_{ct}, and suppression of corrosion current), while bulk electrolyte properties such as ionic conductivity and diffusion coefficients are not adequately addressed. The authors should include basic characterizations of the electrolyte bulk phase and briefly discuss how these properties may influence the observed interfacial behavior.

Response

We thank the Reviewer for the valuable comment and have provided a comprehensive characterization of the liquid-phase properties for both LW and HW electrolytes at room temperature. Ionic conductivities were measured with an ion conductivity meter (FE38, Mettler Toledo). The HW electrolyte exhibited a conductivity of 125.6 mS cm^{-1}, which represents a clear improvement over the 118.1 mS cm^{-1} recorded for the LW electrolyte, as shown in **Supplementary Fig. 22**. Furthermore, we evaluated the diffusion coefficients using the Zn||Cu asymmetric cells. We presented the corresponding results in **Supplementary Fig. 23**. Specifically, the diffusion coefficient for the HW electrolyte reached 1.03×10^{-5} cm^2 s^{-1}, while that of the LW electrolyte was 7.97×10^{-6} cm^2 s^{-1}.

These results indicate that the superior ionic conductivity and higher Zn^{2+} diffusion coefficient of the HW system significantly enhance both charge and mass transport kinetics. The improved conductivity reduces ohmic drop and promotes a more uniform current distribution across the electrode surface. Simultaneously, the higher diffusion coefficient accelerates Zn^{2+} replenishment and effectively alleviates concentration polarization during electrodeposition. These synergistic factors work together to suppress current localization and tip-enhanced growth, which ultimately leads to more rapid and uniform zinc electrodeposition. We have incorporated these transport parameters and the

related discussion into the Revised Manuscript to provide a more complete physical picture of the electrolyte performance.

In response to this comment, the relevant figures have been added as **Supplementary Figs. 22** and **23** in **R-SI (pages 33** and **34)**. Moreover, the additional descriptions have also been included in **R-MS (page 10)** and **R-SI (page 34)**:

'Such electrolytes also demonstrate higher bulk ionic conductivity (Supplementary Fig. 22) and larger diffusion coefficients (Supplementary Fig. 23).' in **R-MS (page 10)**

'Based on the simplified Randles-Sevcik equation (Supplementary Equation (47)), the Zn^{2+} diffusion coefficient ($D_{Zn^{2+}}$) was determined by fitting the peak currents obtained from cyclic voltammetry measurements of Zn||Cu asymmetric cells at varying scan rates.

$$I_p = 269000 n^{\frac{3}{2}} A C D^{\frac{1}{2}} v^{\frac{1}{2}} \tag{47}$$

where D represents the ion diffusion coefficient, I_p is the peak current, v denotes the scan rate, C is the concentration of electrolyte ions, n is the number of electrons transferred in the redox reaction, and A is the electrode area.' in ***R-SI (page 34)***

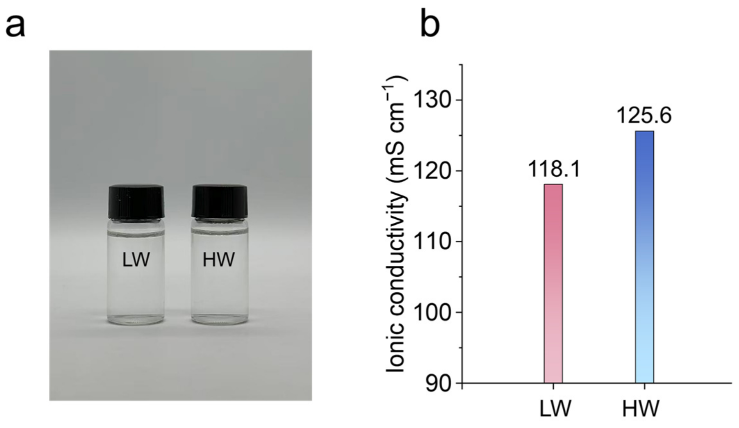

Supplementary Fig. 22 Optical images of the LW electrolyte and HW electrolyte (a) and ionic conductivity (b).

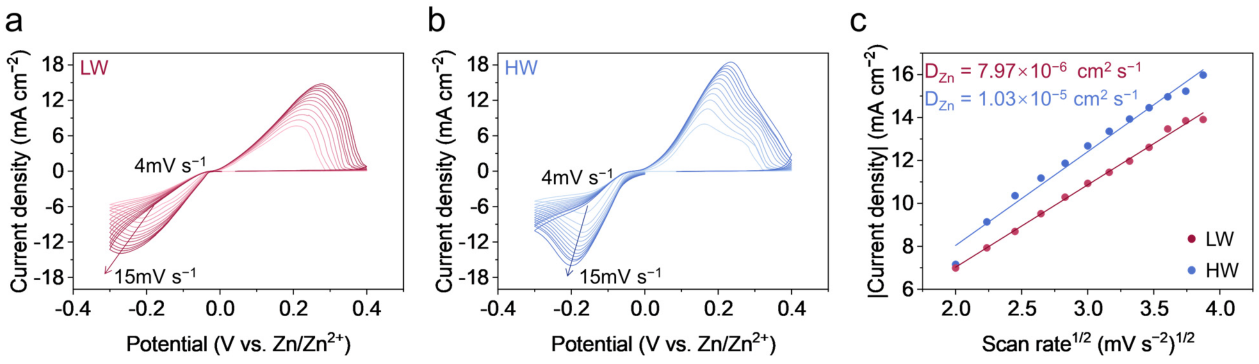

Supplementary Fig. 23 The CV tests of Zn||Cu asymmetric cells with (a) LW electrolyte (0.05 M $ZnSO_4$) and (b) HW electrolyte (15 mM AT-0.05 M $ZnSO_4$), and the corresponding diffusion coefficient calculated from simplified Randles-Sercik curves. The scan rate is from 4 to 15 mV s^{-1}.

Original comment 3-2

Some parameters require clarification. In particular, Table S2 lists the system temperature T0 = 200 K, which is inconsistent with room-temperature experiments. Please confirm the simulation temperature and indicate whether this is a typographical error. The authors should also provide basic modeling details, including boundary and initial conditions, mesh size and convergence checks, and whether Butler-Volmer kinetics are coupled, to ensure the simulations are physically meaningful and reproducible.

Response

We sincerely thank the Reviewer for the meticulous review and for identifying this inconsistency. We clarify that the entry T0 = 200 K in Table S2 was a typographical error because all simulations in this study were performed at a standard room temperature of 298 K. We have corrected this value in the **R-SI** and ensured that all related parameters remain consistent throughout the manuscript.

To ensure the physical rigor and reproducibility of our computational results, we have provided comprehensive modeling details in the revised document. These additions include explicit descriptions of the boundary and initial conditions, as well as mesh independence tests and convergence verification. We also elaborated on the specific coupling of the electrochemical interface with Butler-Volmer kinetics to further justify the simulation framework. These revisions adequately address the concerns regarding the modeling methodology and strengthen the overall quality of the study.

In response to this comment, **Table S2** has been corrected in the **R-SI (page 51)**. Moreover, the additional description has also been included in **R-SI (pages 10 and 11)**:

*'The electric-field distribution and morphological evolution of metal electrodes (Li and Zn) during deposition were simulated in COMSOL Multiphysics 6.2 using physics-based module [15]. The model geometry was constructed from experimentally measured dimensions. Ion transport was described by the Nernst-Planck equations coupled with mass and charge conservation, and interfacial charge transfer was implemented using Butler-Volmer kinetics with the Faradaic flux coupled to species transport at the electrode-electrolyte interface. A three-dimensional (3D) transient current distribution model was coupled to a geometric-deformation module to capture the dynamic interplay between electrochemical deposition and surface morphology, and the electric-field distribution was calculated using COMSOL's Electric Field interface. The stripping electrode potential was fixed at 0 V, while the depositing electrode was treated as a conductive boundary with a prescribed equilibrium potential or an imposed normal surface current density. Transient simulations employed an implicit BDF scheme with adaptive time stepping. Mesh refinement was applied near interfaces, and mesh independence was confirmed by observing that key observables varied by less than 2%. Material properties were taken from the COMSOL library, while the ionic diffusion coefficients (**Supplementary***

*Fig. 23) and charge transfer coefficients (**Supplementary Fig. 9**) were adopted from experimental measurements.' in **R-SI (pages 10 and 11)***

Supplementary Table 2 Simulated electrolyte parameters in 1M $ZnSO_4$ H_2O solution[18].

Param Name	Value and Unit	Description
Cinit	1000 (mol m^{-3})	Initial concentration
T0	298 (K)	System temperature
i0_ref	1^{-6} (A m^{-2})	Exchange current density for reference conditions
phis_anode	0.1 (V)	Anode potential
phis_cathode	−0.1 (V)	Cathode potential
alpha_a	1.5	Symmetry factor
z_Zn	2	Charge, species Zn^{2+}
z_SO4	−2	Charge, species SO_4^{-2}
D_Zn	1^{-9} (m^2 s^{-1})	Diffusivity, species Zn^{2+}
D_SO4	1^{-9} (m^2 s^{-1})	Diffusivity, species SO_4^{-2}

Original comment 3-3

The Supplementary Information indicates that XPS depth profiling was performed using 4 keV Ar^+ sputtering and that ToF-SIMS used 2 keV Ar^+. The authors should provide sputtering-rate calibration data (in nm s^{-1}) based on a reference or standard sample, and briefly discuss possible sputtering-induced effects such as chemical reduction or elemental migration, particularly for zinc valence states and oxygen-containing species, so that readers can assess the quantitative reliability of the depth profiles.

Response

We appreciate the Reviewer's valuable and constructive suggestion, which has substantially improved the quantitative rigor of our depth profile analysis. To address this point, we have incorporated sputter rate calibration data derived from reference samples into **Supplementary Table 4** (*J. Vac. Sci. Technol. A. 2010, 28, 1060-1072; Jpn. J. Appl. Phys. 2005, 44, 7555; Commun. Chem. 2025, 8, 31*). Accordingly, we have included a focused discussion regarding the potential reduction of sulfur valence states during the sputtering process, as well as the oxidation and reduction states of Zn, as shown in **Supplementary Fig. 25**. These additions allow for a more critical evaluation of the quantitative reliability of our depth profiles and help to address potential artifacts associated with ion beam interactions. We believe that these refinements provide readers with a clearer understanding of the interfacial chemistry and strengthen the overall validity of our conclusions.

In response to this comment, the relevant figure has been added as **Supplementary Fig. 25** in **R-SI (page 36)**, and the relevant table has been added to **Supplementary Table 4** in **R-SI** (**page 53**). Moreover, an additional description has also been included in **R-MS (page 10)**:

'This conclusion is further supported by the Zn 2p spectra, which show metallic Zn peaks in the HW after 2 min of sputtering (about 20 nm). In contrast, the LW displays only Zn-O bonds throughout the depth profile (Supplementary Fig. 25)[52]*.' in* ***R-MS (page 10)***

'Sulfite and sulfide signals likely result from Ar^+ bombardment, where preferential sputtering induces oxygen depletion and concurrent electron bombardment and localized heating promote S-O bond reduction, partially converting the original sulfate (SO_4^{2-}) into lower-valence sulfur species (SO_3^{2-} and S^{2-}).' in ***R-MS (page 10)***

Supplementary Table 4 Sputtering conditions and calibrated sputtering rates for XPS depth profiling and ToF-SIMS analysis.

Technique	Beam type / energy	Reference sample (matrix)	Reported sputter rate (nm s^{-1})	Citation
XPS depth profiling	Ar^+, 4 keV	Ta_2O_5 (on Si)	0.5 nm·s^{-1}	19
	Ar^+, 4 keV	ZnO (insulating film)	≈ 0.167 nm·s^{-1} (≈ 10 nm·min^{-1})	20
ToF-SIMS	Ar^+, 500 eV	PVD lithium reference (30 nm Li on Cu)	0.246 ± 0.03 nm·s^{-1} (500 eV Ar^+ @20 nA)	21
	Ar_{1500}^+, 5 keV	Li reference (30 nm Li on Cu)	(6.04 ± 0.13) × 10^{-2} nm·s^{-1}	
	Ar_{1500}^+, 10 keV	Li reference (30 nm Li on Cu)	2.69 ± 0.13 nm·s^{-1}	

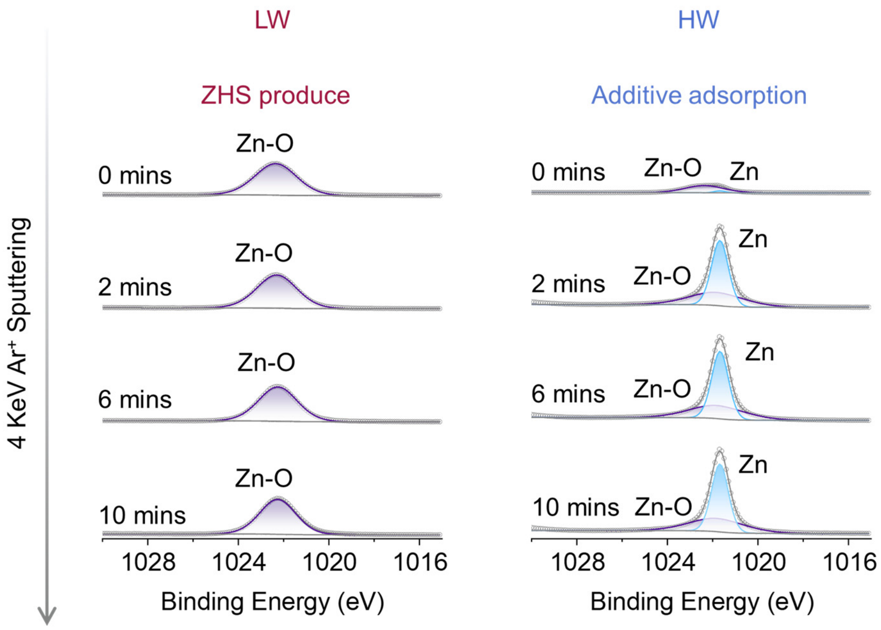

Supplementary Fig. 25 Sputtering XPS spectra of Zn 2p of Zn metal following 5 days of immersion in LW and HW electrolytes.

Original comment 3-4

On pages 3 and 5, the subsection titles "Classical nucleation theory for Zn metal" and "Validation of the Zn classical nucleation theory" would benefit from revision because they do not clearly highlight the specific focus of each section and may inadvertently discourage careful reading. The authors are encouraged to revise these titles better to reflect the key emphasis of the respective sections.

Response

We sincerely appreciate the Reviewer's helpful suggestion. The subsection titles on **pages 3** and **5** of the **R-MS** have been revised to more clearly reflect the specific focus and key emphasis of each section.

In response to this comment, the subsection titles have been revised to more clearly reflect the specific focus of each section in the **R-MS (page 3** and **page 5)**:

'A Zn-HCP nucleation theory' in ***R-MS (page 3)***

'Experimental validation of the Zn-HCP nucleation theory' in ***R-MS (page 5)***

Original comment 3-5

In Figures 4c and 4d, the XPS data treatment appears inappropriate: the S-related peaks should be deconvoluted using a doublet-fitting approach. The authors should reprocess the XPS data and revise the analysis accordingly.

Response

We thank the Reviewer for their careful reading and valuable comments. All relevant corrections have been incorporated into the **R-MS**.

In response to this comment, the relevant figures have been added as **Figs. 4c** and **4 d** in **R-MS** (**page 11**). Moreover, an additional description has also been included in **R-MS** (**page 10**):

'To further elucidate the underlying chemical states, depth-profiling X-ray photoelectron spectroscopy (XPS) is conducted after 5 days of immersion. In the LW, after 10 min of 4 keV Ar^+ sputtering, the O 1s spectrum shows pronounced peaks at 532.2 and 530.5 eV, and the S 2p region displays sulfate-related features, consistent with the formation of a compact ZHS layer (Fig. 4c and Fig. 4d). By contrast, no sulfate-related signals are detected for the HW electrolyte under the same sputtering conditions. Only features attributable to an adsorbed tartrate layer are observed without evidence of ZHS formation.' in ***R-MS (page 10)***

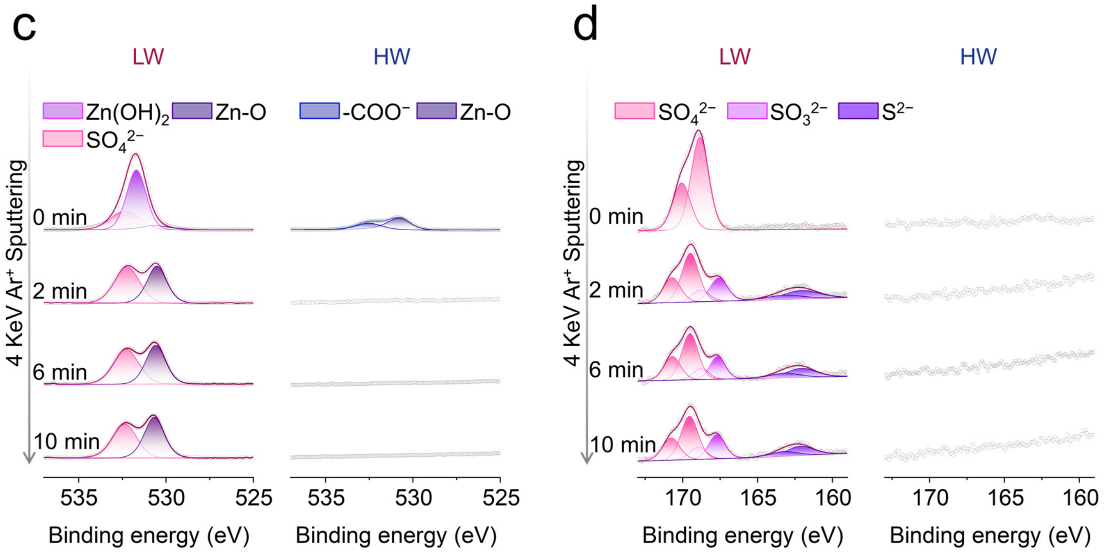

Figure 4. Interfacial behavior and electrochemical performance of LW and HW electrolytes. Depth-profiling X-ray photoelectron spectroscopy (XPS) spectra of (**c**) O1s and (**d**) S2p of Zn metal following 5 days of immersion in LW and HW electrolytes.

END OF RESPONSE TO REVIEWS
